# Multi-target network modulation of Traditional Chinese Medicine in radiation enteropathy: mechanistic insights and therapeutic implications

**DOI:** 10.1186/s13020-026-01504-y

**Published:** 2026-11-02

**Authors:** Biyu Tan, Xiaofang Chen, Gaoxuan Shao, Hang Su, Xian Ma, Xing Ni, Lu Lu, Zemin Lin, Guang Ji, Hanchen Xu

**Affiliations:** 1 The Second Clinical Medical College, Guizhou University of Traditional Chinese Medicine, Guiyang, 550002 China; 2 Institute of Digestive Diseases, Longhua Hospital, China-Canada Center of Research for Digestive Diseases (ccCRDD), Shanghai University of Traditional Chinese Medicine, 725 South Wanping Road, Shanghai, 200032 China; 3 State Key Laboratory of Integration and Innovation of Classic Formula and Modern Chinese Medicine (Shanghai University of Traditional Chinese Medicine), Shanghai, 200032 China; 4 Shanghai Frontier Research Center of Disease and Syndrome Biology of Inflammatory Cancer Transformation, Shanghai, 200032 China

**Keywords:** Radiation enteropathy, Traditional Chinese Medicine, Signaling pathways, Gut microbiota, Oxidative stress, Epithelial barrier

## Abstract

Radiation enteropathy (RE) is a common and clinically significant complication of abdominal and pelvic radiotherapy, characterized by multifactorial pathological processes including oxidative DNA damage, epithelial barrier disruption, immune–inflammatory dysregulation, vascular injury, fibrosis, and gut microbiota dysbiosis. Despite its clinical burden, effective disease-modifying therapies remain limited. Traditional Chinese Medicine (TCM), widely used in the management of gastrointestinal disorders, has attracted increasing attention as a complementary approach for radiation-induced intestinal injury. Accumulating experimental evidence indicates that TCM formulas, herbal extracts, and bioactive phytochemicals may exert protective effects through coordinated regulation of interconnected pathological processes. Reported protective effects involve several interconnected pathological processes, but the supporting evidence remains uneven. Most studies use acute, single-dose animal models, and many mechanistic conclusions rely on pathway-marker changes rather than functional perturbation. This review therefore evaluates TCM interventions according to disease stage, intestinal site, irradiation design, and the strength of mechanistic support. Multi-target regulation is treated as an interpretive framework rather than a mechanism established for every intervention. Clinical evidence remains limited by small samples, heterogeneous preparations, inconsistent endpoints, and inadequate follow-up. Translation will require standardized products, clinically relevant fractionated and tumor-bearing models, exposure-relevant mechanistic validation, and rigorous trials that assess intestinal benefit without compromising tumor control.

**Supplementary Information:**

The online version contains supplementary material available at https://doi.org/10.1186/s13020-026-01504-y.

## Introduction

Radiation enteropathy (RE), also known as radiation enteritis or radiation-induced intestinal injury, is a frequent complication of radiotherapy for abdominal and pelvic malignancies, primarily affecting the small intestine, colon, and rectum [1, 2]. More than 75% of patients receiving abdominal or pelvic RT develop RE to varying degrees [3]. Based on radiation dose, exposure duration, and time of onset, RE is classified into acute radiation enteropathy (ARE) and chronic radiation enteropathy (CRE) [4, 5]. ARE typically occurs during RT or within three months after treatment and presents with diarrhea, abdominal pain, bleeding, and tenesmus [6, 7], affecting approximately 60–80% of patients depending on irradiation site, technique, and individual susceptibility [8]. In contrast, CRE develops months to decades after RT, with latency periods reported up to 30 years [6, 9], and affects more than 20% of patients undergoing pelvic or abdominal irradiation [10–12]. CRE is characterized by progressive obliterative endarteritis and submucosal fibrosis, leading to severe complications such as strictures, fistulae, abscesses, perforation, and chronic bleeding, which may be fatal [13]. With the increasing global cancer burden and widespread application of RT in pelvic malignancies, the incidence of RE continues to rise. RE disrupts treatment continuity, significantly impairs quality of life, and negatively impacts oncological outcomes, as radiotherapy interruptions reduce local tumor control rates by approximately 1–1.4% [7, 14, 15].

The pathogenesis of RE is multifactorial, involving epithelial injury, immune dysregulation, and intestinal microenvironmental alterations. Radiation directly damages intestinal epithelial stem cells, impairing mucosal regeneration [15], and disrupts epithelial tight and adherens junctions, resulting in barrier dysfunction and mucosal thinning [16–18]. Concurrent vascular injury induces ischemia and thrombosis, further exacerbating mucosal necrosis [19]. Barrier disruption facilitates microbial translocation and perpetuates local inflammation. Immune dysregulation is a central driver of disease progression: radiation activates macrophages and other immune cells, inducing sustained release of pro-inflammatory cytokines such as TNF-α and IL-6 [20, 21]. Damage to Paneth cells compromises antimicrobial defense, contributing to endotoxemia and systemic inflammation [16]. In parallel, radiation-induced cellular injury releases damage-associated molecular patterns (DAMPs) [22], activating innate immune pathways and amplifying adaptive immune responses [23, 24], resembling immune disturbances observed in HIV-associated enteropathy [25]. Gut microbiota dysbiosis further aggravates RE, characterized by depletion of beneficial bacteria and short-chain fatty acids (SCFAs), alongside expansion of pathogenic species such as *Escherichia coli* [18]. Reduced SCFA production compromises anti-inflammatory signaling and epithelial repair, whereas disturbances in mucin metabolism, partly involving *Akkermansia muciniphila*, promote bacterial translocation and contribute to systemic inflammation [8, 18, 26]. Emerging evidence also implicates oxidative stress and ferroptosis in radiation-induced gastrointestinal injury, suggesting an additional layer of redox imbalance within the intestinal microenvironment [27].

Current clinical management of RE relies largely on symptomatic interventions, including pharmacotherapy, surgery, and nutritional support. However, effective disease-modifying treatments remain limited, and existing strategies are often associated with prolonged treatment courses, suboptimal efficacy, adverse effects, and high costs [28, 29]. Thus, alternative and complementary therapeutic approaches are urgently needed [30]. Traditional Chinese Medicine (TCM), with extensive clinical experience in gastrointestinal disorders, has increasingly attracted attention as a complementary therapeutic strategy for RE. Its multi-component composition provides a plausible basis for effects across several interconnected pathological domains; however, the cross-study pattern of “multi-target regulation” should be treated as an interpretive framework rather than as a mechanism universally demonstrated for all TCM interventions [31–33]. Accumulating preclinical evidence suggests that TCM interventions may protect intestinal barrier integrity and attenuate inflammatory injury in radiation-induced enteropathy [31–33].

Earlier reviews largely emphasized general pathogenesis and management, natural-product or TCM intervention catalogues, or selected domains such as intestinal stem-cell signaling [1, 30, 34, 35]. A recent TCM-focused review further compared preclinical and clinical evidence and discussed broad methodological and translational limitations [36]. Building on these syntheses, the present review adopts a more focused appraisal framework that links injury stage, intestinal compartment, irradiation design, and depth of mechanistic validation. This framework organizes RE as a continuous pathological network spanning oxidative injury, barrier disruption, inflammatory and microbial amplification, and fibrotic remodeling, while distinguishing acute from chronic disease and small-intestinal from colonic or rectal evidence. The review further differentiates hypothesis-generating predictions and association-based findings from pathway perturbation and more direct causal validation, without treating network pharmacology or molecular docking as proof of mechanism. The review also considers clinical and translational issues, with particular attention to administration routes and outcome measures, concurrent evaluation of intestinal protection and tumor response during radiotherapy, and the standardization and reproducibility of TCM preparations.

Accordingly, after defining the literature search strategy and review scope, we examine how acute epithelial injury, incomplete repair, vascular dysfunction, microbial and inflammatory amplification, and fibrotic remodeling contribute to RE. We then evaluate TCM formulas, herbal preparations, extracts, and defined compounds according to disease stage, anatomical site, irradiation model, and strength of mechanistic support. The final sections discuss the limitations of the current evidence and priorities for clinical translation, with particular attention to tumor safety, preparation standardization, dose translation, intestinal exposure, and reproducibility.

## Literature search and review scope

Literature identification and synthesis followed a structured narrative approach. The databases were searched from inception through July 18, 2026. Searches were conducted in three international bibliographic databases (PubMed, Web of Science Core Collection, and Embase) and three Chinese bibliographic databases (China National Knowledge Infrastructure [CNKI], Wanfang Data, and SinoMed). Search syntax was adapted to the indexing systems and search functions of each database.

The core search combined terms related to radiation-induced intestinal injury with terms related to TCM and herbal interventions. Representative disease-related terms included “radiation enteropathy,” “radiation enteritis,” “radiation-induced intestinal injury,” “radiation proctitis,” “radiation proctopathy,” “radiation colitis,” “radiation-induced intestinal fibrosis,” and “gastrointestinal acute radiation syndrome.” Intervention-related terms included “Traditional Chinese Medicine,” “Chinese herbal medicine,” “herbal formula,” “decoction,” “Chinese patent medicine,” “herbal preparation,” “herbal extract,” “medicinal plant,” “phytochemical,” and “bioactive compound,” together with relevant controlled vocabulary, synonyms, and corresponding Chinese terms, where applicable. Targeted supplementary searches were conducted for specific formulas, extracts, and compounds identified during the initial search and, where necessary, for mechanistic and translational questions addressed in this review. Reference lists of relevant original studies and reviews were also screened to identify additional eligible publications.

Original in vitro studies (including organoid studies), in vivo studies, and clinical studies were eligible if they directly investigated radiation-induced intestinal injury or clinically defined radiation enteropathy and evaluated a TCM formula, Chinese patent medicine, multi-component herbal preparation, medicinal plant extract or fraction, or chemically defined bioactive compound of herbal origin. Eligible studies were required to report outcomes related to intestinal injury, epithelial survival or regeneration, barrier integrity, inflammatory or immune responses, vascular injury or fibrotic remodeling, gut microbiota, clinical symptoms or radiation toxicity, quality of life, treatment response, or safety. Publications in English and Chinese were considered. Master’s and doctoral dissertations identified through the Chinese databases were included only when the full text was available, the methods and outcomes were adequately reported, and no peer-reviewed publication reporting the same data could be identified.

Studies were excluded if they addressed non-radiation-related intestinal diseases, radiation injury confined to non-intestinal organs, or interventions outside the defined scope of TCM and medicinal plant-derived therapies. Duplicate reports and publications with insufficient methodological or outcome information were excluded, as were editorials, commentaries, conference abstracts, and protocols without outcome data. Network pharmacology, in silico prediction, and molecular docking studies without experimental or clinical validation were not treated as stand-alone evidence of an established mechanism. Where relevant, their findings were considered exploratory and hypothesis-generating. Reviews and meta-analyses were used for contextual synthesis and reference tracking but were not regarded as primary intervention evidence.

For the analysis of RE pathobiology, priority was given to original mechanistic studies directly investigating radiation-induced intestinal injury. Evidence from non-RE settings was considered only when it informed specific translational questions, including potential effects on tumor response to radiotherapy, pharmacokinetics, chemical characterization, safety, or product standardization, and was interpreted as indirect or contextual evidence. The clinical evidence included in this review comprised case reports, a prospective single-arm study, and randomized controlled trials; no non-randomized controlled studies were included.

Eligible studies were characterized descriptively according to study design, experimental system, irradiation setting and regimen, injury stage, intestinal site, intervention dose, route and timing of administration, reported outcomes, and the nature and extent of mechanistic validation. As this was a structured narrative review rather than a systematic review or meta-analysis, no formal risk-of-bias assessment, quantitative evidence synthesis, or standardized evidence-grading framework was applied.

## Pathogenesis of RE

### Radiation-induced DNA damage and oxidative stress

Ionizing radiation induces multiple forms of DNA damage, including single-strand breaks (SSBs), double-strand breaks (DSBs), base lesions, and DNA crosslinks, among which DSBs and oxidative base damage are the most deleterious [37–40]. DNA damage activates a highly coordinated cellular stress response encompassing DNA repair, cell-cycle arrest, and apoptosis [41]. Central to this response are the phosphatidylinositol 3-kinase–related kinases ataxia-telangiectasia mutated (ATM) and ataxia telangiectasia and Rad3-related (ATR), which are preferentially activated by DSBs and single-stranded DNA, respectively [42–47]. When the extent of DNA damage exceeds repair capacity, prolonged checkpoint activation results in irreversible cell-cycle arrest or apoptosis [38, 48].

Radiation-induced DNA damage is tightly coupled to oxidative stress. Beyond the transient ROS generated by water radiolysis, the DNA damage response can sustain enzyme-dependent ROS production. In genotoxic-stress models, H2AX-dependent signaling enhances Rac1–NOX1 activity, partly by reducing the inhibitory interaction between 14–3-3ζ and NOXA1 [49]. Consistent with the intestinal relevance of this pathway, low-dose irradiation induces persistent upregulation of NOX1-associated components and enhances Rac1–NOX1 interaction in the mouse colon [50]. Mitochondrial dysfunction and dysregulated iron metabolism further amplify ROS generation [51, 52], leading to lipid peroxidation and accumulation of malondialdehyde (MDA) [53]. MDA forms covalent adducts with proteins and aminophospholipids, thereby disrupting macromolecular function and compromising membrane integrity [54]. Conversely, excessive ROS can impair the activity of DNA repair enzymes such as OGG1, weakening genomic maintenance and perpetuating DNA damage [48]. This bidirectional reinforcement between oxidative stress and DNA damage, to which p53-dependent signaling may contribute, accelerates cellular injury [55].

**Fig. 1:**
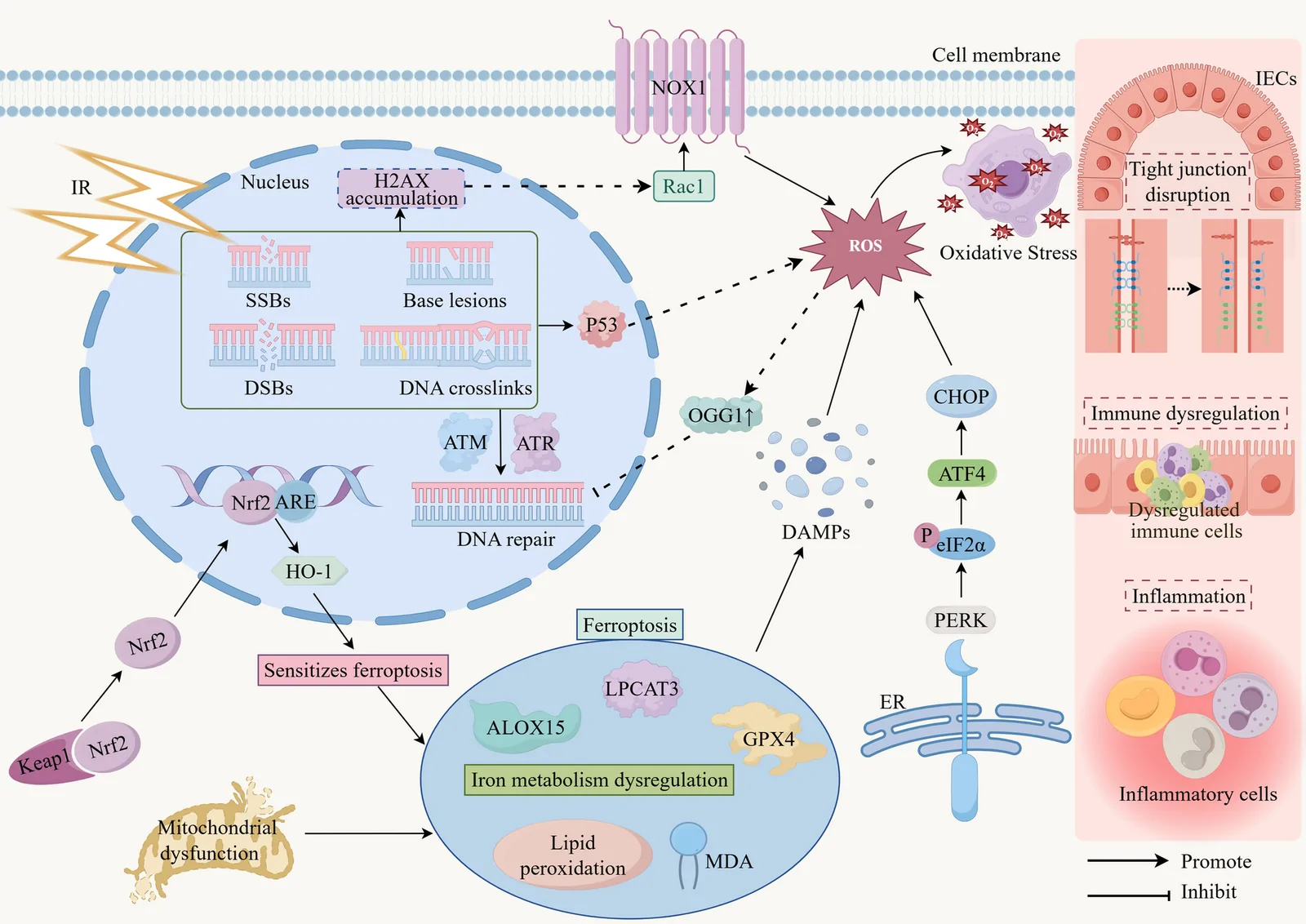
Radiation-induced DNA damage and oxidative stress. Ionizing radiation causes DNA lesions and H2AX-Rac1-NOX1-dependent ROS generation; mitochondrial dysfunction, impaired DNA repair, Nrf2/HO-1 and PERK-eIF2α-ATF4/CHOP signaling, and iron-dependent lipid peroxidation converge on ferroptotic epithelial injury, barrier dysfunction and inflammation. Solid arrows indicate activation or promotion; blunt-ended lines indicate inhibition; dashed arrows indicate indirect or putative relationships; P denotes phosphorylation; and ↑ indicates increased expression or activity. Key abbreviations: IR, ionizing radiation; ROS, reactive oxygen species; DSBs/SSBs, double-/single-strand breaks; H2AX, histone H2AX; NOX1, NADPH oxidase 1; Nrf2, nuclear factor erythroid 2-related factor 2; HO-1, heme oxygenase 1; PERK, protein kinase R-like endoplasmic reticulum kinase; eIF2α, eukaryotic initiation factor 2α; ATF4, activating transcription factor 4; CHOP, C/EBP homologous protein; GPX4, glutathione peroxidase 4; DAMPs, damage-associated molecular patterns

Ferroptosis represents an important downstream consequence of radiation-induced oxidative stress. Radiation promotes iron-dependent lipid peroxidation, accompanied by increased expression of ferroptosis-related factors, including ALOX15 and LPCAT3, while concurrently suppressing the GPX4 antioxidant system [51, 53, 55–57]. Dysregulation of the Nrf2/HO-1 axis further sensitizes cells to ferroptotic injury [58, 59], whereas endoplasmic reticulum stress may modulate ferroptosis through PERK-mediated eIF2α phosphorylation and ATF4 signaling [60]. Ferroptotic epithelial cell death may further aggravate barrier injury and contribute to inflammatory signaling through the release of damage-associated signals [51]. The resulting interplay between lipid peroxidation and inflammatory signaling may further intensify oxidative and epithelial injury, thereby contributing to epithelial dysfunction and persistent inflammation in RE [61] **(**Fig. 1**)**.

### Intestinal epithelial barrier dysfunction

The intestinal epithelium constitutes a multifaceted barrier system that integrates physical, chemical, microbial, and immune components to maintain intestinal homeostasis. Radiation preferentially targets leucine-rich repeat-containing G-protein-coupled receptor 5 (LGR5)-positive intestinal stem cells (ISCs) located at the crypt base [62]. Radiation-induced DSBs activate the p53 pathway, triggering ISC apoptosis [63], while simultaneous inhibition of Wnt/β-catenin signaling suppresses ISC proliferation and regeneration, effectively severing epithelial renewal [64].

At the structural level, radiation downregulates core tight junction proteins, including zonula occludens-1 (ZO-1) and claudin-3, through aberrant phosphorylation and proteasomal degradation, resulting in widened paracellular spaces [65, 66]. Adherens junctions are similarly disrupted, leading to disorganization of intercellular junctional complexes [16]. In addition, radiation perturbs epithelial polarity by impairing polarity-regulating proteins, causing apical–basal disarray and further compromising epithelial integrity.

The chemical barrier is primarily maintained by the mucus layer, which is enriched in mucins secreted by goblet cells. Radiation induces goblet cell apoptosis and rupture, markedly reducing mucin synthesis and thinning the mucus layer [67]. Concurrently, Paneth cell dysfunction diminishes antimicrobial peptide secretion, weakening the antimicrobial shield embedded within the mucus layer and increasing susceptibility to pathogenic invasion [16].

Radiation-associated microbial disturbances and reduced production of protective metabolites such as short-chain fatty acids (SCFAs) further destabilize the mucosal environment and limit the metabolic support required for epithelial maintenance and restitution [65].

**Fig. 2:**
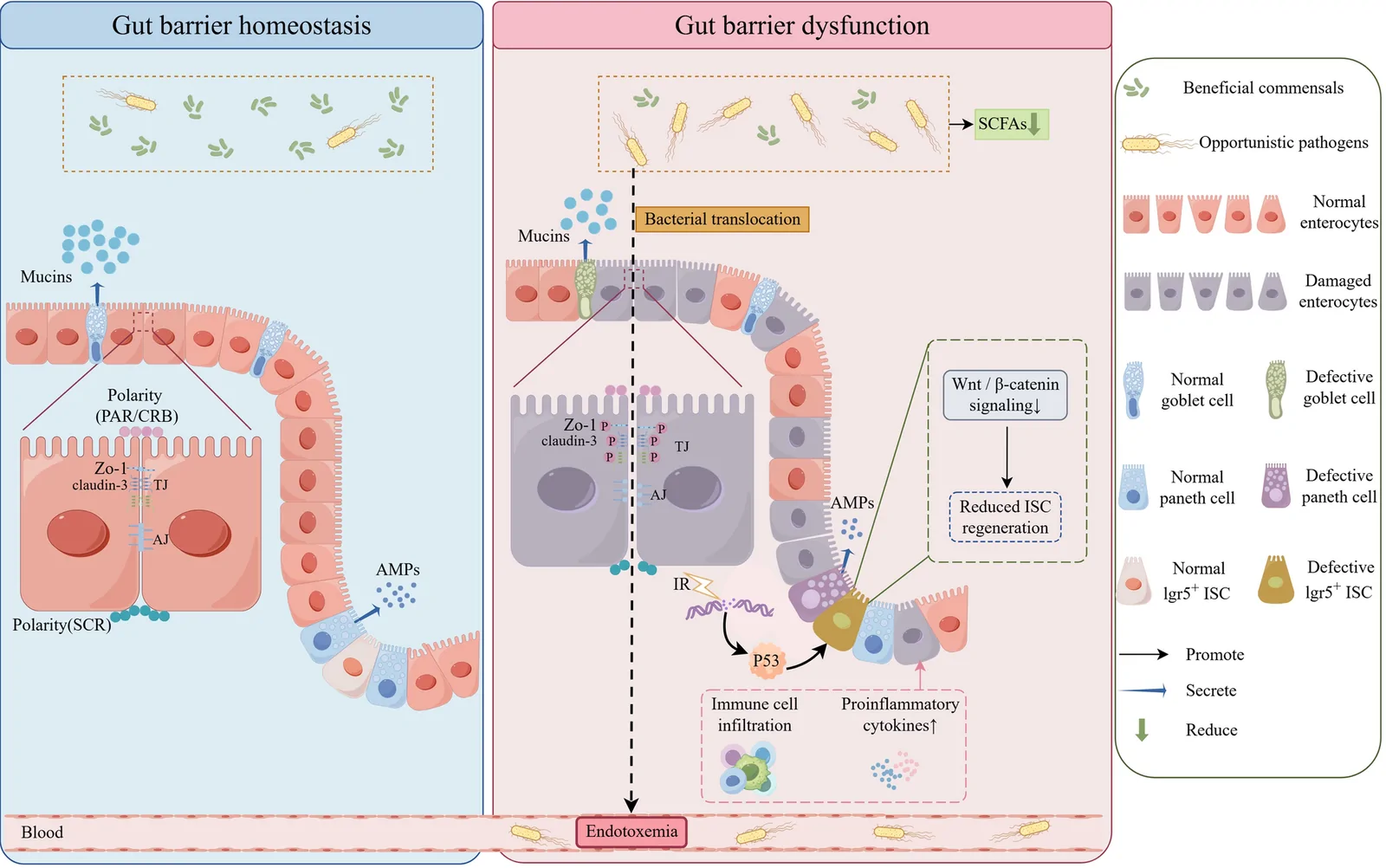
Radiation-induced intestinal epithelial barrier dysfunction. Radiation injures p53/LGR5-positive stem-cell compartments, suppresses Wnt/β-catenin-dependent regeneration, and disrupts junctions, polarity, goblet and Paneth cell function, and microbiota-derived short-chain fatty acids, leading to bacterial translocation, endotoxemia and inflammatory amplification. Black arrows indicate promotion; blue arrows secretion; ↑/↓ increases and decreases; and P phosphorylation. Key abbreviations: AJ, adherens junction; AMPs, antimicrobial peptides; CRB, Crumbs polarity complex; IR, ionizing radiation; ISC, intestinal stem cell; LGR5, leucine-rich repeat-containing G-protein-coupled receptor 5; PAR, partitioning-defective polarity complex; SCFAs, short-chain fatty acids; SCR, Scribble polarity complex; TJ, tight junction; ZO-1, zonula occludens-1

Immune cell infiltration and proinflammatory cytokine release further aggravate epithelial injury and barrier dysfunction [19, 68]. The combined breakdown of physical, chemical, microbial, and immune barriers facilitates bacterial translocation and endotoxemia, thereby amplifying local and systemic inflammation **(**Fig. 2**)**.

### Inflammatory and immune dysregulation

Radiation-damaged IECs release DAMPs such as HMGB1, ATP, and mitochondrial DNA [15, 19, 66, 69, 70]. These signals activate pattern recognition receptors on innate immune cells, particularly macrophages, inducing robust secretion of proinflammatory mediators including TNF-α, IL-6, and CXCL16, as well as activation of the NLRP3 inflammasome [71–74]. Damage-associated cytosolic DNA sensing through the cGAS-STING pathway may further amplify innate immune responses in radiation-induced intestinal injury [3].

Activation of caspase-1 promotes the maturation of IL-1β and IL-18, further amplifying inflammatory signaling [75–78]. TNF-α directly induces IEC apoptosis [79, 80], IL-6 sustains inflammation through the JAK/STAT3 pathway [81, 82], and CXCL16 promotes immune cell recruitment via CXCR6 signaling [83, 84]. Collectively, these mediators disrupt tight and adherens junctions, increasing epithelial permeability and facilitating translocation of gut-derived products such as lipopolysaccharide (LPS) [85]. LPS further activates the TLR4 pathway, reinforcing inflammatory cascades [16, 66, 86].

When acute inflammation fails to resolve, persistent immune dysregulation prolongs tissue injury and interferes with effective repair. Macrophage responses after irradiation are time-dependent: increased macrophage infiltration and proinflammatory M1-like features are commonly observed during active inflammatory injury, whereas the abundance and function of M2-like macrophages vary according to disease stage and tissue context [87]. Available evidence also indicates a shift in helper T-cell responses toward Th1 and Th17 predominance, accompanied by insufficient Treg-mediated control of inflammation [88–93]. DNA damage–associated signaling, including ATM activation, may further exacerbate aberrant T cell responses [94]. Chronic inflammation also promotes accumulation of myeloid-derived suppressor cells (MDSCs), which suppress T cell immunity but simultaneously impair tissue repair and regeneration [95–97]. Persistent microbiota dysbiosis further aggravates immune dysfunction by reducing SCFA availability, thereby weakening immune regulation and epithelial energy metabolism [98, 99]

**Fig. 3:**
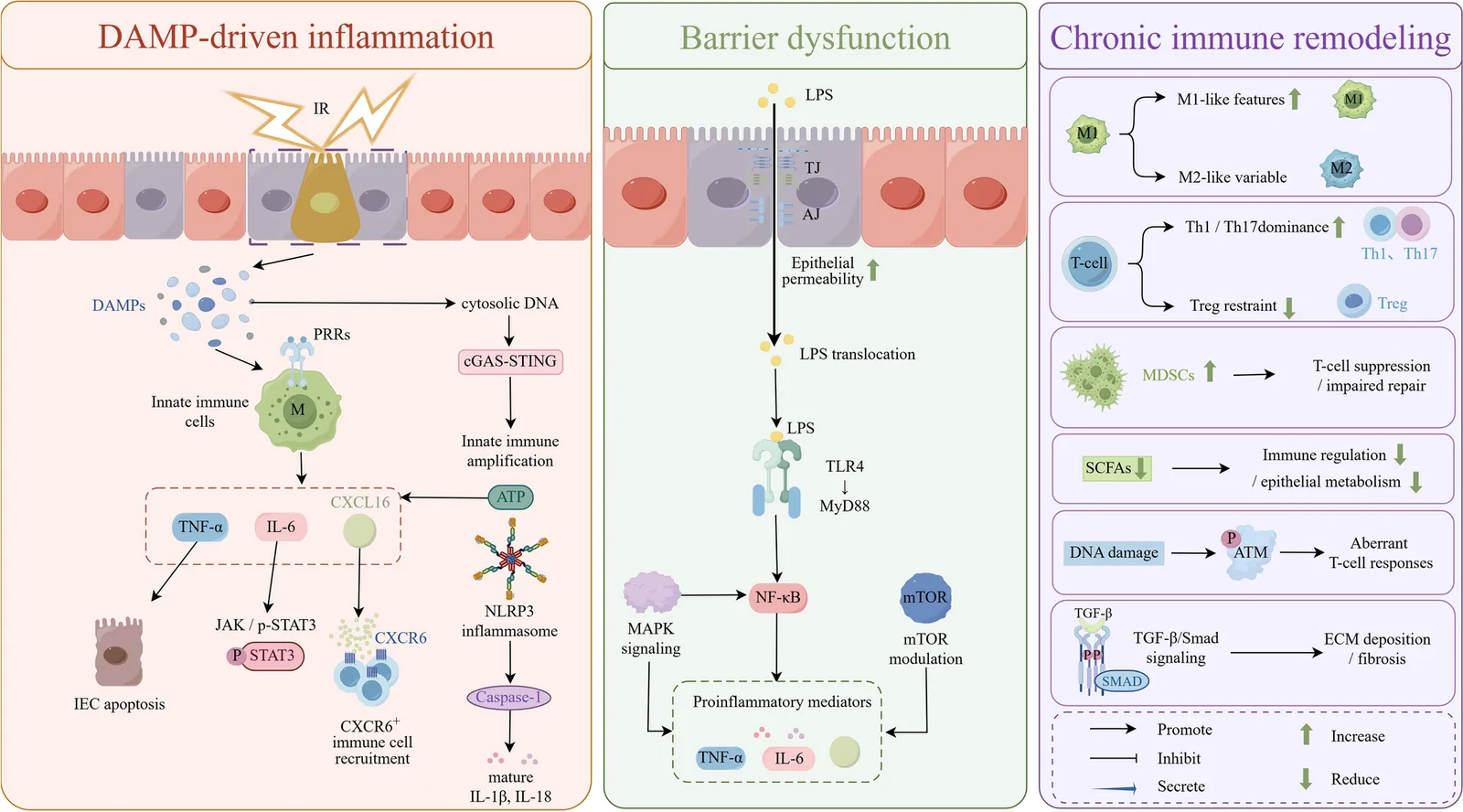
Inflammatory and immune dysregulation in radiation enteritis. Radiation-induced epithelial injury releases DAMPs and cytosolic DNA, activating PRR/cGAS-STING and inflammasome pathways; barrier failure and LPS translocation engage TLR4/MyD88/NF-κB, while persistent injury drives macrophage and T-cell imbalance, MDSC accumulation, SCFA loss and TGF-β/Smad-associated fibrosis. Arrows indicate activation or pathological progression; ↑/↓ indicate increase or reduction. Key abbreviations: DAMPs, damage-associated molecular patterns; PRRs, pattern-recognition receptors; cGAS-STING, cyclic GMP-AMP synthase-stimulator of interferon genes; LPS, lipopolysaccharide; TLR4, Toll-like receptor 4; MyD88, myeloid differentiation primary response 88; NF-κB, nuclear factor kappa B; NLRP3, NOD-like receptor family pyrin domain-containing 3; MDSCs, myeloid-derived suppressor cells; SCFAs, short-chain fatty acids; TGF-β, transforming growth factor beta; Th1/Th17, T helper 1/17 cells; Treg, regulatory T cell

At the signaling level, NF-κB functions as a central inflammatory hub activated through TLR4/MyD88 and intersecting with MAPK and inflammasome pathways [100–106]. The mTOR pathway modulates inflammatory mediator secretion [107, 108], whereas the TGF-β/Smad pathway links persistent immune dysregulation to tissue remodeling and fibrotic responses [109, 110] **(**Fig. 3**)**.

### Vascular injury and fibrotic remodeling

Radiation induces endothelial injury through direct DNA damage and ROS bursts, resulting in altered expression of adhesion molecules such as VE-cadherin, endothelial apoptosis, and loss of vascular barrier integrity [111]. High-dose radiation causes direct endothelial destruction, whereas low-dose radiation promotes endothelial–mesenchymal transition via ROS-mediated activation of NF-κB and MAPK pathways [112]. Endothelial dysfunction disrupts tissue perfusion, exacerbates inflammation, and promotes a prothrombotic state by reducing anticoagulant proteins such as thrombomodulin while increasing plasminogen activator inhibitor-1 (PAI-1), thereby favoring microthrombus formation [113, 114]. Radiation-induced phenotypic switching of vascular smooth muscle cells further contributes to vascular wall thickening and luminal narrowing [112, 113]. Although endothelial apoptosis is more pronounced in tumor vasculature, protective factors such as Robo4 partially preserve microvascular integrity in non-tumor tissues by regulating permeability [115, 116].

Vascular injury establishes ischemic, hypoxic, and inflammatory microenvironments that drive progressive fibrosis. Pericyte transdifferentiation mediated by the PDGF-BB/PDGFR-β axis represents a major source of myofibroblasts and ECM accumulation [117, 118]. Resident fibroblasts are activated via the TGF-β1/Smad2/3 pathway, with further amplification through the KLF6–ADAMTS1 axis [119–122]. Infiltrating immune cells, particularly M2 macrophages, secrete profibrotic mediators such as TGF-β and PDGF and promote myofibroblast differentiation through cGAS-STING signaling [123–126]. The CXCL16/CXCR6 axis serves as a critical molecular bridge linking vascular injury to fibrosis, acting synergistically with persistent oxidative stress and NF-κB/MAPK signaling [127, 128].

**Fig. 4:**
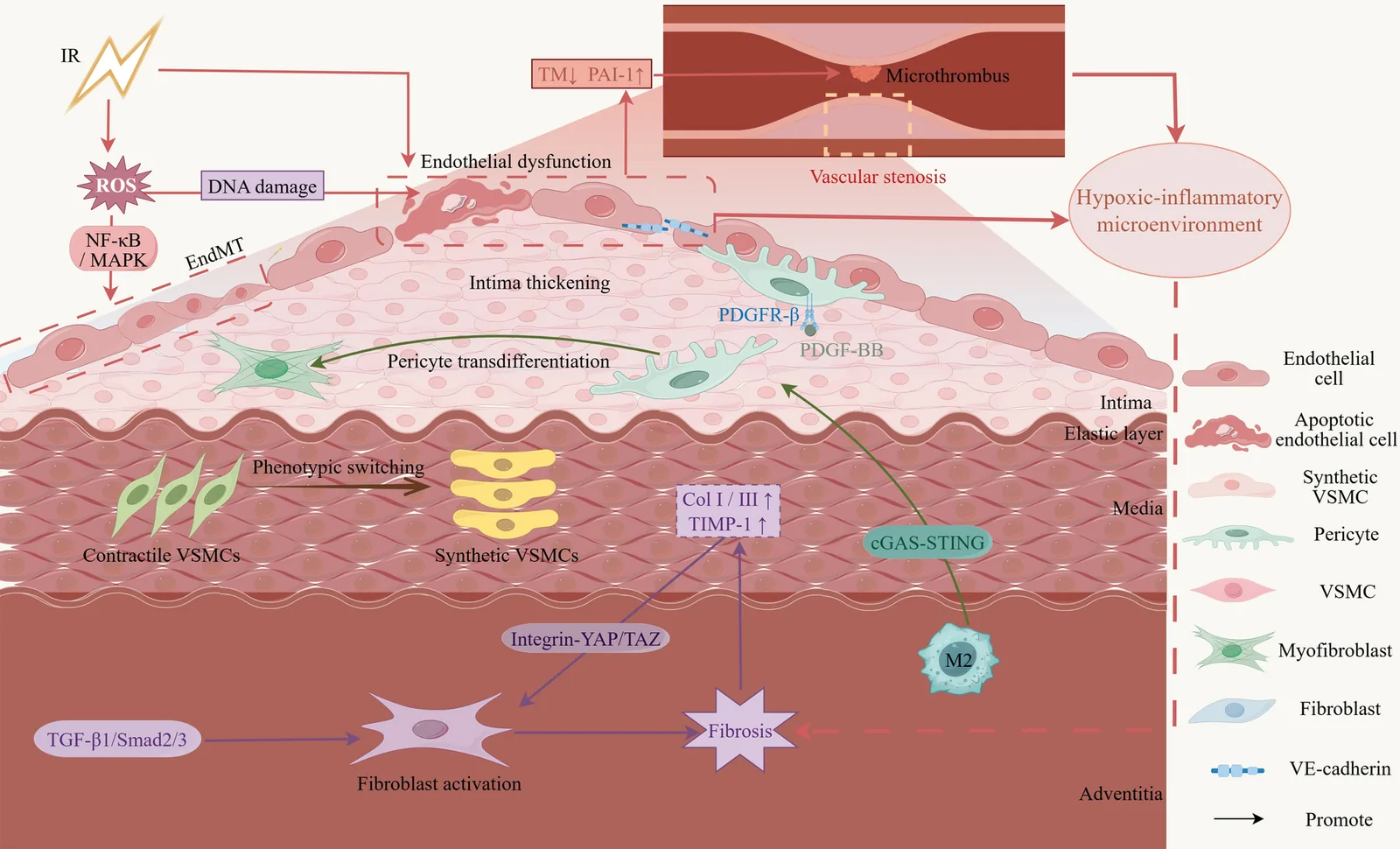
Radiation-induced vascular injury and fibrotic remodeling. IR-induced DNA damage and ROS activate NF-κB/MAPK signaling, causing endothelial dysfunction, EndMT, VSMC phenotypic switching, microthrombosis and vascular stenosis; hypoxia, immune signaling and fibroblast activation then promote extracellular-matrix deposition and fibrosis. Arrows indicate activation, promotion or phenotypic transition; ↑/↓ indicate increase or decrease. Key abbreviations: IR, ionizing radiation; ROS, reactive oxygen species; NF-κB, nuclear factor kappa B; MAPK, mitogen-activated protein kinase; EndMT, endothelial-to-mesenchymal transition; VSMCs, vascular smooth muscle cells; cGAS-STING, cyclic GMP-AMP synthase-stimulator of interferon genes; ECM, extracellular matrix; Col I/III, collagens I and III; PDGFR-β, platelet-derived growth factor receptor beta; TGF-β1, transforming growth factor beta 1; Smad2/3, SMAD family members 2 and 3; TIMP-1, tissue inhibitor of metalloproteinases-1

Fibrosis is further reinforced by dysregulated ECM turnover, characterized by increased synthesis of type I and III collagens [129–131] and reduced degradation due to upregulation of tissue inhibitors of metalloproteinases such as TIMP-1 [128, 132]. Increased ECM stiffness activates mechanotransduction pathways involving integrins and YAP/TAZ, establishing a positive feedback loop that perpetuates fibrotic remodeling [133–135] **(**Fig. 4**)**.

### Gut microbiota dysbiosis

Radiation profoundly alters gut microbial composition by inducing DNA damage and oxidative stress, leading to reduced diversity and depletion of dominant phyla such as Firmicutes and Bacteroidetes, alongside expansion of opportunistic pathogens including Proteobacteria and Fusobacteria [136–138]. This dysbiosis exhibits temporal persistence and may last for several months following radiotherapy [138]. Overgrowth of mucin-degrading bacteria such as *Akkermansia muciniphila* accelerates mucus layer thinning during radiation injury [18, 139]. Following barrier disruption, increased exposure of epithelial and immune cells to microbial products further aggravates mucosal inflammation [140, 141].

Paneth cell dysfunction further destabilizes microbial homeostasis by reducing secretion of α-defensins such as HD-5 and HD-6 [142, 143]. Impaired Paneth cell–mediated Wnt signaling disrupts goblet cell differentiation and mucin production, compounding barrier failure [144]. The combined loss of antimicrobial peptides and mucus promotes sustained dysbiosis and weakens intestinal immune defense [145–147].

Radiation-induced microbial dysbiosis also disrupts metabolic homeostasis. Depletion of beneficial metabolites, particularly butyrate, reduces metabolic support for epithelial survival and weakens immune regulation [8, 107, 148–151]. Meanwhile, increased exposure to LPS and other microbial products reinforces inflammatory signaling and may increase epithelial radiosensitivity [138, 152]. These functional changes can further delay epithelial restitution and prolong mucosal inflammation.

**Fig. 5:**
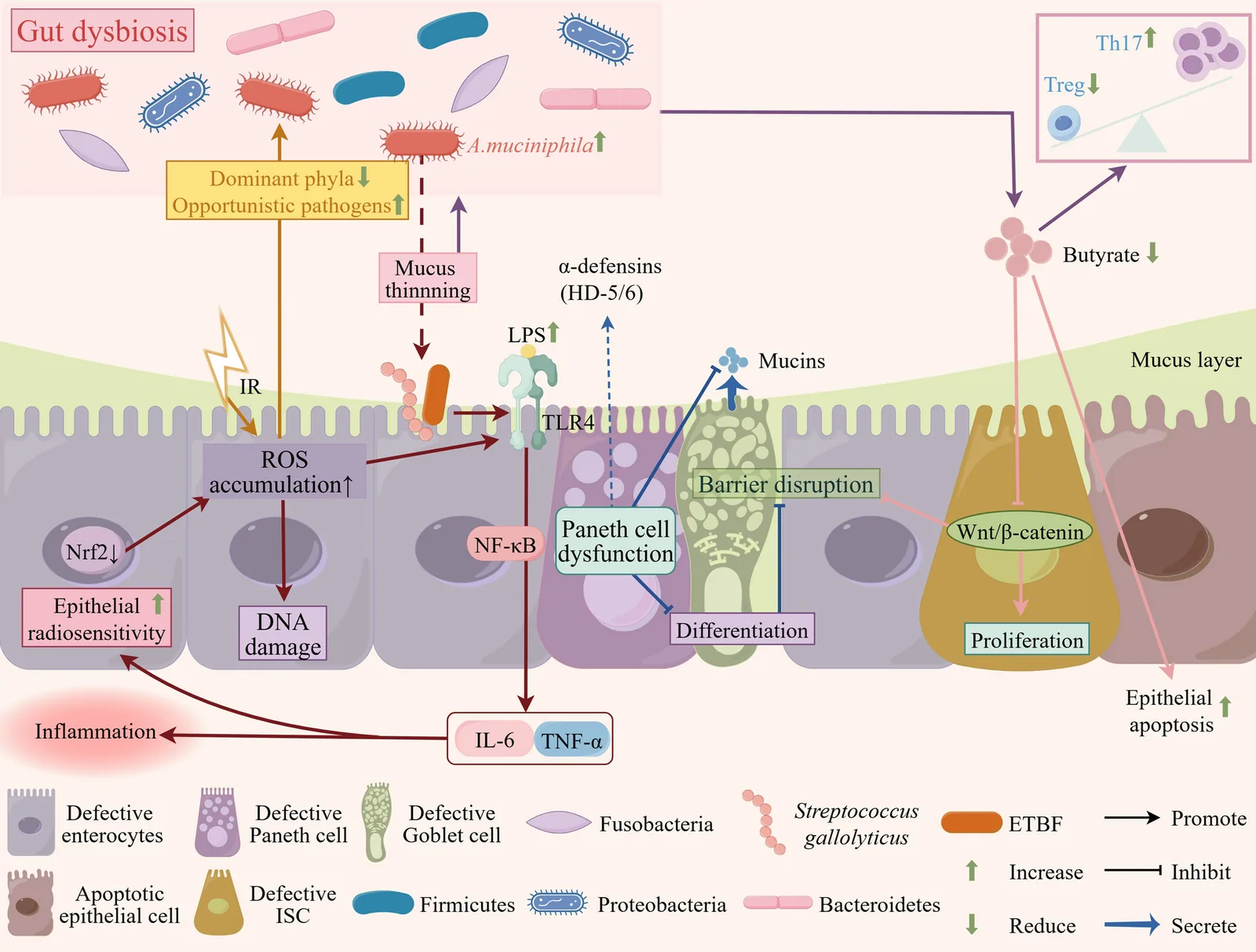
Radiation-induced gut microbiota dysbiosis. Radiation shifts microbial composition and weakens mucus and Paneth-cell defenses, causing barrier disruption and LPS-TLR4/NF-κB inflammation; loss of butyrate impairs Wnt/β-catenin-dependent ISC proliferation and Treg/Th17 balance, while reduced Nrf2 increases oxidative and epithelial injury. Black arrows indicate promotion; blunt-ended lines inhibition; blue arrows secretion; and ↑/↓ increase or decrease. Key abbreviations: *A. muciniphila*, *Akkermansia muciniphila*; ETBF, enterotoxigenic *Bacteroides fragilis*; IR, ionizing radiation; ISC, intestinal stem cell; LPS, lipopolysaccharide; NF-κB, nuclear factor kappa B; Nrf2, nuclear factor erythroid 2-related factor 2; ROS, reactive oxygen species; TLR4, Toll-like receptor 4; Treg, regulatory T cell; Th17, T helper 17 cell

Radiation also alters the intestinal virome, reducing protective bacteriophages and facilitating pathogen expansion, which further aggravates mucosal injury and inflammation [136, 153, 154]. Although the causal contribution of virome alterations remains less well established, gut microbiota dysbiosis overall acts as an amplifier linking defective barrier repair with persistent immune activation **(**Fig. 5**)**.

### Integrated progression from acute injury to chronic remodeling

Rather than operating as independent cascades, the mechanisms described above form an interconnected and time-dependent process that contributes to RE progression. In the early phase after irradiation, direct DNA damage and excessive ROS production injure crypt epithelial cells and ISCs and perturb the vascular endothelium, initiating cell loss, barrier disruption, and innate immune activation. The subsequent course partly depends on the capacity for epithelial regeneration, as Lgr5-positive ISCs are required for robust crypt regeneration following radiation injury [155]. Redox regulation and damage-associated innate immune sensing also intersect during this phase. In experimental radiation-induced intestinal injury, Nrf2 deficiency aggravates tissue damage and enhances cGAS-STING signaling, indicating functional crosstalk between redox regulation and innate immune activation [3]. Thus, the initial radiation insult may rapidly extend beyond direct cytotoxicity through reciprocal amplification of oxidative stress, cellular injury, and inflammation.

As epithelial and junctional integrity deteriorates, barrier dysfunction increasingly interacts with microbial and inflammatory changes. In an acute total-abdominal irradiation model, gut dysbiosis and depletion of butyrate-producing bacteria are accompanied by increased fecal LPS levels and activation of TLR4/MyD88/NF-κB signaling [156]. These changes are consistent with a reinforcing interaction in which barrier disruption increases exposure to microbe-derived inflammatory stimuli, while persistent inflammation further impairs epithelial restitution and barrier repair. Failure to restore epithelial and microbial homeostasis may therefore prolong tissue injury beyond the initial radiation response.

With persistent injury, vascular dysfunction and maladaptive tissue repair become increasingly prominent. Experimental sensitization of intestinal endothelial cells to radiation did not markedly exacerbate the acute gastrointestinal syndrome but increased susceptibility to delayed radiation enteropathy, accompanied by microvascular loss, increased vascular permeability, and tissue hypoxia [157]. These findings indicate that vascular injury has an important role in the development of delayed intestinal damage. Importantly, chronic RE should not be viewed simply as a linear extension of acute mucosal injury. Experimental evidence indicates that chronic injury may reflect both consequential effects of severe acute mucosal damage and primary injury to late-responding tissue compartments [158]. Persistent hypoxia, unresolved inflammation, and defective epithelial regeneration may subsequently favor fibroblast activation and progressive ECM accumulation. Consistent with this transition, stage-dependent shifts in TGF-β1 expression and cellular sources have been observed between early and chronic radiation enteropathy, supporting its involvement in chronic fibrotic remodeling [159].

**Fig. 6:**
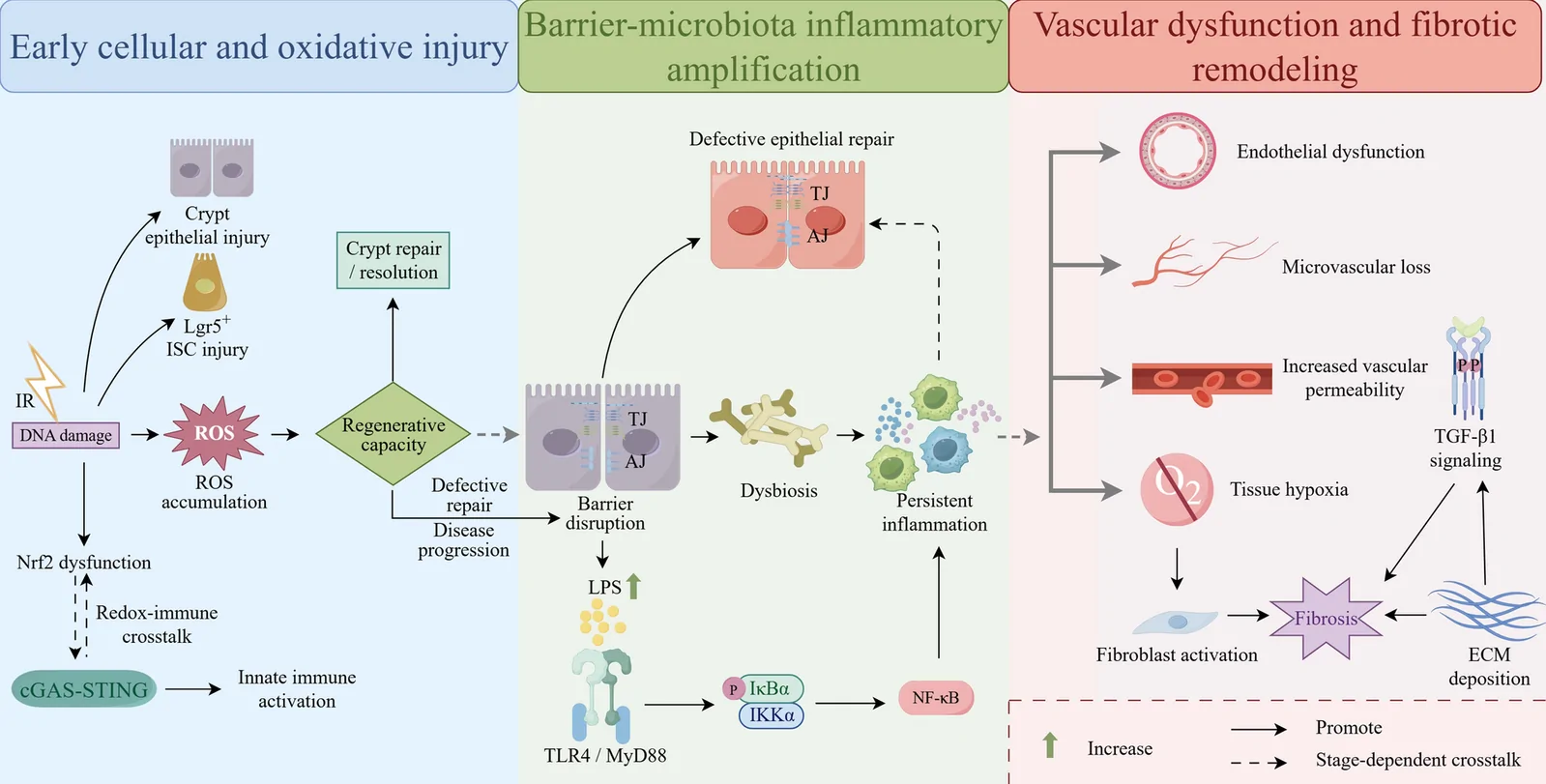
Integrated progression from acute radiation injury to chronic intestinal remodeling. Early DNA and ROS injury affects crypt epithelium and LGR5-positive intestinal stem cells; recovery depends on regenerative capacity, whereas persistent barrier, microbial, immune and vascular injury promotes hypoxia, fibroblast activation and fibrosis. Solid arrows indicate promotion or pathological progression; dashed arrows indicate stage-dependent crosstalk; and ↑ indicates increased expression or activity. Key abbreviations: IR, ionizing radiation; ROS, reactive oxygen species; ISC, intestinal stem cell; LGR5, leucine-rich repeat-containing G-protein-coupled receptor 5; AJ/TJ, adherens/tight junctions; cGAS-STING, cyclic GMP-AMP synthase-stimulator of interferon genes; TLR4/MyD88, Toll-like receptor 4/myeloid differentiation primary response 88; NF-κB, nuclear factor kappa B; Nrf2, nuclear factor erythroid 2-related factor 2; TGF-β1, transforming growth factor beta 1; ECM, extracellular matrix

Collectively, RE can be viewed as a pathological continuum comprising overlapping phases of initial cellular and oxidative injury, inflammatory and microbial amplification, incomplete epithelial repair with progressive vascular dysfunction, and chronic fibrotic remodeling. Whether injured intestinal tissue recovers or progresses toward persistent structural damage likely depends on the balance between regenerative capacity and sustained oxidative, immune, microbial, and vascular injury. Within this framework, redox imbalance, inflammatory responses, microbiota dysbiosis, epithelial regeneration, and vascular dysfunction interact across different stages of disease progression rather than acting as isolated pathogenic pathways, providing an integrated basis for understanding the transition from acute radiation injury to chronic RE **(**Fig. 6**)**.

## TCM formulas for RE: clinical and preclinical evidence

From a TCM perspective, radiation enteritis (RE) is commonly interpreted within the categories of diarrhea, dysentery, hematochezia, and related intestinal disorders. Traditional interpretations generally emphasize the combined involvement of radiation-induced heat toxin and blood stasis, impaired spleen function, and depletion of yin fluids. These interpretations inform therapeutic principles such as clearing heat and resolving toxicity, activating blood circulation, strengthening the spleen and replenishing qi, nourishing yin, and harmonizing intestinal function. From a biomedical perspective, however, multi-herb formulas should not be evaluated by assigning each traditional therapeutic principle to an individual molecular target. A more relevant question is whether a standardized formula shows reproducible effects across epithelial injury, inflammation, defective repair, microbial disturbance, vascular dysfunction, and tissue remodeling.

The preclinical evidence summarized in **Tables S1 and S2** is unevenly distributed across disease stages and intestinal compartments. Representative formulas are discussed here to illustrate differences in disease stage, intestinal site, and administration route, whereas detailed study characteristics and findings are provided in Tables 1**, S1, and S2**. Changrui enema has been evaluated in acute rectal injury, Liangxue Guyuan Yishen Decoction (LGYD) primarily in acute small-intestinal injury following whole-body irradiation, and Shaoyao Decoction in acute colonic injury [160–162]. Baitouweng Decoction has also been examined in a persistent or subacute rectal-injury model characterized by collagen deposition and TGF-β/Smad-associated remodeling, representing one of the few formula studies addressing persistent remodeling rather than predominantly acute injury. Dedicated late or chronic formula models nevertheless remain scarce [163]. Oral administration predominates in small-intestinal or multisite models, whereas retention enemas are used more frequently for localized rectal injury. These differences limit direct comparison because observed efficacy may depend on local drug exposure, irradiation field, treatment timing, and the affected intestinal compartment. The formulas highlighted here should therefore be regarded as illustrative examples from different experimental settings rather than evidence of stage-specific superiority or interchangeability.

**Table 1:** Clinical evidence for TCM formulas in RE

Intervention	Stage/site; population	Design; n; comparator	Dose; route; duration	Endpoints	Core findings	Safety	Study-specific limitations	References
Anchang Formula retention enema	ARE; rectum/radiation proctitis; therapeutic; Adults with acute radiation enteritis after pelvic radiotherapy for gynecologic, rectal, prostate or bladder cancer	Single-center randomized controlled study; n = 93 (48/45); random-number table; Anchang Formula vs saline + dexamethasone + montmorillonite retention enema	Anchang Formula decocted/concentrated to ~ 100 mL as retention enema b.i.d.; for 14 days; comparator: 100 mL saline + dexamethasone 5 mg + montmorillonite (2 sachets), same schedule	Composite clinical response; endoscopic response; TCM symptom scores	Higher composite clinical response (93.75% vs 68.89%) and endoscopic response (89.58% vs 62.22%) (both P < 0.05); greater symptom improvement; IL-2/IFN-γ decreased and IL-10 increased vs control	Safety assessment: NR	Single-center study; blinding/allocation concealment not reported; NR	[169]
Anchang Zufang retention enema	ARE; rectum/radiation proctitis; therapeutic; 68 patients with damp-heat type acute radiation enteritis after pelvic radiotherapy	Single-center uncontrolled before–after study; n = 68; no comparator or randomization	Herbal decoction concentrated to ~ 100 mL as retention enema b.i.d.; for 14 days; symptom-based herb additions permitted	Vienna rectoscopy score; QLQ-C30 quality of life; T-cell subsets (CD3 +, CD4 +, CD8 +)	Post-treatment rectoscopy score decreased; overall health, physical, emotional and social QLQ-C30 domains improved; CD3 +, CD4 + and CD8 +	Safety assessment: NR	Uncontrolled before–after design; no comparator/blinding; short follow-up and no systematic safety assessment	[170]
Modified Baitouweng Decoction retention enema	CRE; rectum/radiation proctitis; unspecified; Chronic radiation enteritis after abdominal/pelvic radiotherapy	Single-center controlled study; n = 59 (32/27); allocation described as random by admission order, method unclear; comparator: the same nutritional/supportive and symptomatic care without the herbal enema	Modified Baitouweng Decoction concentrated to 100–120 mL; retention enema once nightly q.n.; for 15 days	Composite clinical/endoscopic response; hemorheology; serum immunoglobulins	Overall response was 90.7% vs 70.3% (P < 0.01); treatment also produced greater reductions in blood-viscosity indices and IgG/IgA-related measures	Safety assessment: NR	Small single-center sample; allocation/randomization method and blinding unclear; radiotherapy parameters and long-term outcomes NR	[171]
Bazhen Decoction (oral) + Kangfuxin Liquid retention enema	CRE; rectum/radiation proctitis; therapeutic; Chronic radiation proctitis diagnosed > 6 months after pelvic radiotherapy for pelvic malignancies	Single-center randomized controlled study; n = 64 randomized (32/32), 61 analyzed (31/30); random-number table; Bazhen Decoction + Kangfuxin vs Kangfuxin alone	Bazhen Decoction: 1 daily dose decocted to ~ 200 mL and divided morning/evening for 2 weeks; Kangfuxin Liquid 120 mL retention enema, retained ≥ 2 h, for 2 weeks; enema frequency NR	Composite clinical response; abdominal pain, loose stool, hematochezia and anal-tenesmus symptom scores; TCM secondary-symptom scores	Composite response was 87.10% vs 80.00% (reported P < 0.05); post-treatment main- and secondary-symptom scores were lower with add-on Bazhen Decoction (P < 0.05)	One control patient discontinued because of an allergic reaction; systematic comparative adverse-event assessment was not reported	Small single-center study with attrition; blinding/allocation concealment not reported; NR	[172]
Dahuang Fengfang Yin retention enema	ARE; rectum/radiation proctitis; therapeutic; Radiation proctitis after pelvic radiotherapy for gynecologic and other malignancies	Single-center uncontrolled case series; n = 31; no comparator/randomization/blinding	Rhubarb 20 g + Vespae Nidus 100 g + *Glycyrrhiza* 100 g + *Bletilla* 30 g; decocted to 200 mL; retention enema q.d., delivered in three portions per treatment	Clinical symptoms and endoscopic mucosal response; overall clinical response	After 3 courses, 24/31 patients improved and 7/31 were ineffective; overall response 77.4%	Safety assessment: NR	Small uncontrolled case series; no comparator/randomization/blinding; mixed disease duration without phase stratification	[173]
Modified Diyu Decoction retention enema	ARE; rectum/radiation proctitis; therapeutic; Hospitalized patients with acute radiation proctitis developing during radiotherapy for pelvic/abdominal/retroperitoneal malignancies	Single-center controlled study; 64 randomized (33/31), 62 analyzed (32/30); randomization reported, method/blinding/allocation concealment: NR; herbal enema vs Smecta + dexamethasone enema	Modified Diyu decoction concentrated to 100–120 mL; retention enema once nightly q.n.; at 38–40°C for 10 days	Composite clinical response; rectal symptoms/bleeding and endoscopic mucosal findings; adverse reactions	Overall response did not differ significantly NS (93.75% vs 90.0%, P > 0.05); cure + marked-response proportion was higher with the herbal enema (84.4% vs 60.0%, P < 0.05)	No obvious adverse reactions were reported in either group	Small single-center study; randomization method/blinding not reported; NR	[174]
Modified Erqi Decoction (oral + retention enema)	CRE; rectum/radiation proctitis; therapeutic; Radiation enteritis/proctitis after radiotherapy for abdominal, pelvic, or retroperitoneal malignancies	Single-center controlled study; n = 127 (92/35); randomized allocation reported, method/blinding/allocation concealment: NR; Modified Erqi Decoction vs Smecta-based oral/enema therapy	Oral decoction 400 mL/day in 2 divided doses + 200 mL retention enema once daily; 1-week course × 4 (4 weeks)	Composite clinical response; TCM symptom scores; persistent symptoms/rectal perforation at 1–3 years in a followed responder subset	Overall response 93.5% vs 77.1% (P < 0.05); symptom scores improved more rapidly; among followed responders (23 vs 16), persistent symptoms at 1–3 years were less frequent (47.8% vs 68.8%)	Safety assessment: NR	Highly imbalanced groups; randomization method/blinding not reported; NR	[175]
Fuzhen Zhixie Decoction (oral)	ARE; rectum/radiation proctitis; therapeutic; Acute/subacute radiation enteritis after abdominal/pelvic radiotherapy for cervical, uterine, rectal, or bladder cancer	Single-center randomized controlled study; n = 128 (68/60); random-number table; Fuzhen Zhixie Decoction vs ofloxacin + montmorillonite	Hospital-prepared decoction, 1 dose/day p.o. in 2 divided warm doses for 14 days; symptom-based modifications permitted	Composite clinical response; colonoscopic mucosal response; TCM symptom scores	Overall response 95.59% vs 68.33% and colonoscopic response 94.12% vs 80.00% (both P < 0.05); symptom scores decreased and KPS improved more than with control treatment	No treatment-related blood, urine, liver/renal, or ECG abnormalities; mild nausea in 2/68 vs nausea/vomiting in 8/60, resolving spontaneously or with symptomatic treatment	Single-center study; blinding/allocation concealment not reported; NR	[168]
Modified Gegen Qinlian decoction (GQD)	CRE; rectum/radiation proctitis; therapeutic. 51-year-old woman with cervical cancer	Single-patient case report (n = 1); no comparator	150 mL retention enema once nightly for 1 month, followed by modified oral GQD 150 mL three times daily for 5 months	Diarrhea frequency; tenesmus/abdominal pain; colonoscopic ulcer/telangiectasia	Diarrhea decreased from 5–6 to 1–2 times/day; rectal ulcer healed after 1 month; telangiectasia/intestinal-wall abnormalities markedly improved after 6 months	No adverse reactions were reported during treatment, including liver or renal function injury	Single case with no comparator; enema and oral formulations differed and were used sequentially, limiting attribution; controlled long-term efficacy/safety data unavailable	[176]
Gegen Qinlian Decoction (oral)	ARE; rectum/radiation proctitis; therapeutic; Locally advanced rectal cancer with intestinal damp-heat-pattern acute radiation proctitis developing during radiotherapy	Single-center randomized controlled study; n = 30 (15/15); randomization/blinding: NR; comparator: dioctahedral smectite	Gegen Qinlian Decoction p.o., 1 dose/day for 14 days; comparator dioctahedral smectite p.o. three times daily	Composite short-term clinical response and associated symptom improvement	Overall response was 86.67% vs 53.33% (P < 0.05)	Safety assessment: NR	Very small single-center sample; randomization/blinding details NR; short 14-day assessment	[177]
Modified Gegen Qinlian Decoction (oral)	ARE; unspecified; therapeutic; Acute radiation enteritis within 90 days after abdominal/pelvic/retroperitoneal radiotherapy	Single-center randomized controlled study; n = 70 (35/35); random-number table; Modified Gegen Qinlian Decoction vs montmorillonite	1 decoction/day, prepared twice and taken warm morning/evening; 14 days/course; continuation reported	Symptom scores; composite clinical response; WBC/PLT	Response 94.29% vs 77.14% (P < 0.05); symptom scores were lower, and WBC/PLT plus IgG/IgM/IgA were higher than comparator after treatment (P < 0.05)	No obvious changes in routine blood/urine/stool tests, ECG, or liver/renal function were reported	Small single-center study; blinding/allocation concealment not reported; NR	[178]
Self-made herbal decoction (Gong'ai Changqing Formula) retention enema	Mixed; rectum/radiation proctitis; therapeutic; Cervical-cancer radiation proctitis after standardized radiotherapy	Single-center randomized controlled study using a random-number table; n = 120 (60/60); comparator: gentamicin + dexamethasone + lidocaine + saline retention enema; allocation concealment and blinding NR	One daily prescription decocted into two ~ 100-mL bags; retention enema twice daily, b.i.d., retained ≥ 1 h; 10 days/course × 2 courses	Symptom scores; EORTC/RTOG rectal radiation-injury grade; KPS	After 2 courses, symptom scores were lower, radiation-injury grades improved more, and KPS was higher (85.98 ± 7.02 vs 65.01 ± 3.22) than control (reported P < 0.05)	No adverse reactions were observed in either group during treatment; blood, liver, renal and coagulation monitoring was specified	Single-center study; allocation concealment/blinding NR; acute/chronic phase not stratified and RT regimen NR	[179]
Huai Jiang Fang (oral administration + retention enema)	Mixed; rectum/radiation proctitis; unspecified; Endoscopically confirmed radiation enteritis after abdominal/pelvic radiotherapy	Single-center randomized controlled study; n = 84 (42/42); random-number table; Huai Jiang Fang vs dexamethasone + saline retention enema	Egg-yolk oil 10 g + carbonized hair 5 g + carbonized *Sophora flower* 5 g/d; decocted to 400 mL: 200 mL p.o	Clinical/endoscopic response; symptom scores; CRP and oxidative-stress markers	Overall response 95.24% vs 76.19% (P = 0.013); lower post-treatment symptom scores, CRP/NO/MDA and higher SOD vs control (P < 0.05)	Safety assessment: NR	Single-center; blinding/allocation concealment not reported; NR	[180]
Huazhuo Guanchang Formula retention enema	ARE; rectum/radiation proctitis; unspecified; Pathologically confirmed rectal, cervical, or endometrial cancer with pelvic-radiotherapy–associated acute radiation proctitis and heat-toxin injury–collateral TCM pattern	Single-center randomized controlled study; n = 80 (40/40); random-number table; Huazhuo Guanchang Formula vs montmorillonite + dexamethasone enema	One daily herbal dose decocted twice and combined to 150 mL for retention enema; 4 weeks; exact enema frequency beyond the reported daily dose NR	TCM symptom scores/composite response; acute-radiation-proctitis grade; Karnofsky Performance Status	Lower post-treatment symptom scores; composite response 90.0% vs 72.5%; acute-radiation-proctitis grade and KPS favored the herbal group (all reported P < 0.05)	No adverse reactions were reported; systematic safety-assessment methods were not described	Single-center; blinding/allocation concealment not reported; NR	[181]
Leibai Enema	Mixed; rectum/radiation proctitis; therapeutic; Colonoscopy-confirmed hemorrhagic radiation proctitis after pelvic irradiation	Single-center randomized controlled study; n = 30 (15/15); randomization/blinding: NR; comparator: mesalazine retention enema	Herbal decoction prepared by 3 decoctions and concentrated to 120 mL; ~ 40 mL retention enema q.d.; at 36–40°C, retained ≥ 2 h, for 14 days	Composite clinical response based on abdominal pain/tenesmus, rectal bleeding, and fecal occult blood	Overall response 73.3% vs 33.3% (P < 0.05)	Safety assessment: NR	Very small single-center sample; randomization/blinding methods NR; injury phase and actual radiotherapy regimen incompletely characterized	[182]
Modified Liangxue Jiedu Fang retention enema	ARE; rectum/radiation proctitis; therapeutic; Cervical-cancer patients with acute radiation proctitis after radiotherapy	Single-center controlled study; n = 80 (40/40); random-number-table allocation reported; modified herbal enema + conventional Western therapy vs conventional Western therapy	*Bletilla* 30 g, *Cimicifuga* 20 g, *charred Platycladus* 10 g, *Angelica* 10 g and *Phellodendron* 10 g decocted to 50 mL; 50-mL retention enema b.i.d.; for 2 weeks	Composite clinical response incorporating symptoms/stool occult blood/endoscopy; SF-36 quality of life; reported common complications	Composite response was 90.0% vs 66.7% (P < 0.05); selected SF-36 domains (role limitation, bodily pain, general health, emotional role and mental health) favored the intervention (P < 0.05)	Treatment-emergent adverse events were not systematically characterized; reported common-complication incidence was 20.0% vs 25.0%	Small single-center study; blinding/allocation concealment not reported; design wording internally inconsistent; safety reporting and phytochemical standardization limited	[183]
Pingkuisan powder retention enema	ARE; rectum/radiation proctitis; therapeutic; Acute radiation proctitis after postoperative pelvic 60Co radiotherapy for cervical, endometrial, ovarian, prostate, or bladder cancer	Single-center controlled study reported as randomized; n = 60 (30/30); Pingkuisan vs gentamicin + lidocaine + dexamethasone retention enema; randomization method/blinding/allocation concealment: NR	Pingkuisan 24 g dispersed in 200 mL boiled water, cooled to ~ 38 °C and filtered; retention enema twice daily, b.i.d., retained ≥ 30 min, for 14 days	Composite clinical response; symptom scores; rectoscopic mucosal-injury grade	Overall response was 90.0% vs 73.3% (P < 0.05); key symptom scores and rectoscopic mucosal-injury grade improved more than with the comparator	Safety assessment: NR	Small single-center study; randomization method/blinding/allocation concealment not reported; NR	[184]
RP-II Chinese herbal rectal preparation	ARE; rectum/radiation proctitis; prophylactic; Women with gynecologic malignancies undergoing high-dose-rate intracavitary radiotherapy, with external-beam irradiation when indicated	Controlled comparative study; n = 600 (500/100); RP-II vs distilled-water rectal capsule/control; allocation/randomization/blinding not reported	One 15-mL RP-II capsule inserted rectally immediately before each intracavitary irradiation; from the next day, one capsule after bowel evacuation/enema 6 d/wk until completion of intracavitary radiotherapy; 4–14 administrations (mean 9)	Incidence and grade of acute/near-term radiation proctitis during radiotherapy	Radiation proctitis occurred in 1.2% vs 37.0% (P < 0.001); grade 0/I/II/III: 98.8%/1.0%/0.2%/0% vs 63%/34%/3%/0%	Safety assessment: NR	Allocation/randomization/blinding not reported; NR; markedly imbalanced groups and only broadly comparable radiotherapy regimens	[185]
Sechang Tang	Mixed; rectum/radiation proctitis; unspecified; Patients with radiation enteritis after cancer radiotherapy (cervical, rectal, bladder, or renal cancer)	Single-center randomized controlled study; n = 56 (28/28); randomization/blinding: NR; comparator: dexamethasone 10 mg + normal saline 100 mL retention enema	Oral decoction, 300 mL/day in 2 divided doses before meals for 15 days	Composite clinical response based on symptom improvement and colonoscopic mucosal recovery	Total response was 89.28% vs 75.00% (P < 0.05)	No drug-related adverse reactions were reported during treatment in either group	Small single-center sample; randomization/blinding methods not reported; NR	[186]
Modified Shaofuzhuyu Decoction retention enema	CRE; rectum/radiation proctitis; therapeutic; Qi-deficiency and blood-stasis–type chronic radiation proctitis after pelvic radiotherapy	Single-center randomized single-blind controlled study; n = 30 (15/15); modified Shaofuzhuyu Decoction vs dexamethasone + gentamicin retention enema; sequence generation/allocation concealment and blinded party NR	Modified decoction 100 mL by retention enema q.d., retained ≥ 30 min; 1-month course for 2 courses	Composite clinical response; TCM symptom scores; serum TNF-α and IL-6	Clinical response was 93.3% vs 60.0% (P < 0.05); post-treatment symptom scores and TNF-α/IL-6 were lower; 6-month	No adverse reactions were reported during treatment and follow-up	Small single-center study; randomization/allocation-concealment and blinding details incomplete; cytokine changes are associative rather than mechanistic	[167]
Modified Shenling Baizhu San	Unspecified; prophylactic; Cervical or endometrial cancer	Single-center randomized controlled study; n = 56 (30/26); randomization/blinding: NR; comparator: radiotherapy alone	Oral decoction 300 mL/day in 2 divided doses, from RT day 1 through completion (35–38 doses)	Bowel frequency, mucus stool and lower-abdominal pain during RT; colonoscopy-confirmed radiation-enteritis incidence/grade at 1 year	Frequent stools, mucus stool and lower-abdominal pain were less common during RT; at 1 year, radiation enteritis occurred in 43.3% vs 96.2% (P < 0.01 in the main text)	No herb-related adverse reactions were observed; systematic laboratory safety assessment NR	Small single-center study; randomization/blinding methods NR; no placebo	[187]
Shihui San retention enema	ARE; unspecified; therapeutic; Acute radiation enteritis within 90 days after abdominal/pelvic radiotherapy for malignancy	Single-center controlled study reported as randomized; n = 60 (30/30); randomization method/blinding/allocation concealment: NR; Shihui San enema-based regimen vs Western symptomatic treatment	50–100 mL retention enema twice daily, continued b.i.d., until 48 h after diarrhea resolution; efficacy assessed at 2 weeks; symptom-specific Western rectal adjuncts/rescue therapy were permitted	Major symptom scores (diarrhea, stool consistency, hematochezia, abdominal pain, tenesmus); composite clinical response; adverse reactions	Major symptom improvement favored Shihui San; overall response 80.0% vs 53.3% (P < 0.05)	No adverse reactions were reported in either group; routine blood/stool and liver/renal tests were monitored, but detailed safety results were not provided	Small single-center study; randomization method/blinding/allocation concealment not reported; NR	[188]
Self-made SI Cao Decoction (SSCD) retention enema	Unspecified; rectum/radiation proctitis; therapeutic; Radiation enteritis after pelvic/rectal radiotherapy for colorectal, cervical, prostate, or renal cancer	Single-center randomized controlled study; n = 78 (39/39); random-number table; SSCD retention enema vs montmorillonite + dexamethasone retention enema	Herbal decoction prepared to 200 mL/day; 100 mL retention enema twice daily	Stool consistency, diarrhea, abdominal pain/distension, tenesmus; composite clinical response incorporating bleeding/occult blood and colonoscopic findings	Post-treatment symptom scores were lower than comparator (P < 0.05); composite response was 94.87% vs 84.61% (P < 0.05)	Safety assessment: NR	Single-center; blinding/allocation concealment and radiotherapy details not reported; NR	[189]
TJ-14 (Hangeshashinto; Ban Xia Xie Xin Tang)	ARE; rectum/radiation proctitis; unspecified; Patients receiving whole-pelvis radiotherapy (eligibility: ≥ 40 Gy, ≥ 1.5 Gy/fraction) who developed CTCAE v4.0 grade ≥ 1 acute radiation enteritis lasting ≥ 2 days	Prospective, multi-institutional (4 centers), single-arm Phase II trial; 40 pre-registered, 22 developed ARE and were treated/analyzed; no comparator	2.5 g p.o. three times daily (7.5 g/day), started after ARE onset	1-week treatment efficacy (no ARE-grade worsening); 2-/3-week efficacy; stool frequency	19/22 (86%) met the 1-week efficacy criterion; mean stool frequency decreased from 4.9 to 3.7/day at 1 week (P = 0.02), 2.2 at 2 weeks (P = 0.004), and 2.1 at 3 weeks (P = 0.05); FACT-G did not further deteriorate after TJ-14 initiation	Grade 1 hypokalemia occurred in 4 patients (2 discontinued TJ-14) and grade 1 constipation in 3; no grade ≥ 2 nonhematologic events	Single-arm study with no comparator/blinding; small, heterogeneous cohort with concurrent chemotherapy; tumor response/radiosensitivity not assessed	[190]
Xiangyu Quyu Mixture retention enema	ARE; rectum/radiation proctitis; prophylactic; Rectal-cancer patients after sphincter-preserving surgery receiving first postoperative IMRT	Single-center randomized controlled prophylaxis study; n = 60 (30/30); random-number table; Xiangyu Quyu Mixture vs sterile-water retention enema	150 mL retention enema q.d., 30 min before each radiotherapy fraction, at 37.5 ± 0.5°C; continued through completion of radiotherapy; comparator used the same schedule with sterile water	Time to acute radiation proctitis; RTOG/EORTC injury grade; body-weight change	Later onset (17.21 ± 3.10 vs 11.53 ± 2.66 days, P < 0.001); lower injury grades (P = 0.006), less weight loss (1.78 ± 0.88 vs 3.21 ± 1.00 kg, P < 0.001), and lower pain score (2.93 ± 2.23 vs 5.53 ± 1.83, P < 0.001)	Safety assessment: NR	Small single-center sample and symptom-based acute-proctitis assessment; allocation concealment/blinding NR; tumor response/radiosensitivity and systematic safety were not evaluated	[166]
Ziwu Liuzhu–timed herbal retention enema	CRE; rectum/radiation proctitis; therapeutic; Cervical-cancer–associated radiation enteritis/proctitis	Single-center controlled study reported as randomized; n = 80 (42/38); same herbal enema in both groups, differing only in administration time; randomization method/blinding/allocation concealment: NR	Adjustable seven-herb decoction prepared as 100-mL bags; retention enema q.d.; for 15 days (5 days/course × 3)	Composite clinical response; time to resolution of diarrhea, abdominal pain, and mucous-bloody stool	Reported response was 88% vs 79%; among responders, symptom-resolution times were shorter with 05:00–07:00 administration (all reported P < 0.01)	Safety assessment: NR	Tests administration timing rather than herbal efficacy because both groups received the same formula; randomization/blinding details NR; formula could be adjusted by condition	[191]

A recurrent preclinical pattern is the preservation of epithelial structure or regenerative capacity together with attenuation of oxidative and inflammatory injury. Berberine provides another relevant example, with network-pharmacology predictions supported by in vivo and in vitro experiments showing improved post-irradiation survival, body weight, intestinal morphology, Ki67/Muc2 staining, and epithelial cell recovery, together with changes in MYC and CCND1 expression in intestinal epithelial and colon cancer cells[164]. Changes in barrier proteins, intestinal stem-cell recovery, and cytokine profiles have been commonly reported, whereas vascular responses, fluid transport, and microbial composition have been examined in smaller subsets of studies. LGYD provides a representative example in which epithelial recovery was accompanied by increased *Akkermansia muciniphila* abundance and short-chain fatty acid levels. Supplementation with *A. muciniphila* in vivo and experiments using its culture supernatant or isobutyric acid in nonirradiated organoids provided partial functional support for this association. However, the absence of microbiota-depletion or fecal microbiota-transfer experiments and of in vivo evidence demonstrating that the microbiota–short-chain fatty acid axis is required for LGYD-mediated protection prevents firm causal attribution [31]. More broadly, concurrent changes in multiple endpoints do not establish that the affected processes are mechanistically interdependent.

The strength of mechanistic evidence also varies substantially among formulas. Anchang Group Prescription represents one of the relatively few whole-formula studies in which pharmacological pathway activation partially reversed the protective phenotype, providing functional evidence for the involvement of PI3K/AKT signaling [33]. In irradiated organoids, Hes1 inhibition partially attenuated the regenerative effects associated with LGYD, but this experiment did not establish that the pathway is required for protection in vivo [165]. For Gegenqinlian Decoction, pathway perturbation was conducted using a defined mixture of selected components rather than the intact formula; the resulting pathway dependence therefore cannot be directly attributed to the complete decoction [32]. Most remaining formula studies provide phenotypic or pathway-associated rather than causal mechanistic evidence. Moreover, the limited number of formula-level cell studies makes it difficult to distinguish direct epithelial effects from systemic, immune, microbial, metabolic, or pharmacokinetic influences.

Clinical evidence is concentrated primarily on pelvic radiotherapy-associated rectal injury and radiation proctitis. The available studies cover different preventive and therapeutic settings and administration routes but do not support a uniform regimen for RE. Xiangyu Quyu Mixture administered by retention enema has been investigated for the prevention of acute radiation proctitis, Modified Shaofuzhuyu Decoction administered by retention enema for the treatment of chronic radiation proctitis, and orally administered Fuzhen Zhixie Decoction for acute or subacute intestinal symptoms [166–168]. Reported endpoints included the incidence or clinical severity of radiation-induced intestinal injury, gastrointestinal symptom scores, quality-of-life measures, symptom-resolution time, and laboratory biomarkers. Only a subset of studies incorporated endoscopic or proctoscopic evaluation of mucosal injury or healing, whereas many relied primarily on symptom-based scales and clinical response criteria. This imbalance, combined with heterogeneous endpoint definitions and assessment schedules, limits comparisons between symptomatic improvement and objective mucosal recovery.

Clinical studies were not assigned a mechanistic-support category. Study-specific safety and design limitations are reported; NR indicates information not reported in the source article. Abbreviations: ARE, acute radiation enteropathy; CRE, chronic radiation enteropathy; CRP, C-reactive protein; CTCAE, Common Terminology Criteria for Adverse Events; KPS, Karnofsky Performance Status; RE, radiation enteropathy; RTOG, Radiation Therapy Oncology Group; RT, radiotherapy; TCM, traditional Chinese medicine. Dosing/time: b.i.d., twice daily; d, day; h, hour; mo, month; p.o., oral administration; q.d., once daily; q.n., nightly; t.i.d., three times daily; wk, week. Retention enema denotes rectal retention administration.

## Herbal extracts and bioactive compounds investigated for RE

Herbal extracts and chemically defined bioactive compounds have been widely investigated for radiation enteropathy (RE), with reported effects involving redox regulation, epithelial regeneration, inflammatory control, gut microbiota, and mitochondrial homeostasis. The clinical evidence is summarized in Table 2, and the in vivo and in vitro evidence is presented in **Tables S3 and S4**, respectively. Most experimental studies have addressed acute or early intestinal injury using single high-dose total-body or abdominal irradiation, whereas anatomically defined radiation proctitis, fractionated irradiation, and established late fibrotic enteropathy have received comparatively limited investigation.

**Table 2:** Clinical evidence for herbal extracts and bioactive compounds in RE

Intervention	Stage/site; population	Design; n; comparator	Dose; route; duration	Endpoints	Core findings	Safety	Study-specific limitations	References
*Aloe Vera* 3% rectal ointment	ARE; rectum/radiation proctitis; prophylactic; Adults with colorectal cancer receiving pelvic external-beam radiotherapy	Preliminary randomized, double-blind, placebo-controlled trial; n = 20 randomized (*Aloe* n = 12, placebo n = 8); permuted-block randomization; 2 withdrawals reported	1 g rectally b.i.d.; from the first day of radiotherapy throughout 6 weeks	RTOG and clinical symptom scores (rectal bleeding/pain, diarrhea, fecal urgency); QOL/HAD; serum CRP	Compared with placebo, diarrhea and RTOG total scores were lower and CRP favored *Aloe Vera*; most other clinical symptom measures did not show significant between-group differences	Rectal ointments were well tolerated; no adverse effects were reported in either group	Small preliminary sample withdrawals; no chemical-constituent profiling or endoscopic confirmation; tumor response/radiosensitivity was not assessedNR	[219]
Modified Baitouweng Decoction alternating with Kangfuxin Liquid retention enema	Unspecified; rectum/radiation proctitis; therapeutic; Radiation proctitis after pelvic/abdominal radiotherapy	Single-center controlled study reported as randomized; n = 100 (50/50); alternating herbal/Kangfuxin enemas vs herbal enema alone; randomization method/blinding NR	Modified Baitouweng decoction 80–100 mL retention enema nightly (retain ≥ 2–6 h), alternating with Kangfuxin Liquid 80–100 mL the next morning (retain ≥ 2–4 h); 1-week course for 4 weeks; optional 2–4-week extension for severe symptoms	Weekly categorical response (cure/improved/no (cure/no response)	Response distribution favored the alternating regimen (P < 0.05); at wk 2, cure occurred in 36/50 vs 18/50 controls	Safety assessment: NR	Single-center; randomization/blinding not reported; NR	[220]
Berberine	ARE; unspecified; prophylactic; Seminoma/lymphoma patients receiving abdominal RT (36 Gy, 1.8 Gy/fraction) and cervical cancer patients receiving whole-pelvic RT (46 Gy, 2.0 Gy/fraction)	Prospective randomized controlled prophylaxis cohorts: n = 36 (18 vs 18) and n = 42 (21 vs 21), berberine vs placebo; additional uncontrolled symptomatic cohort n = 8	300 mg p.o. three times daily p.o.; t.i.d.; during radiotherapy in randomized cohorts	Weekly CTC v2.0 acute GI toxicity incidence, severity, and onset (including colitis, proctitis, diarrhea, nausea/vomiting, and weight loss)	Berberine reduced the incidence/severity and delayed the onset of acute intestinal toxicity versus placebo in both randomized cohorts; symptoms improved after berberine initiation in the uncontrolled symptomatic cohort	No berberine-related toxicity or obvious adverse events were reported; all berberine-treated patients completed therapy	Blinding/allocation concealment not reported; NR; heterogeneous cohorts plus an uncontrolled treatment cohort	[193]
KUSHEN Ningjiaos (matrine/carbopol rectal gel)	Unspecified; rectum/radiation proctitis; prophylactic; Cervical cancer patients (FIGO I–IV) who had completed EBRT and underwent HDR intracavitary brachytherapy with 192Ir	Prospective, single-center, non-randomized open-label paired dosimetric study; n = 100, with planning scans before and after KUSHEN insertion; no independent clinical control group; 57 patients had clinical follow-up	20 g (40 mL; 5 g × 4) inserted transanally into the rectum during brachytherapy planning/treatment; number/frequency of insertions across the brachytherapy course NR	Rectal-wall dose-volume parameters; RTOG-graded acute/chronic radiation proctitis during follow-up; procedure-related adverse events	Posterior rectal-wall maximum dose and D2cc decreased after insertion (both P < 0.05), whereas whole/anterior rectal-wall D2cc increased; among 57 patients followed for a median of 7 mo, acute RP occurred in 15 and chronic RP in 22, all grade 1–2	Insertion was completed successfully in all patients; no procedure- or product-related adverse events were reported	Non-randomized open-label paired dosimetric design with no clinical toxicity comparator; only 57/100 had short follow-up; product chemical standardization was insufficiently described	[221]

Clinical studies were not assigned a mechanistic-support category. Study-specific safety and design limitations are reported; NR indicates information not reported in the source article. ARE, acute radiation enteropathy; CRE, chronic radiation enteropathy; CRP, C-reactive protein; CTCAE, Common Terminology Criteria for Adverse Events; KPS, Karnofsky Performance Status; RE, radiation enteropathy; RTOG, Radiation Therapy Oncology Group; RT, radiotherapy; TCM, traditional Chinese medicine. Dosing/time: b.i.d., twice daily; d, day; h, hour; mo, month; p.o., oral administration; q.d., once daily; q.n., nightly; t.i.d., three times daily; wk, week. Retention enema denotes rectal retention administration

To clarify differences in evidential strength, we distinguish cell- and organoid-based experiments, animal studies, and clinical evidence. We also consider whether the proposed mechanisms are inferred from phenotypic or molecular changes or tested through pharmacological inhibition, gene silencing, transfer, or rescue experiments. These comparisons reflect the depth of experimental validation rather than relative therapeutic efficacy. Clinical evidence remains limited to four small and heterogeneous reports comprising case-report evidence, one multi-institutional prospective single-arm phase II study, and randomized controlled trials [176, 190, 192, 193]. These reports examined different preparations, anatomical settings, and preventive or therapeutic objectives and therefore cannot be directly compared.

### Regulation of oxidative stress and redox homeostasis

Improved redox homeostasis is among the most consistently reported effects of herbal extracts and bioactive compounds in experimental RE. Chemically diverse interventions have been associated with enhanced endogenous antioxidant capacity and reduced ROS accumulation, oxidative macromolecular damage, lipid peroxidation, and intestinal tissue injury [194–199]. Redox regulation therefore appears to be a shared component of experimental protection rather than an effect restricted to a particular chemical class.

The depth of mechanistic support nevertheless varies. Many studies rely on biochemical and histological changes, which associate improved redox status with reduced tissue injury but do not establish pathway dependency. Stronger functional evidence has been reported for EGCG and perillaldehyde, for which pharmacological inhibition of Nrf2 attenuated protection against radiation-induced oxidative injury and ferroptosis [197, 198]. However, these perturbation experiments were conducted mainly in irradiated epithelial cells or organoids and do not by themselves demonstrate Nrf2 dependency within the intact intestinal microenvironment. VND3207 has been linked to DNA-PKcs-mediated DNA repair through loss-of-function experiments in DNA-PKcs-deficient models, extending the evidence beyond conventional antioxidant measurements [200]. Because oxidative injury, mitochondrial dysfunction, epithelial loss, and inflammatory amplification are closely coupled, changes in redox markers alone cannot establish antioxidant activity as the primary protective mechanism.

### Preservation of epithelial regeneration and barrier integrity

Protection of the intestinal barrier may involve crypt preservation, intestinal stem and progenitor cell survival, epithelial proliferation, junctional maintenance, reduced permeability, or protection of the vascular niche. Similar histological improvement may therefore arise through different proximal mechanisms.

The endpoints used vary according to the anatomical setting. Acute small-intestinal and gastrointestinal radiation-injury models generally assess crypt survival, villus architecture, epithelial proliferation, and short-term regenerative capacity [196, 198, 201]. Colonic studies more often examine tight-junction integrity, permeability, epithelial apoptosis, inflammatory infiltration, and mitochondrial injury [202–204]. Findings from acute small-intestinal crypt models therefore cannot be directly extrapolated to radiation proctitis, in which bleeding, telangiectasia, ischemic ulceration, and submucosal remodeling are clinically prominent.

Evidence involving vascular and mesenchymal compartments remains comparatively sparse. Baicalein and ecliptasaponin II have been investigated in relation to radiation-induced endothelial dysfunction and endothelial apoptosis, respectively [201, 205]. Silibinin and Liriodendron, a Fibraureae Caulis–derived extract, have also been evaluated in models involving epithelial–mesenchymal transition, collagen deposition, or delayed intestinal and rectal fibrosis [206, 207]. Together, these findings extend the focus beyond acute epithelial survival, although intermediate collagen accumulation or altered fibrotic markers should not be considered equivalent to established late fibrotic enteropathy.

### Suppression of inflammatory responses and immune dysregulation

Attenuation of radiation-induced inflammation is another recurrent finding. Reported effects include reductions in proinflammatory cytokines, MPO activity, inflammatory-cell infiltration, inflammasome activation, and pyroptotic signaling. The implicated pathways include PPARγ/NF-κB, HMGB1/TLR4, NLRP3, KAT2A-mediated NLRP3 succinylation, and TNF-α/caspase-3 signaling [203, 204, 208–212].

These results support suppression of inflammatory injury, but the relevant cellular targets are not always identifiable. Tissue cytokine concentrations and MPO activity cannot distinguish direct regulation of immune cells from secondary reductions in inflammation caused by improved epithelial integrity and decreased microbial translocation. In addition, many proposed pathways are inferred from altered protein expression without inhibition, genetic manipulation, or rescue experiments. Current evidence consequently provides stronger support for attenuation of acute inflammatory injury than for correction of persistent immune dysregulation during incomplete repair and fibrosis.

### Regulation of gut microbiota composition and function

Several herbal extracts and bioactive compounds have been associated with changes in microbial diversity, community structure, taxonomic abundance, and microbiota-related metabolic profiles following irradiation [210, 213–215]. Most evidence, however, is based on 16S rRNA sequencing and cross-sectional comparisons. Changes in individual taxa or in the Firmicutes/Bacteroidetes ratio do not necessarily indicate restoration of microbial function and may partly reflect improvements in mucosal integrity, inflammation, food intake, or host metabolism.

EGCG is one of the few compounds for which microbiota-related protection has been tested functionally. Protection was transferable through sterile fecal filtrate, and supplementation with the microbiota-derived metabolite D-tagatose reproduced part of the protective phenotype and was accompanied by AMPK signaling activation [215]. These findings show that a transferable microbiota-derived component can be sufficient to reproduce part of the protection under the tested conditions. They do not, however, demonstrate that the microbiota, D-tagatose, or AMPK is required for the overall effect of EGCG. Comparable validation using antibiotic depletion, germ-free models, selective microbial reconstitution, or targeted metabolite manipulation remains uncommon. Reliance on fecal samples also limits conclusions regarding site-specific microbial changes in the small intestine, colon, and rectum.

### Preservation of mitochondrial homeostasis and regulation of apoptosis

Mitochondrial dysfunction is closely linked to radiation-induced oxidative stress, epithelial apoptosis, and impaired regeneration. Earlier studies inferred mitochondrial protection mainly from cytochrome-c release, apoptosis-related proteins, caspase activation, mitochondrial membrane potential, and cell survival [216, 217]. Although these endpoints indicate preservation of mitochondrial integrity, they do not establish a requirement for mitochondrial quality control in intestinal protection.

More recent studies have examined mitophagy-related mechanisms. Icariside II was associated with increased PINK1/Parkin-related mitophagy, improved mitochondrial function, reduced apoptosis, and restoration of colonic barrier integrity [202]. Reversal of cellular protection by Mdivi-1 is consistent with the involvement of mitochondrial dynamics or mitophagy in the observed effects. However, Mdivi-1 is not specific to the PINK1/Parkin pathway, and the absence of direct in vivo pathway perturbation limits causal interpretation.

Licochalcone D has similarly been linked to SIRT3-associated mitophagy, reduced ROS accumulation and DNA damage, suppression of epithelial apoptosis, and preservation of crypt–villus architecture [218]. Pharmacological inhibition of autophagy with chloroquine attenuated protection in irradiated epithelial cells, whereas the SIRT3 inhibitor 3-TYP weakened intestinal structural protection in vivo. SIRT3 knockdown further abolished licochalcone D-mediated protection against radiation-induced DNA damage in epithelial cells. Together, these experiments provide functional support for the involvement of SIRT3-associated mitophagy, although inhibitor specificity and the absence of intestinal cell-specific genetic validation preclude the conclusion that SIRT3 is required for protection in vivo.

### Protective mechanisms of representative compounds

EGCG, icariside II, and licochalcone D illustrate overlapping but differently substantiated protective mechanisms across epithelial injury and repair. For EGCG, Nrf2 inhibition supports the involvement of redox regulation and ferroptosis suppression, whereas sterile fecal-filtrate transfer and D-tagatose supplementation provide separate functional evidence for a microbiota-derived metabolic component. Because these effects were examined in different experimental settings, current evidence does not establish that the redox and microbiota-related pathways act jointly or are both required for the overall protection afforded by EGCG. Icariside II links PINK1/Parkin-associated mitophagy with reduced oxidative injury and apoptosis, attenuation of inflammation, and restoration of colonic barrier integrity; however, pathway involvement was tested mainly with Mdivi-1 in irradiated cells and has not been confirmed through genetic perturbation in vivo. Licochalcone D was examined using both pharmacological inhibition in vivo and SIRT3 knockdown in irradiated epithelial cells, supporting the involvement of SIRT3-associated mitophagy in epithelial protection and tissue repair [197, 202, 214, 215, 218]. Taken together, these studies indicate that individual compounds may affect several related components of radiation injury, but they do not establish coordination, hierarchy, or mutual dependence among the implicated pathways. Differences in the depth of mechanistic validation should not be interpreted as evidence of relative therapeutic efficacy.

## Integrated pharmacological actions and evidence appraisal

Across the reviewed studies, TCM formulas, herbal preparations, and isolated compounds affected several biological domains implicated in radiation enteropathy (RE), including oxidative and mitochondrial injury, epithelial loss and impaired regeneration, barrier dysfunction, inflammatory amplification, microbial disturbance, and tissue remodeling. Changes in these domains frequently occurred in parallel: reduced oxidative or mitochondrial damage was often accompanied by improved epithelial survival, whereas restoration of barrier integrity coincided with reduced microbial translocation and secondary inflammation. Findings involving redox indices, cytokines, tight-junction proteins, intestinal stem-cell activity, microbial metabolites, and tissue architecture are therefore compatible with interactions among these pathological processes [31, 202, 218]. Nevertheless, simultaneous improvement does not establish pharmacological synergy, identify the direction of crosstalk, or demonstrate that all affected processes are required for protection. Multi-target regulation should therefore be regarded as a cross-study interpretive framework rather than a universally validated mechanism shared by all TCM interventions.

The preclinical evidence reviewed here is weighted toward acute or early injury after single-dose total-body or abdominal irradiation. Small-intestinal models commonly assess survival, crypt loss, villus injury, and short-term intestinal stem-cell regeneration, whereas colonic and rectal models more often evaluate permeability, inflammatory infiltration, mucosal damage, collagen deposition, and fibrosis-related signaling. These models provide complementary but non-interchangeable information. Total-body irradiation additionally causes hematopoietic and systemic immune injury, while whole-abdominal irradiation does not reproduce the field geometry, fractionation, organ motion, or rectal dose distribution of contemporary pelvic radiotherapy. Likewise, early collagen accumulation or altered TGF-β/Smad signaling indicates remodeling activity but is not equivalent to established late radiation enteropathy. Models reproducing delayed mucosal, vascular, and fibrotic abnormalities were uncommon among the included TCM studies, although dedicated chronic radiation-proctopathy models are available [222]. Consequently, acute crypt rescue, early histological improvement, and short-term symptom control should not be assumed to prevent chronic ischemic, vascular, or fibrotic complications.

The strength and scope of inference also differ across experimental systems. Cell and organoid models permit controlled analysis of epithelial responses and pathway perturbation but do not reproduce systemic immunity, vascular–stromal interactions, microbiota, or the absorption and metabolism of complex preparations. Animal models preserve more of this biological context, although translation remains limited by heterogeneous irradiation fields, doses, intervention schedules, administration routes, and outcome measures. The clinical reports included in this review comprise a case report, one multi-institutional prospective single-arm phase II study, and randomized controlled trials [176, 190, 192, 193]. These studies provide preliminary evidence of feasibility or clinical benefit but remain constrained by small samples, heterogeneous preparations and endpoints, incompletely reported trial procedures, and limited follow-up. They also provide insufficient evidence regarding tumor response, recurrence, progression, or survival. Studies of isoliquiritigenin and Huanglian Decoction in irradiated lung-cancer-bearing mice demonstrate the feasibility of evaluating intestinal protection in a tumor-bearing setting, but the presence of a tumor alone does not establish oncological safety [203, 223]. Conversely, Guiqi Baizhu Decoction enhanced experimental non-small-cell lung cancer radiosensitivity through inhibition of the HIF-1α/DNA-PKcs axis [224]. This finding is specific to that formula and tumor model and cannot be extrapolated to other preparations. Tumor-bearing studies should therefore assess at least an appropriate measure of tumor response, such as tumor-growth delay or local control, with metastatic progression and survival included where relevant to the model and follow-up duration.

Mechanistic conclusions should be matched to the level of validation performed. Network pharmacology and molecular docking are hypothesis-generating approaches, whereas transcriptomic, metabolomic, microbiome, and protein-expression changes generally remain exploratory or association-based unless linked to functional perturbation. Pharmacological activation or inhibition can support pathway involvement but remains vulnerable to imperfect selectivity and off-target effects. Genetic loss-of-function and rescue experiments can test necessity, while gain-of-function, microbial transfer, or metabolite supplementation may provide evidence of sufficiency. Direct binding and cellular target-engagement assays provide more proximal evidence for proposed molecular targets. Selected studies have progressed beyond descriptive profiling through pathway perturbation, cross-validation among experimental systems, organoid assays, or microbiota- and metabolite-transfer approaches [32, 33, 165, 203]. Nevertheless, in vivo dependence on the proposed mechanism has seldom been established for intact formulas, and evidence obtained from a purified compound cannot be directly extrapolated to its source herb or a multi-herb formula. Mechanistic attribution should therefore remain specific to the preparation, model, tissue, dose, and level of validation examined. Collectively, these limitations constitute major barriers to clinical translation and identify priorities for future preclinical and clinical research.

## Clinical evidence and translational considerations

### Clinical application, treatment routes, and limitations

Study-level details of the clinical setting, design, administration route, endpoints, and reported safety findings are summarized in Table 2. The discussion below therefore focuses on cross-study patterns and interpretive limitations.

The clinical application of TCM for RE should be defined according to disease stage, anatomical site, and therapeutic objective rather than treated as a single indication. The included studies encompassed preventive interventions administered during pelvic or abdominal radiotherapy, treatment of acute radiation-associated diarrhea or proctitis, and management of persistent or chronic rectal symptoms. Oral preparations were mainly evaluated for diffuse intestinal symptoms or acute enteritis, whereas retention enemas and other topical rectal preparations were directed toward localized proctitis. These routes are not interchangeable. Oral efficacy may depend on luminal exposure, gastrointestinal absorption, metabolism, and microbiota-mediated transformation, whereas rectal administration may increase local exposure but is influenced by formulation concentration, administered volume, retention time, lesion distribution, and patient tolerance. Preventive and therapeutic settings should likewise be interpreted separately.

Reported endpoints included stool frequency, diarrhea severity, rectal bleeding, fecal urgency, abdominal or rectal pain, time to symptom resolution, CTCAE- or RTOG-based toxicity grades, composite clinical response, quality of life, laboratory biomarkers, and, in a smaller subset, endoscopic or proctoscopic findings. Although symptom-based outcomes are clinically relevant, symptomatic improvement does not necessarily indicate objective mucosal healing or prevention of delayed vascular and fibrotic injury. Future trials should therefore combine validated patient-reported outcomes and standardized toxicity grading with objective mucosal assessment when clinically justified. Acute and chronic disease should also be evaluated using distinct follow-up schedules and outcome frameworks.

Safety reporting remains insufficient. The prospective TJ-14 study prespecified adverse-event assessment and reported grade 1 hypokalemia and constipation [190]. In several other studies, safety reporting was limited to general statements that no adverse events were observed; predefined monitoring procedures and systematic laboratory surveillance were not described. Such reporting cannot exclude uncommon, delayed, dose-related, or interaction-related toxicity. Future studies should prospectively assess gastrointestinal adverse events, electrolytes, blood counts, hepatic and renal function, adherence, concomitant anticancer therapy, and potential herb–drug interactions. Radiotherapy completion and treatment interruptions should be reported as treatment-delivery outcomes, whereas tumor response, recurrence, progression, and survival should be prespecified as oncological outcomes.

Overall, the clinical evidence remains preliminary. Small samples, heterogeneous preparations, different preventive and therapeutic settings, inconsistent definitions and grading of RE, incomplete reporting of randomization or blinding, variable endpoint schedules, and limited follow-up preclude robust estimates of efficacy and safety. Current findings justify further investigation but are insufficient to establish a standard TCM treatment for RE.

### Herbal composition, chemical characterization, dose translation, and reproducibility

Across the included studies, composition, preparation procedures, chemical characterization, batch information, dose expression, and exposure assessment were not consistently reported together. Some preparations were characterized using chromatographic or mass-spectrometric profiling, but batch-specific release criteria, interbatch comparison, and pharmacokinetic or intestinal-exposure data remained uncommon. Detection of multiple chemical constituents alone does not establish preparation identity, quantitative consistency, biological equivalence, or causal relevance.

Studies of formulas and herbal extracts should report accepted botanical names, medicinal parts, geographical origins, authentication methods, voucher specimens, herb proportions, extraction solvents, extraction temperature and duration, extraction yield, final dry-extract content, storage conditions, and stability. Chromatographic fingerprints should be combined with quantitative assays of predefined quality markers, including quality markers (Q-markers) where scientifically justified. Marker selection should reflect preparation identity, batch consistency, measurable exposure, and plausible relevance to efficacy or safety; not every detected constituent or chromatographic peak should be designated a Q-marker. Specifications should also address moisture, microbial contamination, pesticide residues, heavy metals, mycotoxins, and other preparation-specific contaminants where applicable. Batch numbers should be reported, and key findings should ideally be reproduced using independently prepared batches or batches meeting predefined release specifications.

Dose reporting requires similar standardization. Concentrations expressed only as grams of crude drug per milliliter, percentages of drug-containing serum, or milligrams per kilogram of an incompletely characterized extract do not permit reliable cross-study comparison. Formula doses should therefore be reported as crude-drug equivalents, dry-extract mass, extraction yield, and amounts of quantified markers where available. Animal-to-human conversion based on body-surface area may support initial dose selection but does not account for differences in extraction, absorption, metabolism, microbiota-mediated transformation, or tissue distribution and should not be interpreted as direct clinical dose equivalence. Rectal preparations additionally require reporting of concentration, volume, retention time, dosing frequency, formulation properties, and the intestinal segment exposed.

Pharmacokinetic and intestinal-exposure evidence is particularly important for mechanistic interpretation. Plasma concentrations after oral administration may not reflect luminal, mucosal, microbial, or metabolite-mediated activity. Conversely, constituents detected in serum may not reach irradiated intestinal tissue at concentrations sufficient to reproduce effects observed in vitro. Future studies should therefore relate administered doses to parent compounds, circulating and microbiota-derived metabolites, intestinal tissue concentrations, luminal exposure, and pharmacodynamic responses. These data are necessary to determine whether experimentally implicated mechanisms are plausible at therapeutically relevant exposures.

### Priorities for future preclinical and clinical research

Preclinical research should incorporate clinically relevant fractionated pelvic or rectal irradiation, anatomical dosimetry, and both acute and delayed assessment points. Models should encompass persistent mucosal injury, vascular dysfunction, ulceration, and mature fibrosis rather than relying predominantly on early crypt loss and short-term survival. Intervention timing should distinguish prophylactic administration before or during irradiation, mitigation initiated after exposure, and treatment of established late disease, because these settings address different biological processes and clinical objectives.

Mechanistic studies should test whether a proposed pathway is necessary or sufficient for the effects of the intact, chemically characterized preparation. Tissue- or cell-specific genetic perturbation, pharmacological–genetic cross-validation, rescue experiments, direct target-engagement assays, microbiota depletion and reconstitution, metabolite manipulation, and multicellular organoid or co-culture systems may strengthen causal inference. Functional validation should be reproduced across independent preparation batches and at exposure levels consistent with the corresponding in vivo dose.

Oncological safety requires tumor-bearing models in which normal-intestinal injury and tumor response are assessed concurrently. Relevant outcomes may include tumor-growth delay, local control, metastatic progression, and survival, selected according to the tumor model and follow-up duration. Studies should determine whether the intervention is present systemically during irradiation and whether it alters tumor DNA damage and repair, oxidative responses, antitumor immunity, or malignant stem-cell survival. Potential interactions with concurrent chemotherapy, targeted therapy, or immunotherapy also require evaluation. Demonstrating intestinal protection without an appropriate measure of tumor response is insufficient for an intervention intended for administration during radiotherapy.

Clinical development should proceed through prospectively registered, adequately powered, and preferably multicenter trials using chemically standardized products. Prevention of acute toxicity, treatment of established acute symptoms, and treatment of chronic radiation proctopathy should be evaluated separately. Trials should report sequence generation, allocation concealment, blinding where feasible, adherence, concomitant supportive care, radiotherapy fields and dose–volume parameters, and product batch information. Core outcomes should include validated symptom measures, CTCAE- or RTOG-based toxicity, quality of life, objective mucosal assessment when clinically justified, treatment interruptions, and appropriately timed gastrointestinal and oncological follow-up. Until such evidence becomes available, the reviewed interventions should be regarded as candidate supportive treatments requiring further validation rather than established radioprotective standards.

## Conclusion and perspective

Preclinical studies, conducted mainly in acute or early radiation-injury models, suggest that TCM formulas, herbal preparations, and defined bioactive compounds may modulate several interconnected processes in radiation enteropathy, including oxidative and mitochondrial injury, epithelial and barrier damage, inflammatory amplification, microbial disturbance, and fibrosis-related remodeling. However, these findings do not establish clinical effectiveness. The clinical literature remains sparse and heterogeneous, with small samples, inconsistent interventions and endpoints, incomplete methodological reporting, and limited long-term follow-up. For many complex preparations, the active constituents and their contributions to formula-level effects remain poorly resolved, while mechanistic claims often rely on predicted targets or parallel changes in pathway markers rather than causal validation. Reproducibility is further limited by inadequate control and reporting of botanical sources, preparation procedures, chemical profiles, predefined quality markers, batch consistency, and dose expression, together with limited information on systemic or intestinal exposure. Safety is also insufficiently characterized, particularly regarding delayed toxicity, herb–drug interactions, and effects on tumor control. These interventions should therefore be regarded as investigational rather than established preventive or therapeutic options for RE. Clinical translation will require standardized and reproducible preparations, exposure-relevant causal validation in clinically representative acute and chronic models, and rigorous trials that jointly evaluate gastrointestinal benefit, long-term safety, and oncological outcomes.

## Supplementary Information

Supplementary Material 1

## Data Availability

No datasets were generated or analysed during the current study.

## Abbreviations

AKT: Protein kinase B
ALOX15: Arachidonate 15-lipoxygenase
AMPK: AMP-activated protein kinase
ARE: Acute radiation enteropathy
ATM: Ataxia-telangiectasia mutated
ATR: Ataxia telangiectasia and Rad3-related
ATF4: Activating transcription factor 4
ATP: Adenosine triphosphate
cGAS: Cyclic GMP-AMP synthase
CRE: Chronic radiation enteropathy
CTCAE: Common Terminology Criteria for Adverse Events
CXCL16: C-X-C motif chemokine ligand 16
CXCR6: C-X-C motif chemokine receptor 6
DAMPs: Damage-associated molecular patterns
DNA: Deoxyribonucleic acid
DNA-PKcs: DNA-dependent protein kinase catalytic subunit
DSBs: Double-strand breaks
ECM: Extracellular matrix
EGCG: (-)-Epigallocatechin-3-gallate
eIF2α: Eukaryotic translation initiation factor 2α
GPX4: Glutathione peroxidase 4
H2AX: H2A histone family member X
HD-5: Human defensin 5
HD-6: Human defensin 6
HIF-1α: Hypoxia-inducible factor 1α
HMGB1: High-mobility group box 1
HO-1: Heme oxygenase 1
IECs: Intestinal epithelial cells
IL-6: Interleukin-6
IR: Ionizing radiation
ISCs: Intestinal stem cells
JAK: Janus kinase
LGR5: Leucine-rich repeat-containing G-protein-coupled receptor 5
LGYD: Liangxue Guyuan Yishen Decoction
LPCAT3: Lysophosphatidylcholine acyltransferase 3
LPS: Lipopolysaccharide
M2: M2-like macrophage
MAPK: Mitogen-activated protein kinase
MDA: Malondialdehyde
MDSCs: Myeloid-derived suppressor cells
MPO: Myeloperoxidase
mTOR: Mechanistic target of rapamycin
MyD88: Myeloid differentiation primary response 88
NF-κB: Nuclear factor kappa-B
NLRP3: NOD-like receptor family pyrin domain-containing 3
NOX1: NADPH oxidase 1
Nrf2: Nuclear factor erythroid 2-related factor 2
OGG1: 8-Oxoguanine DNA glycosylase 1
PAI-1: Plasminogen activator inhibitor-1
PDGF-BB: Platelet-derived growth factor-BB
PDGFR-β: Platelet-derived growth factor receptor-β
PERK: Protein kinase R-like endoplasmic reticulum kinase
PI3K: Phosphoinositide 3-kinase
PINK1: PTEN-induced kinase 1
PPARγ: Peroxisome proliferator-activated receptor gamma
Rac1: Rac family small GTPase 1
RE: Radiation enteropathy
ROS: Reactive oxygen species
RT: Radiotherapy
RTOG: Radiation Therapy Oncology Group
SCFAs: Short-chain fatty acids
SIRT3: Sirtuin 3
Smad2/3: SMAD family members 2 and 3
SSBs: Single-strand breaks
STAT3: Signal transducer and activator of transcription 3
STING: Stimulator of interferon genes
TCM: Traditional Chinese Medicine
TGF-β: Transforming growth factor beta
TGF-β1: Transforming growth factor beta 1
Th1: T helper 1 cell
Th17: T helper 17 cell
TIMP-1: Tissue inhibitor of metalloproteinases-1
TLR4: Toll-like receptor 4
TNF-α: Tumor necrosis factor-alpha
Treg: Regulatory T cell
VE-cadherin: Vascular endothelial-cadherin
Wnt: Wingless-type MMTV integration site
YAP/TAZ: Yes-associated protein/transcriptional coactivator with PDZ-binding motif
ZO-1: Zonula occludens-1
