# Supplementary material for: Multi-target network modulation of Traditional Chinese Medicine in radiation enteropathy: mechanistic insights and therapeutic implications
Source: Chin Med. 2026 Nov 2;21:236. doi: 10.1186/s13020-026-01504-y (PMC13726156; doi:10.1186/s13020-026-01504-y)
Supplement: Supplementary file 1 [file 13020_2026_1504_MOESM1_ESM.docx]

**Table S1.** *In vivo* evidence for TCM formulas in radiation-induced intestinal injury

| **Formula** | **Model; irradiation** | **Stage/site; intent** | **Dose; route; timing** | **Core outcomes** | **Mechanism/evidence (target → validation → limitation)** | **Evidence category** | **References** |
| --- | --- | --- | --- | --- | --- | --- | --- |
| Anchang Group Prescription (ACZF) | C57BL/6 mice; 15 Gy single-dose whole-abdominal 6-MV X-ray irradiation | Acute/early injury; colon; therapeutic (assessed over 7 days post-irradiation) | ACZF decoction (4.2 g crude drug/mL), 0.2 mL q.d.; for 7 days, initiated after irradiation; post-IR | Lower DAI with improved body weight, survival, and colon length; reduced colonic ulceration and crypt/epithelial damage; improved barrier integrity | ACZF reduced PI3K and AKT phosphorylation; coadministration of the PI3K activator YS-49 reversed effects on pathway phosphorylation, tight-junction proteins, and cytokines, supporting functional PI3K/AKT involvement; No genetic validation → cytokine assay, pharmacologic inhibitor/activator, transfer/coadministration → Limit.: No genetic validation | Causal/functional | [1] |
| Anchang Recipe | SD rats; 9 Gy single-dose total-abdominal X-ray irradiation | Acute/early injury; colon; therapeutic | 32.85, 65.69, or 131.38 g/kg i.g.; q.d.; for 30 days | Improved general condition/body-weight recovery; attenuated colonic mucosal edema, gland loss, and inflammatory infiltration | Reduced intestinal ROS; decreased Bax and increased Bcl-2, with lower NF-κB at medium/high doses; multi-node expression/biochemical evidence without direct perturbation (pathway-level associative evidence); no direct *in vivo* pathway perturbation. → expression/phenotype assays → Limit.: † | Pathway-level association† | [1] |
| Astragali Radix–Scutellariae Radix combination | C57BL/6 mice; 14 Gy single-dose local abdominal X-ray irradiation | Acute/early injury; colon; therapeutic | Astragali Radix 60 g + Scutellariae Radix 20 g decoction; 0.4 mL i.g.; q.d. | Improved colonic mucosal/crypt morphology and inflammatory infiltration; lower colonic TNF-α/IL-1β with higher IL-10; altered fecal microbiota composition | Reduced inflammatory cytokines accompanied shifts in gut-microbiota composition; ELISA and 16S sequencing only, without microbiota depletion/transfer or causal perturbation (descriptive/associative ( inflammation–microbiota evidence); no direct *in vivo* pathway perturbation.) → ELISA, 16S rRNA, cytokine assay, transfer/coadministration → Limit.: † | Pathway-level association† | [2] |
| Baitouweng Decoction (BTWD) | C57BL/6 mice; 12 Gy single-dose local abdominal 6-MV X-ray irradiation | Acute/early injury; colon; unspecified (assessed after 7 days) | 7.5 g/kg i.g. twice daily i.g.; b.i.d.; for 7 days | Reduced colonic epithelial/crypt injury and collagen deposition; delayed symptom onset and no premature deaths versus 2/8 model deaths; reduced inflammatory and apoptotic injury | Reduced serum IL-6, TNF-α, and COX and shifted Bax/Bcl-2/caspase-3/CytoC expression toward an anti-apoptotic profile; expression analyses only, without pathway perturbation; No direct *in vivo* pathway perturbation; cytokine assay → Limit.: † | Pathway-level association† | [3] |
| Baitouweng Decoction | SD rats; 20 Gy single-dose localized rectal/pelvic X-ray irradiation | Persistent/subacute injury; rectum; therapeutic | Retention enema after model establishment: 1 g/mL (low) or 2 g/mL (high), 1 mL once daily; assessments q.d.; assessment after 3, 7, 14 and 28 days of treatment | Attenuated weight loss and rectal inflammation; reduced collagen deposition/fibrotic remodeling | Downregulated Collagen I, TGF-β1 and Smad3 expression; pathway-associated expression evidence without direct perturbation or causal validation; No direct *in vivo* pathway perturbation; expression/phenotype assays → Limit.: † | Pathway-level association† | [4] |
| Changfukang Decoction | SD rats; sex NR; 9 Gy single-dose whole-abdominal X-ray irradiation | Acute/early injury; ileum; therapeutic (30-d follow-up) | 15.75, 31.50, or 63.00 g crude drug/kg/day i.g. once daily drug/kg/d i.g.; q.d.; for 30 days, initiated after model confirmation | Improved feeding, activity, and stool consistency; higher body weight with the medium dose; milder ileal mucosal injury across doses, with Chiu-grade improvement statistically supported for the medium dose | Pathway: NR; phenotype only → expression/phenotype assays → Limit.: † | Phenotypic/descriptive† | [5] |
| Changhejianye (CHJY) | Adult SD rats; sex NR; 10 Gy single-dose whole-abdominal 6-MV X-ray irradiation | Acute/early injury; multiple sites; therapeutic (assessed on day 8) | 19.8 g/kg i.g.; q.d.; for 7 days, initiated on day 1 after irradiation | Lower diarrhea scores and higher day 8 body weight; 1/12 deaths versus 4/12 in the model group (no survival test reported); descriptively reduced ileal/colonic mucosal injury | Higher Nrf2/HO-1/NQO1 with lower oxidative stress, inflammatory cytokines, Bax, and cleaved caspase-3 and higher Bcl-2; expression/biomarker analyses without direct pathway perturbation (pathway-level associative evidence → cytokine assay → Limit.: † | Pathway-level association† | [6] |
| Changhe Jianye (CHJY) | adult SD rats; 10 Gy single-dose whole-abdominal 6-MV X-ray irradiation | Acute/early injury; multiple sites; therapeutic (assessed on day 8) | 19.8 g/kg i.g.; q.d.; for 7 days, initiated on day 1 after irradiation | Lower diarrhea scores and higher day 8 body weight; 2/12 deaths versus 5/12 in the model group (no survival test reported); descriptively preserved jejunal/ileal morphology | Lower intestinal TLR4 and NF-κB p65 abundance with reduced IL-1, IL-6, and TNF-α; tissue protein/cytokine measurements only, without pathway perturbation (pathway-level associative evidence); no direct *in vivo* pathway perturbation. → cytokine assay → Limit.: † | Pathway-level association† | [7] |
| Changrui enema | C57BL/6 mice; 27 Gy single-dose local pelvic 6-MV X-ray irradiation | Acute/early injury; rectum; therapeutic | Retention enema 1.8, 3.6, or 7.2 g/kg q.d.; from day 2 post-irradiation for 1 or 2 weeks | Improved general-condition scores and body-weight recovery; reduced rectal histopathologic injury | Downregulated IL-1β/NF-κB and VEGF/AQP1/p-ERK1/2, with increased AQP3/p-JNK; consistent with reduced consistent: inflammation-associated angiogenesis, based on WB/IHC expression analyses without direct pathway perturbation → WB, IHC, cytokine assay → Limit.: † | Pathway-level association† | [8] |
| Chinese herbal diet (CHD; Polygonati Rhizoma, Achyranthis Bidentatae Radix, and Epimedii Folium) | C57BL/6 mice, 4 weeks; single 2, 4, or 8 Gy total-body 60Co γ-irradiation | Acute/early injury; small intestine; prophylactic (assessed on day 9) | 3.03 g/kg p.o. once daily p.o.; q.d.; for 26 consecutive days | Greater crypt depth at 4 and 8 Gy and preservation of intestinal nNOS-positive inhibitory neurons at 8 Gy; no improvement in villus length, body weight, hematopoietic indices, or immune-organ indices | Preserved nNOS-positive enteric neurons by immunohistochemistry; descriptive tissue evidence only, without motility testing or pathway, microbiota, pharmacologic, or genetic perturbation; No direct pathway perturbation. → IHC → Limit.: † | Phenotypic/descriptive† | [9] |
| Compound Kushen Injection (CKI) | SD rats, 6 weeks; 10 Gy whole-abdominal 6-MV X-ray irradiation | Acute/early injury; ileum; therapeutic (assessed after 7 days) | 0.1 mL/100 g (1 mL/kg) i.p.; q.d.; for 7 days after irradiation | Improved ileal villus/mucosal histology; reduced inflammatory and oxidative injury; intestinal propulsion rate was not significantly changedNS | Network pharmacology predicted CB1; the CB1 antagonist AM251 attenuated several CKI-associated histologic, inflammatory, oxidative, and p38/NF-κB effects, supporting pharmacologic functional involvement; No genetic or target-engagement validation → network pharmacology, histology → Limit.: † | Causal/functional | [10] |
| Compound Matrine Injection | SD rats; 10 Gy single-dose whole-abdominal 6-MV X-ray irradiation | Acute/early injury; ileum; therapeutic | 0.4 or 2 mL/kg i.p.; q.d.; for 7 days, initiated 12 h after irradiation | Reduced diarrhea and post-irradiation weight loss; preserved ileal villus architecture with greater villus number and height | Lower ileal mucosal NO accompanied protection; evidence was limited to biochemical measurement without pathway perturbation; No direct *in vivo* pathway perturbation; expression/phenotype assays → Limit.: † | Pathway-level association† | [11] |
| Daihuang Shaoyao Powder | Wistar rats; 30 Gy single-dose local pelvic 60Co γ-irradiation | Acute/early injury; rectum; therapeutic (assessed on day 14) | 0.68, 1.36, or 2.72 g crude drug/mL, 2 mL i.g.; q.d.; for 14 days, initiated the day after irradiation | Less gross rectal congestion, ecchymosis, erosion, and ulceration; lower rectal COX-2, serum TXB2, and TXB2/6-keto-PGF1α ratio; 6-keto-PGF1α alone was unchanged versus model | Reduced COX-2 expression and thromboxane-associated imbalance were consistent with improved: microcirculatory injury; biomarker/expression evidence only, without functional pathway perturbation; No direct *in vivo* pathway perturbation; expression/phenotype assays → Limit.: † | Pathway-level association† | [12] |
| Daihuang Shaoyao San (DHSYS) | Wistar rats; 30 Gy single-dose local pelvic 60Co γ-irradiation | Acute/early injury; rectum; therapeutic (assessed on day 14) | 0.68, 1.36, or 2.72 g crude drug/mL; 2 mL/rat i.g.; q.d. | No functional or histologic efficacy endpoint was reported in this paper; rectal NF-κB expression and serum TNF-α/IL-6 were reduced vs model | Lower rectal NF-κB with reduced serum TNF-α/IL-6; multi-node expression evidence consistent with consistent: suppression of NF-κB-associated inflammation, without direct pathway perturbation (pathway-level associative evidence → cytokine assay → Limit.: † | Pathway-level association† | [13] |
| Daihuang Shaoyao San (DHSYS) | Wistar rats; 30 Gy single-dose local pelvic 60Co γ-irradiation | Acute/early injury; rectum; therapeutic (assessed on day 14) | 7.1, 14.2, or 28.4 g/kg i.g.; q.d.; for 14 days, initiated the day after irradiation | High-dose DHSYS increased body-weight gain vs model (P<0.01); food intake/activity improved; rectal epithelial/gland injury and inflammatory infiltration were attenuated | Pathway: NR; phenotype only → expression/phenotype assays → Limit.: † | Phenotypic/descriptive† | [14] |
| Daihuang Shaoyao San (DHSYS) | Wistar rats; 30 Gy single-dose local pelvic 60Co γ-irradiation | Acute/early injury; rectum; therapeutic (assessed on day 14) | 0.68, 1.36, or 2.72 g crude drug/mL; 2 mL/rat i.g.; q.d. | Higher rectal crypt/gland survival, especially at high dose (65.67% vs 19.33% in model, P<0.01); selected thymus/spleen indices improved | IFN-γ, IL-4/IL-10, and lower IFN-γ/IL-4 accompanied increased crypt survival; cytokine-profile evidence consistent with reduced consistent: Th1 bias, without direct Th1/Th2-cell quantification or causal perturbation (associative immunologic evidence); no direct *in vivo* pathway perturbation.) → cytokine assay → Limit.: † | Pathway-level association† | [15] |
| Dunhuang Medical Formula Dabupi Decoction | SD rats; 6 Gy single-dose whole-body X-ray irradiation | Acute/early injury; colon; prophylactic | 6.8 g/kg (0.68 g/mL) i.g.; q.d.; for 14 days before irradiation | Lower colonic mucosal-injury scores with greater mucosal thickness/crypt depth; improved mitochondrial swelling and cytoplasmic vacuolization; body weight, food intake, and water intake were not significantly improved | Reduced ROS, HIF-1α, AQP4 and HOIL-1L; increased PKCζ, Na+/K+-ATPase and occludin. Expression-level, pathway-associative evidence without direct perturbation → cytokine assay → Limit.: † | Pathway-level association† | [16] |
| Fever-reducing and invigorating Chinese medicine (FRICM) | Wistar rats; 10 Gy single-dose abdominal 6-MV X-ray irradiation | Acute/early injury; ileum; prophylactic and therapeutic | Concentrated decoction (2.64 g crude drug/mL) i.g.; q.d.; for 7 days, starting the afternoon after irradiation | Increased ileal villus number and height versus irradiated controls; reduced mucosal NO; no significant differences versus NS or norfloxacin-treated rats | Lower mucosal NO accompanied histologic protection, suggesting reduced: NO-associated inflammatory activity; descriptive/associative biochemical evidence only, without NOS/pathway perturbation or causal validation; No direct *in vivo* pathway perturbation; histology → Limit.: † | Pathway-level association† | [17] |
| Fuzheng Huoxue Qingre Formula | Wistar rats; 20 Gy single X-ray irradiation (irradiation field/source NR) | Unspecified; rectum; therapeutic | 5 mL retention enema every other day q.o.d.; after model establishment; concentration/formula dose and total duration NR | Attenuated rectal mucosal congestion, edema, ulceration, and inflammatory infiltration; lower reported TNF-α/IL-8 inflammatory readouts at later assessmentsassessment | Attenuated TNF-α/IL-8 inflammatory responses; descriptive/associative cytokine and immunohistochemical evidence only, without direct pathway perturbation → IHC, cytokine assay → Limit.: † | Pathway-level association† | [18] |
| Fuzheng Qingre Tang | Wistar rats; 20 Gy single X-ray irradiation; irradiated field/source NR | Acute/early injury; rectum; therapeutic | 5 mL retention enema every other day q.o.d.; for 1 week after model establishment; decoction prepared from 13 herbs in 250 mL water (final concentration NR) | Reduced rectal congestion, edema, ulceration, and inflammatory-cell infiltration versus untreated irradiated controls | Lower serum IL-8 and reduced rectal IL-8 immunoreactivity; descriptive/associative cytokine/IHC evidence without pathway perturbation; No direct *in vivo* pathway perturbation; IHC, cytokine assay → Limit.: † | Pathway-level association† | [19] |
| Modified Gegen Qinlian Decoction | Wistar rats; 20 Gy single-dose abdominal-pelvic irradiation using an electron linear accelerator on treatment day 7 | Acute/early injury; rectum; prophylactic | Retention enema every other day for 28 days; initiated before irradiation and continued post-IR | Higher fecal-occult-blood negativity; improved rectal mucosal histopathology vs irradiated model | Increased SOD/CAT activities, reduced MDA, and increased IL-10; descriptive/associative antioxidant and anti-inflammatory evidence without direct pathway perturbation → redox assays, cytokine assay → Limit.: † | Pathway-level association† | [20] |
| Gegenqinlian Decoction (GQD) | C57BL/6 mice; 10 Gy single-dose whole-abdominal X-ray irradiation | Acute/early injury; small intestine; therapeutic (tissues collected on day 8) | 0.75, 1.5, or 3 g/kg i.g.; for 7 days, initiated after irradiation; post-IR | Lower Chiu score with longer villi and more crypts/goblet cells; reduced crypt apoptosis and enhanced epithelial proliferation; reduced intestinal inflammation and oxidative injury | GQD-associated anti-inflammatory, antioxidant, anti-apoptotic, and proliferative changes were observed; Pharmacologic pathway tests targeted a four-component mixture, not intact GQD, in HIEC-6 cells; the whole-formula mechanism therefore remains pathway-associated with indirect functional support No direct *in vivo* pathway perturbation; expression/phenotype assays → Limit.: † | Pathway-level association† | [21] |
| Guiqi Baizhu Decoction (GQBZD) | SD rats; 6 Gy single X-ray irradiation (250 keV, 13.3 mA; Methods state whole-body irradiation after abdominal exposure) | Acute/early injury; colon; therapeutic (assessed after 7 days) | 4.1, 8.2, or 16.4 g/kg i.g.; q.d.; (10 mL/kg), initiated the day after irradiation for 7 days | Reduced colonic water content, mucosal shedding, and edema; improved gland depth and epithelial/mitochondrial ultrastructure, mainly at medium/high doses; only slight overall weight/food-intake recovery | Reduced ROS/HIF-1α/AQP4 and altered HIF-1α–HOIL-1L–PKCζ/Na+/K+-ATPase markers. siHIF-1α in irradiated NCM460 cells reduced AQP4 and increased Na+/K+-ATPase activity, but GQBZD was not tested *in vitro*; supports injury-pathway involvement, not causal formula action. Functional support was indirect/*in vitro* | Pathway-level association† | [22] |
| Huanglian Decoction (HLD) | C57BL/6 mice, bearing subcutaneous Lewis lung tumors; 4 Gy single-dose tumor-localized X-ray irradiation | Acute/early injury; colon; therapeutic (7-d study) | 5.79, 11.57, or 23.14 g/kg i.g.; q.d.; for 7 days, initiated the day after irradiation | Reduced colonic histopathologic/ultrastructural injury; improved barrier integrity; reduced inflammation and apoptosis | Reduced IL-33/ST2L/TRPA1/Ca2+ signaling and multiple ER-stress markers; supported by tissue expression analyses, a parallel TRPA1-inhibitor group, and a nonirradiated TRPA1-overexpression cell model; *In vivo* pathway-level evidence without direct blockade/rescue of the HLD effect Indirect/contextual evidence:: the intestinal tissue was not directly irradiated. → cytokine assay, pharmacologic inhibitor/activator, overexpression/knockdown → Limit.: nonirradiated TRPA1-overexpression cell model | Indirect/contextual§ | [23] |
| Jiawei Xianglian Pills | SD rats, 2 months; mo; 12 Gy single-dose whole-abdominal X-ray irradiation (12 MV in Methods | Acute/early injury; multiple sites; therapeutic (study stopped on day 5 because of high mortality) | Formula dose NR (converted from a 60-kg adult regimen); 5 mL/kg i.g.; q.d. | Lower symptom scores than model on days 1-3 and relatively milder gross/HE injury; body weight was not improved; 6/10 formula-treated versus 2/10 model rats survived to termination (no survival test reported) | Intestinal TLR4, NF-κB p65, IL-6, and TNF-α were measured by ELISA; formula-versus-model significance was not reported, and final analyzable n was 6 versus 2. vs 2; No pathway perturbation; descriptive evidence with severe attrition/survivor-bias risk No direct *in vivo* pathway perturbation | Pathway-level association† | [24] |
| Liangxue Guyuan Decoction (LGD) | SD rats; 11 Gy single-dose total-body 60Co γ-irradiation | Acute/early injury; unspecified; therapeutic (assessed on day 7) | 9.0 g/kg i.g.; q.d.; for 7 days, initiated the day after irradiation | Less severe diarrhea and higher day 7 body weight; 10/25 deaths versus 14/25 in the model group (no survival test reported); partially normalized fecal microbiota composition | Associated with lower microbial alpha diversity, restored Firmicutes/Bacteroidetes balance, lower Bacteroides, and higher Blautia; 16S rDNA profiling of selected survivors only, without depletion, transfer, or rescue (descriptive/associative ( microbiota evidence); no direct *in vivo* pathway perturbation.) → 16S rRNA, transfer/coadministration → Limit.: † | Pathway-level association† | [25] |
| Liangxue-Guyuan-Yishen Decoction (LGYD) | SD rats; 10 Gy single-dose total-body 60Co γ-irradiation | Acute/early injury; small intestine; therapeutic (assessed on days 3, 5, and 10) | 2.73 or 5.46 g crude drug/mL, 10 mL/kg i.g.; q.d.; from day 1 after irradiation until death or scheduled sampling (27.3 or 54.6 g/kg/day) | Prolonged short-term survival and transiently improved body weight, although day-14 survival remained low; improved small-intestinal histology, crypt/ISC regeneration, and barrier integrity (lower ( D-lactate; higher occludin/claudin-1) | Increased Lgr5/PCNA/Cyclin D1 with Wnt3a/β-catenin/c-Myc changes, and increased tight-junction proteins with MEK/ERK activation; network pharmacology plus WB/RT-qPCR supported pathway association, without direct pathway perturbation → WB, qPCR, network pharmacology → Limit.: † | Pathway-level association† | [26] |
| Liangxue Guyuan Yishen Decoction (LGYD) | C57BL/6J mice; 8.5 Gy single-dose whole-body 60Co γ-irradiation, 70 R/min (5 Gy examined only during microbiota model selection) | Acute/early injury; small intestine; prophylactic | LGYD decoction (9.0 g crude drug/mL), 10 mL/kg i.g.; q.d.; from day -3 to day +5 | Higher body weight and survival after 8.5 Gy; partial restoration of villus/crypt architecture; increased epithelial proliferation and reduced apoptosis | Increased Akkermansia muciniphila abundance and fecal SCFAs, especially isobutyrate, accompanied repair; AKK coadministration and nonirradiated organoid assays with LGYD drug-containing serum, AKK supernatant, or isobutyrate partial functional support; No depletion, FMT/rescue, or genetic test; associative/partial functional support was indirect/*in vitro* | Pathway-level association† | [27] |
| Liangxue Guyuan Yishen Decoction (LGYD) | C57BL/6J mice; 8.5 Gy single-dose total-body 60Co γ-irradiation | Acute/early injury; small intestine; therapeutic (day-5 (d-5 tissue analyses and 2-week survival follow-up) | LGYD decoction (8.06 g crude drug/mL), 10 mL/kg i.g.; q.d.; from day 1 after irradiation until death or scheduled sampling (80.6 g/kg/day) | 40% survival at 2 weeks; short-term weight recovery; increased villus length, Ki67, claudin, and ZO-1 | Increased intestinal Hes1, STAT3/p-STAT3, and AKT/p-AKT with scRNA-seq-supported ISC regeneration; no *in vivo* perturbation; In irradiated organoids, the Hes1 inhibitor JI051 attenuated LGYD-serum-induced growth and pathway changes, providing pharmacologic functional support for Hes1 involvement but no genetic or direct-target validation Functional support was indirect/*in vitro*; no *in vivo* dependency test → RNA-seq/transcriptomics, organoid assay, pharmacologic inhibitor/activator → Limit.: † | Pathway-level association† | [28] |
| Qinghua Liangxue Huluo Decoction | Adult Wistar rats; sex NR; 17.5 Gy single-dose local abdominopelvic 6-MV X-ray irradiation | Acute/early injury; multiple sites; prophylactic (assessed on days 4, 7, and 10) | High dose: 0.08 g·mL−1·kg−1 as reported, 1 mL i.g. twice daily i.g.; b.i.d.; from day -7 | Lower general-symptom scores; higher ileal crypt survival, villus height, and crypt depth; lower rectal Hovdenak histopathology scores | Lower serum COX-2, TNF-α, IL-6, and Hsp90α in the high-dose group; biomarker measurements only, without apoptosis assay or pathway perturbation (descriptive/associative ( anti-inflammatory evidence); no direct *in vivo* pathway perturbation.) → cytokine assay → Limit.: † | Pathway-level association† | [29] |
| Qinghua Yichang Formula (QYF) | C57BL/6J mice; 15 Gy single 6-MV X-ray irradiation; field, source geometry, and dose rate NR | Acute/early injury; unspecified; prophylactic and therapeutic | QYF decoction 4 g/mL, 0.2 mL i.g. once daily; pre-IR arm from day −7 and continued for 14 days after irradiation; post-IR arm after irradiation | Improved intestinal histopathology, with less villus/mucosal injury, gland loss, necrosis, and neutrophil infiltration; QYF plus MCC950 showed the greatest histologic improvement | Reduced NLRP3/Caspase-1/GSDMD and IFN-γ/IL-18/IL-1β, with CD4+/CD8+ shifts. MCC950 produced similar protection; combination further suppressed pathway markers, supporting NLRP3-inflammasome involvement but not causal dependence. No direct *in vivo* pathway perturbation; cytokine assay → Limit.: † | Pathway-level association† | [30] |
| Qingre Liangxue Formula | SD rats; 12 Gy single X-ray irradiation from xiphoid process to anal margin | Acute/early injury; unspecified; therapeutic | 2 g/kg per route i.g., enema, or both (combined group received the same dose by each route), every other day q.o.d.; for 4 weeks after model establishment | Improved activity, food/water intake, and stool condition; higher body weight at 4 weeks; increased wk | Increased SOD activity and reduced MDA, consistent with lower: oxidative stress; descriptive/associative biochemical evidence only, without pathway perturbation; No direct *in vivo* pathway perturbation; redox assays → Limit.: † | Pathway-level association† | [31] |
| Qingre Zhixie Formula (Qingre Zhixiefang) | Wistar rats; 20 Gy single-dose localized X-ray irradiation from xiphoid to pubic symphysis | Acute/early injury; rectum; therapeutic | 8, 12, or 16 g/kg by retention enema (5 mL/rat) every other day for 1 week; started on day 2 after model establishment | Improved general condition/fecal occult blood and rectal histopathology; high- and medium-dose groups showed the clearest maximal benefit | Lower serum MDA and higher SOD activity, consistent with reduced consistent: oxidative stress; descriptive/associative biochemical evidence without direct pathway perturbation → redox assays → Limit.: † | Pathway-level association† | [32] |
| Shaoyao Decoction (SYD) | C57BL/6 mice; 12 Gy single-dose local abdominal X-ray irradiation | Acute/early injury; colon; therapeutic (assessed after 7 days) | 8.56, 17.12, or 25.68 g raw herbs/kg i.g.; for 7 consecutive days after irradiation; post-IR | Reduced colonic epithelial/crypt injury and collagen deposition; reduced TUNEL-positive apoptosis and inflammatory/oxidative injury; restored claudin-1 | Associated with higher Dclk1/ATM/MRE11 and Bcl-2 and lower p53/Bax/caspase-3/CytoC; expression, staining, and serum-marker evidence only, without pathway perturbation; No direct *in vivo* pathway perturbation; expression/phenotype assays → Limit.: † | Pathway-level association† | [33] |
| Shenlingbaizhu Extract | BALB/c mice; 10 Gy single-dose local abdominal 60Co γ-irradiation | Acute/early injury; jejunum; prophylactic and therapeutic | 0.75, 1.5, or 3 g/kg i.g.; q.d.; for 10 days, initiated immediately after irradiation | Higher 30-day survival, with no deaths at 1.5 g/kg versus 7/10 deaths in irradiated controls; less acute diarrhea and weight loss; reduced jejunal mucosal injury with greater epithelial thickness/villus height | Pathway: NR; phenotype only → expression/phenotype assays → Limit.: † | Phenotypic/descriptive† | [34] |
| Tongxie Yaofang | SD rats; 10 Gy single-dose whole-abdominal 6-MV X-ray irradiation | Acute/early injury; jejunum; therapeutic | 4.92 g/100 g body weight; i.g., 2 mL/100 g body weight q.d.; from post-irradiation day 1 for 7 days | Improved general condition, mucosanguineous stools and body-weight recovery; ameliorated jejunal mucosal injury | Reduced jejunal NO, IL-6 and TNF-α and increased IL-10; descriptive/associative inflammatory evidence without direct pathway perturbation → cytokine assay → Limit.: † | Pathway-level association† | [35] |
| Tong-Xie-Yao-Fang (TXYF) | SD rats; 10 Gy single-dose whole-abdominal irradiation; radiation source/type unclear in the reportNR | Acute/early injury; jejunum; therapeutic | 4.92 g/100 g body weight; i.g., 2 mL/100 g body weight q.d.; from post-irradiation day 1 for 7 days | Improved general condition and food intake; increased villus height and total intestinal-wall thickness; crypt depth and mucosal thickness were not significantly changedNS | Transcriptomic profiling identified 90 DEGs; enrichment suggested modulation of inflammatory pathways, especially TNF signaling (including Tab3/Bcl3/Tnfaip3 changes) → RNA-seq/transcriptomics → Limit.: † | Pathway-level association† | [36] |
| Modified Xijiao Dihuang Decoction (MXDD) | SD rats; 11 Gy single 60Co γ-irradiation; field and dose rate NR | Acute/early injury; small intestine; therapeutic (assessed on day 3) | 36.0 g/kg q.d.; for 3 days after irradiation | Attenuated day-3 weight loss and diarrhea; partially restored gut microbial diversity/composition, with higher Firmicutes and lower Proteobacteria and Escherichia-Shigella | Microbiota shifts accompanied lower intestinal IL-17/RORγt and higher FoxP3; 16S sequencing, mRNA/protein assays, and correlations only, without depletion, transfer, rescue, or immune perturbation; No direct *in vivo* pathway perturbation; 16S rRNA, cytokine assay, transfer/coadministration → Limit.: † | Pathway-level association† | [37] |
| Xilei Powder + Yunnan Baiyao gel | SD rats; 25 Gy single-dose local pelvic/rectal 6-MV X-ray irradiation | Acute/early injury; rectum; therapeutic | Xilei Powder + Yunnan Baiyao in 1.5% sodium-hyaluronate gel; 1.5 mL retention enema q.d.; for 14 days from day 2 after irradiation | Lower clinical-symptom and rectal-histology scores vs model; overt bleeding was absent in the gel group; rectal injury was milder than with the non-gel solution | Lower serum TNF-α/IL-6 accompanied phenotypic improvement; descriptive/associative inflammatory-biomarker evidence only, without direct pathway perturbation → cytokine assay → Limit.: † | Pathway-level association† | [38] |
| Xuebijing Injection (XBJ) | C57BL/6 mice; 8.0 or 9.0 Gy total-body gamma irradiation; 9.0 Gy used for intestinal analyses | Acute/early injury; small intestine; unspecified (tissues assessed at 3-3.5 days) | 0.4 mL/kg/day i.p. mL/kg/d i.p.; for 5 days (survival experiments) or 3 days (other experiments); start time relative to irradiation NR | Higher day-6 survival and longer median survival after 8.0/9.0 Gy; preserved crypt-villus structure and Lgr5/Ki67-positive regenerative cells; reduced intestinal apoptosis and DNA damage | Altered inflammatory cytokines and reduced γ-H2AX, p53, Bax, and caspase-associated apoptosis; supported by expression/staining changes without pathway perturbation; No direct *in vivo* pathway perturbation; cytokine assay → Limit.: † | Pathway-level association† | [39] |
| Yinhuo Tang (YHT) | ICR mice; 7.0 Gy single-dose total-body 60Co γ-irradiation | Acute/early injury; small intestine; prophylactic (assessed on day 3) | 250, 500, or 1500 mg/kg for 14 days before irradiation; frequency and route NR (prepared as an oral solution) | Preserved small-intestinal villus height, crypt depth, and mucosal architecture; body weight did not differ significantly NS among groups | Systemic serum metabolomics and bone-marrow proteomics/WB showed altered oxidative/metabolic profiles and lower p-PI3K/PI3K and p-Akt/Akt. Evidence p-Akt/Akt; was not intestine-specific and lacked pathway perturbation; network pharmacology/docking were predictive No direct *in vivo* pathway perturbation; WB, metabolomics, network pharmacology, molecular docking → Limit.: † | Pathway-level association† | [40] |

Notes: Evidence categories are causal/functional (pathway-dependency perturbation in the relevant irradiated model), pathway-level association (target/pathway readouts without a dependency test), phenotypic/descriptive (outcomes without pathway validation), and indirect/contextual (nonirradiated or non-RE models). *In vitro* functional validation, where cited as supporting evidence, does not establish *in vivo* causality.

Common limitations: † no mechanism-dependency perturbation in the original article; ‡ *in vitro* functional validation is not equivalent to *in vivo* causality; § nonirradiated/non-RE models provide contextual rather than radioprotective evidence. Expression changes, omics, network pharmacology, and docking are hypothesis-generating unless functionally perturbed in the relevant irradiated model.

Abbreviations: DEG, differentially expressed gene; IR, irradiation; NR, not reported; post-IR, after irradiation; pre-IR, before irradiation; h, hour; d, day; wk, week; mo, month; i.g., intragastric gavage; i.p., intraperitoneal injection; i.v., intravenous injection; p.o., oral administration; s.c., subcutaneous injection; q.d., once daily; q.o.d., every other day; b.i.d., twice daily; t.i.d., three times daily; q.n., nightly; SD, Sprague–Dawley; DAI, disease activity index; SCFA, short-chain fatty acid. Symbols: ↑, increase/upregulation; ↓, decrease/downregulation; →, evidence chain.

**Table S2.** *In vitro* evidence for TCM formulas in radiation-induced intestinal injury

| **Formula** | ***In vitro* model** | **Irradiation conditions** | **Dose; timing** | **Core outcomes** | **Mechanism/evidence (target → validation → limitation)** | **Evidence category** | **References** |
| --- | --- | --- | --- | --- | --- | --- | --- |
| Gegenqinlian Decoction (GQD) | HIEC-6 normal human intestinal epithelial cells | Single 4 Gy X-ray exposure (6-MV linear accelerator; 400 MU/min) | 0.4, 0.8, or 1.6 mg/mL added immediately post-IR; 24 h incubation | Reduced TNF-α, IL-1β, and IL-6 and increased IL-10; restored Ki67 and reduced TUNEL positivity after irradiation | The intact decoction was evaluated phenotypically; NF-κB, Caspase-9/-3, and Wnt/β-catenin perturbation experiments used the defined four-component mixture rather than GQD; Whole-formula mechanism association only → expression/phenotype assays → Limit.: Whole-formula mechanism remains associative | Pathway-level association† | [21] |
| Huanglian Decoction (HLD) | NCM460 human colonic epithelial cells with lentiviral TRPA1 overexpression | No *in vitro* irradiation; genetic overexpression model | 5% or 10% HLD drug-containing rat serum for 48 h | Restored proliferation; reduced overexpressed TRPA1, intracellular Ca2+, Ca2+, ER-stress markers, inflammatory proteins, and apoptosis | TRPA1 overexpression induced proliferation loss, Ca2+ elevation, ER stress, inflammation, and apoptosis, all attenuated by HLD-containing serum; Genetic/functional support for TRPA1-driven stress, but no direct *in vitro* radioprotection, target-necessity test, or binding → overexpression/knockdown → Limit.: target-necessity or binding evidence; indirect/contextual evidence: the mechanistic model was not directly irradiated; binding evidence Indirect/contextual evidence: the mechanistic model was not directly irradiated | Indirect/contextual§ | [23] |
| Liangxue Guyuan Yishen Decoction (LGYD) | Murine small-intestinal crypt-derived organoids (500 crypts/well) | No *in vitro* irradiation; organoids were nonirradiated | 1% or 5% LGYD drug-containing rat serum during organoid culture; organoid area assessed on days 1 and 5; exact exposure start NR | At day 5, 1% LGYD-containing serum increased organoid surface area/formation versus normal serum; 5% serum inhibited growth; no irradiated-organoid outcome | LGYD-related constituents (1% serum) promoted organoid growth in a nonirradiated assay without pathway perturbation; indirect/contextual evidence, not direct *in vitro* radioprotection → organoid assay; Limit.: nonirradiated; no pathway perturbation | Indirect/contextual§ | [27] |
| Liangxue Guyuan Yishen Decoction (LGYD) | Murine small-intestinal Lgr5-GFP crypt-derived organoids (500 crypts/well) | Single 8.5 Gy irradiation (70 R/min), 24 h after implantation | 1% LGYD drug-containing rat serum added before irradiation; pre-IR; assessed on days 1, 3, and 5 | Increased post-irradiation organoid area/formation and Ki67, ZO-1, and Hes1; JI051 counteracted organoid recovery | JI051 attenuated LGYD-serum-induced organoid growth and reduced Hes1, STAT3/p-STAT3, and AKT/p-AKT, supporting pharmacologic functional involvement of Hes1; no genetic rescue or direct target-engagement evidence *In vitro* functional validation only; not equivalent to *in vivo* causality.→ organoid assay, pharmacologic inhibitor/activator → Limit.: no genetic rescue | Causal/functional (*in vitro*)‡ | [28] |
| Shenlingbaizhu Extract | Rat IEC-6 intestinal epithelial cells | Single 16 Gy 60Co γ-irradiation; 217 cGy/min | 12.5, 25, 50, 75, 100, or 150 μg/mL added immediately post-IR; 48 h exposure | Promoted post-irradiation proliferation at 50–150 μg/mL; maximal increase (~20%) at 75 μg/mL; proliferative effect declined at higher concentrations | Pathway: NR; phenotype only → expression/phenotype assays → Limit.: † | Phenotypic/descriptive† | [34] |

Notes: Evidence categories are causal/functional (pathway-dependency perturbation in the relevant irradiated model), pathway-level association (target/pathway readouts without a dependency test), phenotypic/descriptive (outcomes without pathway validation), and indirect/contextual (nonirradiated or non-RE models). *In vitro* functional validation does not establish *in vivo* causality.

Common limitations: † no mechanism-dependency perturbation in the original article; ‡ *in vitro* functional validation is not equivalent to *in vivo* causality; § nonirradiated/non-RE models provide contextual rather than radioprotective evidence. Expression changes, omics, network pharmacology, and docking are hypothesis-generating unless functionally perturbed in the relevant irradiated model.

Abbreviations: DEG, differentially expressed gene; IR, irradiation; NR, not reported; post-IR, after irradiation; pre-IR, before irradiation; h, hour; d, day; wk, week; mo, month; DAI, disease activity index; SCFA, short-chain fatty acid. Symbols: ↑, increase/upregulation; ↓, decrease/downregulation; →, evidence chain.

**Table S3.** *In vivo* evidence for herbal extracts and bioactive compounds in radiation-induced intestinal injury

| **Extract/compound** | **Source** | **Model; irradiation** | **Stage/site; intent** | **Dose; route; timing** | **Core outcomes** | **Mechanism/evidence (target → validation → limitation)** | **Evidence category** | **References** |
| --- | --- | --- | --- | --- | --- | --- | --- | --- |
| 6-Shogaol | Ginger oleoresin; purified by preparative HPLC (98.4% purity) | C57BL/6J mice; 15 Gy single-dose abdominal γ-irradiation; radiation source/dose rate NR | Acute/early injury; small intestine; prophylactic | 100 mg/kg/day by oral gavage q.d.; for 7 days before irradiation (pre-IR, days −7 to −1) | Improved 30-day survival (90% vs 40%) and body-weight recovery; reduced diarrhea and preserved duodenal crypt–villus architecture; improved epithelial permeability and reduced bacterial/endotoxin translocation | Reduced oxidative stress, epithelial apoptosis, IFN-γ/iNOS/TNF-α expression, and bacterial/endotoxin translocation; associative evidence without direct pathway perturbation → cytokine assay → Limit.: † | Pathway-level association† | [41] |
| Acidic polysaccharide of *Panax ginseng* (APG; ginsan) | *Panax ginseng* C.A.Mey. root; acidic polysaccharide (ginsan) | C57BL/6 mice; 2 or 7 Gy single-dose whole-body 60Co γ-irradiation; 7 Gy for crypt regeneration, 2/7 Gy for apoptosis assays | Acute/early injury; jejunum; prophylactic | 10 mg/kg i.p.; on day −1 and again on the day of irradiation; exact timing of the day of irradiation dose relative to exposure NR | Preserved villus length and increased surviving/regenerating crypt cells; reduced radiation-induced jejunal crypt apoptosis | Reduced p53/Bax and increased Bcl-2 in irradiated intestine; descriptive/associative apoptosis-pathway evidence without direct pathway perturbation → expression/phenotype assays → Limit.: † | Pathway-level association† | [42] |
| Aloperine (ALO) | *Sophora alopecuroides* L. (reported natural source); commercial ALO, purity ≥98% | C57BL/6 mice, 6 weeks; 13 Gy single-dose targeted gastrointestinal X-ray irradiation | Acute/early injury; ileum; unspecified | 10 mg/kg by oral/intragastric administration q.d.; for 7 consecutive days; exact start time relative to irradiation NR | Reduced body-weight loss and ileal MPO activity; preserved epithelial structure/villus height; reduced ileal Fe2+ accumulation and lipid peroxidation | TAS2R138 target engagement was supported by docking/CETSA; TAS2R138 knockout in IEC-6 cells weakened ALO-induced metabolic and anti-ferroptotic effects, with GFPT1 knockdown/GAPDH overexpression supporting the PLCβ/GAPDH–HBP axis (genetic/functional evidence mainly *in vitro* | Pathway-level association† | [43] |
| Amentoflavone (AF) | NR (commercial amentoflavone from Sigma; botanical source of the test material not reported) | SD rats; 14 Gy single-dose whole-abdominal high-energy X-ray irradiation | Acute/early injury; ileum; therapeutic | 60 mg/kg/day i.p.; for 7 consecutive days after irradiation; post-IR | Preserved ileal mucosal architecture; reduced collagen-fiber deposition; body weight was not significantly improved versus irradiated controls | Reduced ileal NF-κB mRNA and serum TNF-α/IL-1β, with accompanying gut-microbiota shifts; associative/pathway-level evidence only, without NF-κB perturbation or microbiota-causality testing | Pathway-level association† | [44] |
| Apigenin | NR (commercial apigenin [Innochem]; specific botanical source not reported) | C57BL/6J mice; X-ray TBI/TAI; 10 Gy TBI and 12 Gy TAI survival models; 8–14 Gy TBI crypt-survival series, with 10 Gy used for most tissue assays | Acute/early injury; unspecified; prophylactic | 200 mg/kg i.p.; at 12 h and 2 h before irradiation (two doses) | Prolonged survival after lethal TBI/TAI; reduced weight loss; increased crypt survival and villus regeneration | Increased intestinal Nrf2/HO-1 with reduced lipid peroxidation and apoptosis; pathway-level association only, without Nrf2 perturbation; No direct *in vivo* pathway perturbation | Pathway-level association† | [45] |
| Astragalus polysaccharide (APS) | *Astragalus membranaceus* / *A. membranaceus* var. mongholicus (reported botanical source context); commercial APS, purity ≥95% (batch A1427) | SD rats, 8 weeks; 9 Gy single-dose whole-abdominal 6-MV X-ray irradiation | Unspecified; small intestine; therapeutic | 400 mg/kg/day by oral gavage for 7 days, initiated the day after successful model establishment; mechanistic arm: Compound C 10 μmol/L, 2 mL i.g.; 4 h after APS | Reduced DAI (diarrhea/bleeding) and mucosal permeability; improved small-intestinal histopathology/ultrastructure; reduced mucosal IL-1β and TNF-α | APS increased p-AMPK and reduced p-mTOR; the AMPK inhibitor Compound C partially reversed APS-associated phenotypic and pathway changes, supporting functional AMPK involvement (pharmacologic inhibition evidence) → pharmacologic inhibitor/activator → Limit.: † | Causal/functional | [46] |
| Atractylenolide II (ATR II) | *Atractylodes macrocephala* Koidz rhizome (reported natural source); commercial ATR II (Chengdu DeSiTe), purity NR | C57BL/6J mice; 8 Gy single-dose local abdominal 137Cs γ-irradiation | Acute/early injury; unspecified; prophylactic | 60 mg/kg by oral gavage q.d.; for 6 days before irradiation | Preserved intestinal architecture and reduced villus damage at day 5 after irradiation | MAPKp38/Nrf2–HO-1/NQO-1 involvement was functionally/genetically validated only in irradiated HaCaT keratinocytes (SB203580; Nrf2 siRNA) | Pathway-level association† | [47] |
| Baicalein | *Scutellaria baicalensis* dry root (reported natural source); commercial baicalein (Sigma-Aldrich), purity NR | C57BL/6 mice, 7 weeks; 13.5 Gy single-dose whole-abdominal X-ray irradiation | Acute/early injury; small intestine; therapeutic | 10 mg/kg/day i.p., initiated after irradiation and continued post-IR for 6 days | Preserved villus length and crypts; improved intestinal barrier function and reduced bacterial translocation; reduced inflammatory-cell infiltration | Reduced endothelial P-selectin/ICAM-1/VCAM-1 in irradiated intestine and HUVECs, with lower leukocyte infiltration and inflammatory mediators; cross-model pathway-level associative evidence without target perturbation; No direct *in vivo* pathway perturbation | Pathway-level association† | [48] |
| Baicalein | Plant-derived flavonoid (specific botanical source of the test article NR); commercial baicalein (Aladdin), purity NR | C57BL/6J mice; 137Cs γ-irradiation: 10 Gy survival model and 9 Gy intestinal-injury model; irradiation field NR | Acute/early injury; small intestine; prophylactic | 100 mg/kg i.p.; at 1 h before irradiation and again 1 day after irradiation | Prolonged survival after 10 Gy; preserved crypt–villus architecture and intestinal stem/progenitor regeneration after 9 Gy; reduced intestinal apoptosis | 16S/KEGG and intestinal assays linked protection to microbiota remodeling and reduced p53-associated apoptosis (p53/8-OHdG/Bax/caspase-8/3); pathway-level associative evidence without microbiota or p53 perturbation; No direct *in vivo* pathway perturbation (p53/8-OHdG/Bax/caspase-8/3) → 16S rRNA → Limit.: † | Pathway-level association† | [49] |
| Berberine (BBR; berberine chloride) | *Coptis* (reported natural source); commercial berberine chloride (MedChemExpress) | C57BL/6 mice; 8 Gy single-dose whole-abdominal X-ray irradiation | Acute/early injury; small intestine; unspecified | 5 mg/kg/day i.p.; for 6 consecutive days; dosing began before WAI, but exact distribution relative to day 0 is inconsistently described/depicted | Reduced post-WAI weight loss and intestinal hyperemia/edema; preserved intestinal length, villus/crypt architecture, and crypt-cell survival; increased OLFM4+ ISCs and Lgr5-lineage progeny | Increased p-S6, p-STAT3, and p-ERK1/2-positive crypt cells; lineage tracing supports enhanced ISC regeneration, but signaling evidence remained pathway-level/associative without direct pathway perturbation/associative → expression/phenotype assays → Limit.: † | Pathway-level association† | [50] |
| Berberine (BBR) | *Coptis* and *Berberis* spp. (reported natural sources); commercial berberine from Chengdu Refines Biotechnology | SD rats; 12 Gy single-dose whole-abdominal 60Co γ-irradiation over 3 min (xiphoid to pubic symphysis; source distance 50 cm; dose rate NR) | Acute/early injury; unspecified; therapeutic | 50 mg/kg/day by oral gavage q.d.; for 7 days; timing relative to irradiation NR | Improved stool/bloody-stool findings and body-weight course; preserved intestinal structure and crypts with less collagen deposition; reduced inflammatory and oxidative injury | Reduced IL-6/TNF-α/IL-1β/LPS and MDA with increased SOD; descriptive/associative inflammatory and oxidative-stress evidence without pathway perturbation; No direct *in vivo* pathway perturbation | Pathway-level association† | [51] |
| Bixin | *Bixa orellana* L. seeds (reported natural source); commercial bixin used (Biozol) | C57BL/6 mice; 13.5 Gy single-dose abdominal X-ray irradiation | Acute/early injury; small intestine; therapeutic | 62.5 mg/kg/day i.p., initiated immediately after irradiation and continued post-IR for 6 days | Attenuated weight loss and histopathologic injury; restored villus length, crypt number, Ki67/Olfm4-positive regenerative cells and epithelial barrier markers; reduced mesenteric-lymph-node bacterial translocation | Enhanced NRF2 expression and reduced inflammatory-cell accumulation/TNF-α/PAI1, with restoration of villin/CLDN3/ZO1; pathway-level association without direct NRF2 perturbation or causal validation; No direct *in vivo* pathway perturbation | Pathway-level association† | [52] |
| *Brownea grandiceps* leaf hydro-alcoholic extract (BGE) and ethyl acetate fraction (EAF) | *Brownea grandiceps* Jacq. leaves (Fabaceae); botanically authenticated, voucher PHG-P-BG-167 | SD rats; 10 Gy single-dose whole-body γ-irradiation using a Gamma Cell-40 137Cs source, 0.382 Gy/min | Acute/early injury; jejunum; prophylactic | BGE or EAF 100, 200, or 400 mg/kg p.o. once daily p.o.; q.d.; for 10 days | BGE 400 mg/kg reduced jejunal histologic injury and improved crypt regeneration; EAF, especially 200–400 mg/kg, more strongly restored villus/crypt architecture and goblet cells | Improved intestinal redox markers and reduced NF-κB p65/TNF-α; EAF also lowered TGF-β1. supported by TGF-β1 | Pathway-level association† | [53] |
| Catalpol | *Rehmannia glutinosa* roots (reported natural source); commercial catalpol >99% | BALB/c mice; 8 Gy single-dose abdominal 60Co γ-irradiation | Acute/early injury; small intestine; prophylactic | 25, 50, or 100 mg/kg i.p., single dose 20 min pre-IR | Dose-dependently reduced small-intestinal villus injury; decreased intestinal oxidative DNA damage and improved antioxidant status | Increased SOD/GSH and reduced MDA/intestinal 8-OHdG; descriptive/associative oxidative-stress evidence without direct ROS/pathway perturbation; No direct *in vivo* pathway perturbation | Pathway-level association† | [54] |
| *Centella asiatica* preparation (CA) | *Centella asiatica*; commercial USP preparation | C57BL/6 mice; 13.5 Gy single-dose abdominal X-ray irradiation | Acute/early injury; ileum; therapeutic | CA 200 µg/kg/day, started within 2 h after irradiation and continued post-IR for 6 days; route NR | Attenuated weight loss; restored villus length/crypt number and reduced histologic injury; decreased bacterial translocation and restored epithelial barrier markers | Promoted endothelial–epithelial repair involving endothelial-derived EGF; CA-conditioned medium and rEGF reproduced protection in irradiated mice, while EGF neutralization abrogated barrier rescue *in vitro* (functional/mechanistic evidence). | Pathway-level association† | [55] |
| Crocetin | *Crocus sativus* L. flower stigmas (reported natural source); test-material purity NR | C57BL/6 mice; 6-MV X-rays; total irradiation dose and field NR | Acute/early injury; multiple sites; prophylactic | 25 or 50 mg/kg by oral gavage 1 h before irradiation, pre-IR, followed by daily administration for 3 consecutive days | Preserved crypt–villus architecture; reduced mucosal edema and intestinal bacterial burden; increased villus height and crypt number | Crocetin reduced KAT2A expression and NLRP3 succinylation; sh-KAT2A, NLRP3-K21 mutation and NLRP3-overexpression rescue established a causal KAT2A–NLRP3 succinylation/pyroptosis axis in HIECs, but direct dependence of crocetin protection on KAT2A was not tested | Pathway-level association† | [56] |
| Curcumin | *Curcuma longa* (turmeric; reported natural source); test-material supplier/purity NR | Wistar albino rats; whole-abdominal 60Co γ-irradiation, 5 Gy once or 5 Gy × 2 | Acute/early injury; ileum; prophylactic | 100 mg/kg by oral gavage once daily; q.d.; single-dose arm: 10 days before irradiation through 4 days after (14 days total) | Reduced ileal histopathologic injury and ultrastructural degeneration; attenuated excess goblet-cell responses; limited radiation-associated body-weight loss | Radioprotection was supported by histology, morphometry, and electron microscopy; no molecular pathway or oxidative-stress mechanism was directly tested (descriptive evidence); no direct pathway perturbation. → histology → Limit.: † | Phenotypic/descriptive† | [57] |
| Cynaroside (luteoloside; luteolin-7-O-glucoside) | *Lonicera* species (reported natural source); commercial purified cynaroside >98% | C57BL/6 mice; 12 Gy single-dose total-abdominal 6-MV X-ray irradiation | Acute/early injury; multiple sites; therapeutic | 20, 50, or 100 mg/kg by oral gavage; first dose 2 h after TAI, then daily for the next 5 days; survival arm: 50 mg/kg with continued daily dosing for 20 days | Improved survival and body-weight recovery; preserved colon length and crypt–villus architecture with enhanced epithelial proliferation and regeneration; reduced intestinal inflammation | Proteomics and irradiated rectal-cancer mucosa supported Dnm2 upregulation; dynasore phenocopied protection and Cyn + dynasore showed no added benefit, supporting dynamin-GTPase/Dnm2 involvement; dynasore is not Dnm2-selective and no genetic validation was performed | Pathway-level association† | [58] |
| Defined core-component mixture: puerarin, baicalein, berberine, and glycyrrhetinic acid (molar ratio 254:186:14:1) | Commercially purchased purified compounds; specific botanical source NR | C57BL/6 mice; 10 Gy single-dose whole-abdominal X-ray irradiation | Acute/early injury; small intestine; therapeutic (tissues collected on day 8) | Low-, medium-, or high-dose mixture i.g. for 7 d post-IR | Lower Chiu score with longer villi, more crypts/goblet cells, less apoptosis, and more Ki67-positive crypt cells; reduced inflammatory and oxidative injury | Network pharmacology predicted inflammatory, apoptotic, and proliferative pathways; animal data were phenotypic/biomarker-based, while pathway perturbation was performed only in HIEC-6 cells. | Pathway-level association† | [21] |
| *Dendrobium huoshanense* flavone fraction (FLA) | *Dendrobium huoshanense* stems; flavone fraction isolated by polyamide chromatography and characterized by HPLC fingerprint/PCA/SESC and LC-MS | BALB/c mice; 9 Gy single-dose irradiation used to establish a rectal radiation-enteritis model; irradiation source/dose rate NR in the main Methods | Mixed; rectum; therapeutic | 28.75 mg/kg/day i.g. mg/kg/d i.g.; for 30 days after modeling | Reduced diarrhea/fecal moisture; improved rectal histopathology and lymphocyte infiltration; reduced collagen deposition/fibrotic remodeling | Multi-omics associated FLA with gut-microbiota/metabolite shifts, restored RUNX3/IFN-γ-related immune signaling, and reduced Smad2/3 with increased Smad7/E-cadherin; pathway-level associative evidence without microbiota or pathway perturbation; No direct *in vivo* pathway perturbation | Pathway-level association† | [59] |
| Eclipta total saponins (ETS); Ecliptasaponin II (Ecl2) | Ecliptae Herba (authenticated); ETS prepared from the herb; purified Ecl2 ≥98% | C57BL/6J mice; 13 Gy single-dose abdominal-pelvic ionizing irradiation; radiation source/type/dose rate NR | Acute/early injury; colon; therapeutic | ETS 100 mg/kg or Ecl2 50 mg/kg by oral gavage q.d.; for 7 days; Ecl1 20 mg/kg served as an inactive/weak comparator | Reduced DAI and colonic histopathologic/MPO injury; preserved colon length; reduced intestinal permeability | Ecl2 increased PPARγ-associated downstream transcription in colon; PPARγ dependence was functionally supported only in HUVECs with GW9662, so *in vivo* evidence remains pathway-level rather than causal No direct *in vivo* pathway perturbation | Pathway-level association† | [60] |
| Emodin | Medicinal rhubarb (reported constituent); commercial emodin, 98% purity (Xi'an Huacui Biotechnology) | SD rats; 10 Gy single-dose whole-abdominal 6-MV X-ray irradiation | Acute/early injury; ileum; therapeutic | 40 mg/kg/day by oral gavage q.d.; for 7 days; timing relative to irradiation NR | Preserved villus height, crypt depth, and mucosal/full-wall thickness; reduced mucosal edema/erosion and inflammatory infiltration | Reduced intestinal NO and MDA with increased SOD; descriptive/associative antioxidant/free-radical evidence without direct mechanistic perturbation; No direct *in vivo* pathway perturbation | Pathway-level association† | [61] |
| (-)-Epicatechin (EC) | Green tea (reported natural source) | Lgr5-EGFP-IRES-creERT2 C57BL/6 mice; 10 Gy single X-ray irradiation using RS 2000; irradiation field NR | Acute/early injury; small intestine; therapeutic | 5, 15, or 45 mg/kg by oral gavage once daily after irradiation; q.d.; post-IR | Reduced crypt apoptosis and oxidative injury; increased regenerative crypts, Ki67+ proliferation and Lgr5+ intestinal stem-cell activity; promoted epithelial regeneration | Enhanced Nrf2 nuclear accumulation with lower Keap1 and increased antioxidant-response targets, and increased β-catenin/Lgr5 signaling; *in vivo* evidence was expression/pathway-level without direct causal perturbation; No direct *in vivo* pathway perturbation | Pathway-level association† | [62] |
| (-)-Epigallocatechin-3-gallate (EGCG) | Green tea (reported constituent); purified EGCG purchased from Sigma-Aldrich | C57BL/6J mice, 6 weeks; 9 Gy single-dose total-body 60Co γ-irradiation | Acute/early injury; jejunum; prophylactic | 25 mg/kg p.o. once daily p.o.; q.d.; for 5 consecutive days before TBI, plus one dose 30 min after TBI | Preserved villus–crypt architecture and crypt regeneration; enhanced epithelial proliferation, crypt cells and Lgr5+ intestinal stem cells; partially restored radiation-disrupted gut-microbiota composition | 16S rRNA sequencing showed restoration of the Firmicutes/Bacteroidetes ratio and increased Bacteroides/Blautia/Turicibacter/Lactobacillus; microbiota changes correlated with intestinal phenotypes, but no FMT, antibiotic, germ-free, or targeted microbial perturbation was performed (associative microbiome evidence); no direct *in vivo* pathway perturbation.) → 16S rRNA, transfer/coadministration → Limit.: † | Pathway-level association† | [63] |
| (-)-Epigallocatechin-3-gallate (EGCG) | Green tea (reported natural source); purified EGCG purchased from Sigma-Aldrich | C57BL/6J mice; 9 Gy single-dose total-body 60Co γ-irradiation | Acute/early injury; small intestine; prophylactic | 12.5 or 25 mg/kg i.p.; q.d.; for 5 consecutive days before TBI plus one dose 30 min after TBI | Prolonged survival and reduced weight loss; preserved villus–crypt architecture and Lgr5+/Ki67+ regenerative cells; reduced intestinal apoptosis and oxidative DNA damage | Activated Nrf2/HO-1/Slc7A11/GPX4-associated antioxidant responses with reduced apoptosis/ferroptosis-related injury; *in vivo* evidence was expression-based, whereas Nrf2 dependence was functionally validated only in HIEC cells using ML385 No direct *in vivo* pathway perturbation | Pathway-level association† | [64] |
| Eutigoside C | Purified from leaves/twigs of *Eurya emarginata*; methanol extraction, fractionation/MPLC purification, spectroscopic identification | ICR mice: single 2 or 12 Gy whole-body 60Co γ-rays (3.1 Gy/min); male BALB/c mice: 9–13 Gy whole-body 60Co γ-rays for crypt assays | Acute/early injury; jejunum; prophylactic | 10, 50, or 100 mg/kg i.p. at 48, 24, and 1 h before irradiation (pre-IR; Methods); 100 mg/kg used for crypt-survival/DRF assays | Reduced jejunal crypt apoptosis; preserved villus height; increased surviving crypts (DRF 1.09) | Antioxidant/anti-inflammatory radioprotection proposed; hepatic SOD/CAT recovery indirect associative, but no intestinal pathway perturbation or causal validation was proposed | Pathway-level association† | [65] |
| *Evodia rutaecarpa* chloroform extract (evodiamine-rich; reported as evodiamine intervention) | Dried immature seeds of *Evodia rutaecarpa*; chloroform extract prepared after methanolic extraction | mice; 6 Gy single-dose whole-abdominal high-energy X-ray irradiation | Acute/early injury; ileum; prophylactic and therapeutic | Extract suspension equivalent to 2 g crude herb/mL by oral gavage twice daily; prophylaxis + treatment arm: 7 days before irradiation and 7 days from 12 h post-irradiation | Improved intestinal histology and delayed/reduced clinical-like enteritis signs; prophylaxis + treatment produced greater histologic protection than post-exposure treatment alone; increased intestinal DAO | Reduced intestinal IL-6-related mRNA and preserved DAO activity; descriptive/associative inflammatory/barrier evidence without targeted pathway perturbation or causal validation; No direct *in vivo* pathway perturbation | Pathway-level association† | [66] |
| Fructus sophorae-derived genistein (FSGen) | Fructus sophorae (dried ripe fruit of *Sophora japonica* L.); laboratory-prepared genistein, >99% purity reported | C57BL/6 mice; 7.5 Gy single-dose whole-body X-ray irradiation (reported dose rate 222.2 Gy/min; unit inconsistency flagged) | Acute/early injury; small intestine; prophylactic | 200 mg/kg s.c. at the posterior neck, single dose 24 h pre-IR | Preserved villus architecture and reduced edema/structural injury; reduced intestinal-cell apoptosis | Reduced intestinal IFN-γ expression and TUNEL-positive apoptosis; descriptive/associative anti-inflammatory and anti-apoptotic evidence without pathway perturbation; No direct *in vivo* pathway perturbation | Pathway-level association† | [67] |
| Fructus sophorae-derived genistein (FSGen) | Fructus sophorae [*Styphnolobium japonicum* (L.) Schott; syn. *Sophora japonica* L.]; extracted genistein, 98.82% purity with HPLC/MS/UV/IR/NMR characterization | C57BL/6 mice, 8 weeks; 7.5 Gy single-dose whole-body X-ray irradiation | Acute/early injury; unspecified; prophylactic | 200 mg/kg s.c., single dose 24 h pre-IR | Prolonged 30-day survival (50% vs 0% in vehicle-irradiated mice); preserved villus structure and reduced intestinal apoptosis/inflammation | RASSF1A/ERCC1 upregulation in intestinal tissue was associated with lower ↔ DNA-damage signals; siRNA knockdown in IEC-6 cells abolished FSGen radioprotection, providing genetic support at the cellular level but not *in vivo* causal validation Functional support was indirect/*in vitro* | Pathway-level association† | [68] |
| Genistein | Commercial genistein (Sigma); specific botanical source NR | BALB/c mice, 6 weeks, wk, bearing subcutaneous CT26 colon-cancer xenografts; 5 or 10 Gy single-dose abdominal irradiation with 6-MV high-energy photons, 3.8 Gy/min | Acute/early injury; jejunum; prophylactic | 200 mg/kg s.c. into the dorsal space, single dose 24 h pre-IR | Reduced jejunal crypt apoptosis; improved crypt survival, villus height and proliferative recovery; tumor weight after 10 Gy did not differ between irradiation alone and genistein + irradiation at day 7 (P=0.5) | Anti-apoptotic/pro-regenerative effects supported by TUNEL, Ki-67 and histomorphology; antioxidant, estrogen-receptor/Akt and G-CSF mechanisms were not directly tested (descriptive/associative mechanistic evidence); no direct *in vivo* pathway perturbation; → TUNEL/Ki-67 → Limit.: † | Pathway-level association† | [69] |
| Ginsenoside Rg3 (GRg3; Shenyi Capsule preparation) | *Panax ginseng* (reported active constituent); Shenyi Capsule, Jilin Yatai Pharmaceutical | Wistar rats; 21.5 Gy single-dose local pelvic/rectal-region 6-MV X-ray irradiation | Acute/early injury; rectum; therapeutic | 20, 40, or 80 mg/kg by oral gavage once daily; started on day 8 after irradiation and continued for 14 days | High-dose GRg3 improved body-weight recovery and rectal histopathology; reduced mucosal ulceration/inflammatory injury compared with vs untreated irradiated rats | Reduced TNF-α and IKK-β/p-IκB-α/nuclear NF-κB p50/Caspase-8-related changes with increased IL-4/IL-10 and cytoplasmic p50 retention; pathway-level association without direct pathway perturbation → cytokine assay → Limit.: † | Pathway-level association† | [70] |
| Ginsenoside Rk1 | Ginseng-derived rare ginsenoside (reported source context); commercial Rk1 from Wekeqi Biotech | SD rats, 6 weeks; 14 Gy single-dose total-abdominal irradiation by linear accelerator | Acute/early injury; unspecified; therapeutic | 10 mg/kg by oral gavage once daily for 5 consecutive days after irradiation | Improved 14-day survival; reduced weight loss/diarrhea and intestinal histopathologic injury; reduced apoptosis and preserved crypt-cell proliferation | RNA-seq and protein analyses implicated PI3K/AKT/mTOR suppression; IGF-1 rescue in IEC-6 cells reversed Rk1-associated proliferative/anti-apoptotic effects and pathway changes, supporting functional pathway involvement *in vitro* | Pathway-level association† | [71] |
| Glycyrrhizin (GL) | *Glycyrrhiza glabra* (reported natural source); commercial glycyrrhizin, purity ≥98% | C57BL/6 mice, 8 weeks; 6.5 Gy single-dose abdominal X-ray irradiation | Acute/early injury; jejunum; prophylactic | 5, 10, or 20 mg/kg/day i.p.; first dose 2 h before irradiation, then the same dose within 2 h after irradiation for 3 consecutive days; assessed at day 3.5 | Preserved jejunal villus/crypt architecture; reduced weight loss; improved D-xylose intestinal absorption | Reduced inflammatory cytokines and HMGB1/TLR4 expression; pathway-level association based on ELISA, western blot and immunofluorescence without direct HMGB1/TLR4 perturbation; No direct *in vivo* pathway perturbation | Pathway-level association† | [72] |
| Hesperidin; Quercetin | Commercial compounds from Sigma-Aldrich; specific botanical source of test materials NR | rats, strain NR (n=24; 6/group); 10 Gy single-dose abdominopelvic 6-MV irradiation | Acute/early injury; colon; prophylactic (assessed 24 h after irradiation) | Hesperidin 200 mg/kg/day or quercetin 50 mg/kg/day for 15 days before irradiation; route NR | No histopathologic or functional injury endpoint was assessed; evidence was limited to colonic biochemical markers, with stronger marker changes for quercetin | Both lowered TNF-α and increased IL-10; quercetin significantly reduced caspase-3/8/9, whereas hesperidin did not significantly reduce caspases and increased HSP70 | Pathway-level association† | [73] |
| *Hibiscus sabdariffa* polysaccharides (TP; purified HSP1) | *Hibiscus sabdariffa* L. flower buds; HSP1 purified from total polysaccharides | C57BL/6J mice; 6 Gy single-dose abdominal γ-irradiation using a Gammacell-40 137Cs source, 0.88 Gy/min | Acute/early injury; colon; prophylactic | TP 100 mg/kg/day or HSP1 10/100 mg/kg/day by oral gavage for 7 days before irradiation; then every other day q.o.d. | Improved body-weight recovery; reduced colonic histologic/barrier injury and fecal albumin; restored mucus/tight-junction integrity | Restored Nrf2/HO-1 expression/redox status and reduced NOD1/TLR2/TLR4–p65-associated inflammatory markers; 16S profiling showed microbiota remodeling. pathway-level remodeling | Pathway-level association† | [74] |
| Icariside II (ICS II) | *Epimedium* (reported natural source); commercial ICS II, HPLC ≥98% | SD rats; 20 Gy single 6-MV irradiation using a Siemens medical linear accelerator; irradiation field NR | Acute/early injury; colon; therapeutic | 4.5 mg/kg/day by oral gavage for 4 weeks, started 24 h after irradiation | Improved body weight/colon length and DAI; reduced intestinal permeability; restored colonic mucosal/crypt architecture and barrier integrity | Restored mitochondrial function and increased PINK1/Parkin/LC3-associated mitophagy markers *in vivo*; Mdivi-1 perturbation in irradiated Caco-2 cells supported functional involvement of mitophagy, without genetic validation; No direct *in vivo* pathway perturbation | Pathway-level association† | [75] |
| Isoliquiritigenin (ISO) | Licorice and other traditional Chinese medicines (reported source); commercial ISO ≥98% | C57BL/6 mice, bearing subcutaneous Lewis tumors; 4 Gy single-dose local tumor-site X-ray irradiation | Acute/early injury; colon; therapeutic | 40 mg/kg i.p.; (0.2 mL/day) once daily mL/d) q.d.; for 7 consecutive days, initiated the day after irradiation | Improved colonic histopathology and epithelial barrier markers; reduced colonic apoptosis; restored Lgr5/Axin2/Ki67-associated stem-cell proliferation | Reduced TNF-α/TNFR and caspase/apoptosis-related changes; docking suggested a potential TNF-α interaction only, with no direct-binding or pathway-perturbation validation Indirect/contextual evidence:: the intestinal tissue was not directly irradiated. → molecular docking, cytokine assay → Limit.: § | Indirect/contextual§ | [76] |
| Licochalcone D (LCD) | Licorice (reported natural source); commercial LCD (TargetMol), purity NR | C57BL/6 mice; whole-abdominal irradiation, 12 Gy or 14 Gy; radiation source/dose rate NR | Mixed; small intestine; prophylactic | 20 mg/kg i.p.; for 2 days before WAI and again 1 h before WAI | At 12 Gy, reduced weight loss, preserved crypt–villus architecture and crypt regeneration, and reduced day 90 submucosal fibrosis; no survival benefit after 14 Gy (all mice died within 7 days) | CQ and the SIRT3 inhibitor 3-TYP attenuated or abolished intestinal protection *in vivo*; SIRT3 siRNA abolished LCD-induced mitophagy and cellular radioprotection, supporting SIRT3-dependent mitophagy | Causal/functional | [77] |
| Liriodendrin | *Sargentodoxa cuneata*; extracted compound, purity >97% by HPLC; structure confirmed by NMR | C57BL/6J mice; 8 Gy single 60Co irradiation (GWXJ80 60Co teletherapy machine; reported dose rate 39.22 Gy/min; irradiation field NR) | Acute/early injury; small intestine; prophylactic | 100 mg/kg p.o.; started 3 days before irradiation and continued for 7 days after irradiation; dosing frequency NR | Reduced small-intestinal histopathologic injury; decreased neutrophil/inflammatory infiltration and pro-inflammatory cytokines; reduced intestinal apoptosis | Lower Cer/Cer1P/S1P with reduced p-NF-κB and Bax/caspase-3 and increased Bcl-2; sphingolipid and NF-κB/apoptosis pathway-level associative evidence without causal perturbation; No direct *in vivo* pathway perturbation | Pathway-level association† | [78] |
| Liriodendron (authors’ terminology; C34H46O18 material) | Fibraureae Caulis (reported source); commercial material from Tianjin Furui Xiang Technology Co., Ltd.; purity/chemical authentication NR | C57/BL mice; 10 Gy single-dose localized pelvic X-ray irradiation | Late/chronic remodeling; rectum; therapeutic | 100 mg/kg i.g. or enema, initiated 24 h after irradiation; followed for 8 weeks; exact treatment frequency NR | Gavage improved 8-week survival and reduced fecal occult blood/rectal histopathology; reduced rectal fibrosis and α-SMA; enema was generally less effective | Transcriptomics/GSEA/qPCR indicated reduced collagen/TGF-β/Smad-related signaling with lower ROS/apoptosis and fibrosis markers; pathway-level associative evidence without causal perturbation; No direct *in vivo* pathway perturbation.markers → qPCR, RNA-seq/transcriptomics, GSEA → Limit.: † | Pathway-level association† | [79] |
| *Lycium barbarum* polysaccharide (LBP) | *Lycium barbarum* (reported source); commercial LBP, purity >950 mg/g, batch KZ20160812 | Kunming mice, equally represented; 6 Gy single-dose abdominal 6-MV X-ray irradiation | Acute/early injury; small intestine; therapeutic | 200, 400, or 800 mg/kg by oral gavage once daily for 7 days, initiated 2 h after irradiation (0.5 mL/dose) | Increased villus length and crypt number; improved isolated IEC viability and reduced apoptosis; dose-dependently improved antioxidant indices | Increased Bcl-2 and reduced Bax with higher SOD/lower MDA; descriptive/associative apoptosis and oxidative-stress evidence without direct pathway perturbation → redox assays → Limit.: † | Pathway-level association† | [80] |
| *Orychophragmus violaceus* extract OVS-2 (alkaloid extract) | *Orychophragmus violaceus* seeds; 70% ethanol extraction followed by D001 cation-exchange resin purification | C57BL/6 mice (sex/age/weight NR); 15 Gy single-dose targeted abdominal 60Co γ-irradiation; non-abdominal regions shielded | Acute/early injury; small intestine; prophylactic | 100 mg/kg p.o.; at 24 h before, 0.5 h after, and 24 and 48 h after irradiation (Methods schedule) | Improved 30-day survival (100% vs 40% in untreated irradiated mice); reduced weight loss; preserved villus/crypt morphology | 16S rRNA profiling showed microbiota-community shifts associated with intestinal protection; no FMT, antibiotic, germ-free, or targeted microbial/metabolite validation (associative microbiome evidence); no direct *in vivo* pathway perturbation.) → 16S rRNA, transfer/coadministration → Limit.: † | Pathway-level association† | [81] |
| p-Coumaric acid (CA) | NR (commercial p-coumaric acid; specific botanical source and purity not reported) | C57BL/6 mice; 4 Gy single-dose total-body X-ray irradiation | Acute/early injury; jejunum; prophylactic | 50, 100, or 200 mg/kg i.g.; initiated 30 min before irradiation and continued for 3 days after irradiation; dosing frequency NR | Improved jejunal morphology, with greatest structural benefit at 200 mg/kg; reduced epithelial apoptosis and oxidative/inflammatory injury | Reduced CASP-1/NLRP3 transcripts and AIM2 transcript in selected dose/time comparisons; corresponding pyroptosis-related protein differences were not significant. associative evidence only No direct *in vivo* pathway perturbation.significant → expression/phenotype assays → Limit.: † | Pathway-level association† | [82] |
| Perillaldehyde (PAH) | Perilla plants/*Perilla frutescens* (reported natural source); commercial PAH (AbMole), purity NR | C57BL/6J mice; 13 Gy single-dose total-abdominal X-ray irradiation | Acute/early injury; unspecified; prophylactic | 100 mg/kg/day by oral gavage for 7 consecutive days before irradiation | Prolonged mean survival (5.1 to 6.5 days; all mice died by day 12); preserved crypt–villus architecture/regeneration | Activated Nrf2/Slc7A11/GPX4 and suppressed ferroptosis-related lipid peroxidation; ML385 partially blocked radioprotection in irradiated HIEC-6 cells and intestinal organoids (functional pharmacologic evidence) | Pathway-level association† | [83] |
| Puerarin | *Pueraria lobata* (Gegen) rhizome (reported natural source); commercial puerarin injection | SD rats, 7 weeks; 12 Gy single lower-abdominal 6-MV X-ray irradiation | Acute/early injury; jejunum; therapeutic | 15 mg/kg/day by tail-vein injection, initiated after irradiation and continued post-IR for 7 consecutive days | Preserved villus/crypt architecture and crypt regeneration; increased epithelial proliferative recovery; restored tight-junction proteins and reduced oxidative injury | Increased Ki-67 and claudin-1/ZO-1 with lower MDA and higher SOD; descriptive/associative barrier-regeneration and antioxidant evidence without direct pathway perturbation → TUNEL/Ki-67, redox assays → Limit.: † | Pathway-level association† | [84] |
| Puffed ginseng extract (PGE) | *Panax ginseng* C.A.Mey.; explosively puffed, 70% ethanol extract; HPLC ginsenoside profiling | ICR mice, 6 weeks; 8 Gy single 137Cs irradiation; field/geometry NR | Acute/early injury; multiple sites; prophylactic | 200 mg/kg by oral gavage once daily for 4 weeks before irradiation (pre-IR) | Improved colorectal architecture and histologic severity; reduced epithelial apoptosis; attenuated oxidative/inflammatory injury | Reduced p53-associated DNA-damage responses, NF-κB/MAPK-related signaling and intrinsic/extrinsic apoptotic markers, with lower oxidative-stress markers; pathway-level association based on expression/phosphorylation assays without direct perturbation; No direct *in vivo* pathway perturbation | Pathway-level association† | [85] |
| *Pyrrosia petiolosa* aqueous extract | *Pyrrosia petiolosa* (Christ) Ching; aqueous decoction, botanically authenticated (voucher SZ 162295); LC–MS profiled | BALB/c mice; 7.5 Gy single-dose abdominal X-ray irradiation, 1.14 Gy/min | Acute/early injury; jejunum; unspecified | 200 μL P. petiolosa solution (300 mg/mL) by oral gavage daily; timing relative to irradiation NR | Preserved villus architecture/length and crypt Ki67+ proliferation; reduced inflammatory/oxidative injury; improved WBC/lymphocyte recovery | Associated with anti-inflammatory/antioxidant effects plus microbiome–metabolome remodeling; 16S, untargeted metabolomics and correlation/enrichment analyses provided associative multi-omic evidence without causal microbiota/metabolite perturbation; No direct *in vivo* pathway perturbation | Pathway-level association† | [86] |
| Quercetin | Common constituent of Baitouweng decoction, Gegen Qinlian decoction, and Shaoyao decoction; HPLC-confirmed in all three formulas | C57BL/6J mice; 6 Gy single 60Co irradiation; body region NR | Acute/early injury; colon; prophylactic | 50 or 100 mg/kg i.g.; once, 30 min before irradiation; pre-IR | Improved colonic histopathology, particularly at 50 mg/kg; reduced pro-inflammatory cytokines and increased IL-10; altered gut microbial communities | Network pharmacology/docking suggested quercetin and IL10/STAT1/IL1B/IL1A as shared candidates; cytokine changes and 16S microbiota shifts provided bioinformatic/associative evidence without direct target or microbiota perturbation; No direct *in vivo* pathway perturbation | Pathway-level association† | [87] |
| Resveratrol | Commercial resveratrol (Sigma-Aldrich); specific botanical source NR | C57BL/6 mice; 12 Gy single-dose abdominal high-energy X-ray irradiation | Acute/early injury; small intestine; prophylactic | 40 mg/kg/day i.p.; started 3 days before irradiation and continued until death/euthanasia; tissue cohort collected on day 3 post-IR | Improved survival; preserved villus–crypt architecture and Ki67+ + Ki67+ regeneration; reduced intestinal apoptosis | SIRT1 involvement was functionally supported by Ex527, which attenuated survival/histologic and molecular benefits; IEC-6 experiments linked SIRT1 to FOXO3a deacetylation/nuclear localization and p53-associated apoptosis. | Causal/functional | [88] |
| Resveratrol | Commercial resveratrol (NeoCell); specific botanical source NR | albino rats, strain NR; 5 Gy single 137Cs γ-irradiation; field/geometry NR | Persistent/subacute injury; unspecified; therapeutic (assessed 4 weeks after irradiation) | 20 mg/kg/day by oral gavage for 3 weeks, started 1 week after irradiation; tissues collected 24 h after the last dose | Reduced villus necrosis/atrophy and inflammatory infiltration; improved intestinal histopathology and redox status | Reduced intestinal TNF-α/NF-κB/IL-1β and PI3K/Akt/mTOR protein expression; pathway-level association without direct pathway perturbation or rescuerescue → cytokine assay → Limit.: † | Pathway-level association† | [89] |
| Resveratrol | NR (commercial resveratrol, 97% purity; botanical source of the test material not reported) | C57BL/6 mice; 7.2 Gy single-dose total-body 137Cs γ-irradiation | Acute/early injury; unspecified; prophylactic | 50, 100, or 200 mg/kg/day by oral gavage for 7 days before irradiation and 14 days after irradiation | Improved intestinal histopathology, with the 100 mg/kg group showing the greatest protection; facilitated post-irradiation body-weight recovery; reduced intestinal IL-1β expression | Increased SIRT1 and decreased NLRP3/IL-1β in spleen/thymus, with reduced intestinal IL-1β; associative/pathway-level evidence only, without direct pathway perturbation or intestinal SIRT1/NLRP3 validation → cytokine assay → Limit.: † | Pathway-level association† | [90] |
| Rheinic acid (RA) | Rhubarb (reported natural source); commercial RA from Zelang (Nanjing) | SD rats; 10 Gy single-dose abdominal X-ray irradiation | Acute/early injury; multiple sites; therapeutic | 90, 180, or 360 μg/kg/d by oral gavage q.d.; for 7 days; timing relative to irradiation NR | Reduced DAI/weight loss and bloody stool; dose-dependently attenuated ileal/colonic mucosal injury; reduced inflammatory and oxidative injury | Upregulated PPAR-γ and reduced NF-κB and p38/JNK phosphorylation with lower apoptotic markers; pathway-level association only, without direct pathway perturbation → expression/phenotype assays → Limit.: † | Pathway-level association† | [91] |
| *Rheum tanguticum* polysaccharide fraction 1 (RTP1) | *Rheum tanguticum*; water-extracted/ethanol-precipitated polysaccharide purified by DEAE-52 and Sephacryl S-200 chromatography | SD rats; 10 Gy single-dose whole-abdominal 6-MV X-ray irradiation | Acute/early injury; small intestine; prophylactic | 200, 400, or 800 mg/kg by oral gavage daily for 7 days; irradiation performed on day 7 after the pretreatment course | Preserved villus architecture/height and crypt depth, especially at higher doses; reduced intestinal-barrier injury markers (DAO, D-lactate, endotoxin) | Increased SOD/GSH and reduced MDA with improved barrier-related biomarkers; descriptive/associative antioxidant and barrier evidence without direct mechanistic perturbation; No direct *in vivo* pathway perturbation | Pathway-level association† | [92] |
| *Rheum tanguticum* polysaccharides (RTP) | *Rheum tanguticum* Maxim. ex Regel rhizomes; polysaccharide preparation from the Air Force Military Medical University laboratory | SD rats; 12 Gy single-dose total-abdominal 6-MV X-ray irradiation | Acute/early injury; jejunum; prophylactic | 500 mg/kg by oral gavage once daily from 1 day before irradiation through day 7 after irradiation | Improved survival/physiologic status and jejunal histopathology; reduced oxidative injury, apoptosis and inflammatory responses; preserved proliferative/regenerative markers | Enhanced Nrf2 nuclear translocation and Nrf2/HO-1 expression with lower ROS, NF-κB/inflammatory mediators and caspase-3; pathway-level evidence based on temporal/expression analyses without direct Nrf2 perturbation; No direct *in vivo* pathway perturbation | Pathway-level association† | [93] |
| Rosavin | *Rhodiola rosea* L. (reported natural source); commercial rosavin (Sigma), purity NR | SD rats, 7 weeks, wk, ~; 14 Gy single-dose whole-abdominal irradiation | Acute/early injury; unspecified; therapeutic | 200 mg/kg/day by oral gavage (2 mL) for 7 days; first dose immediately after irradiation (days 0–6 post-IR) | Improved 14-day survival; restored villus/crypt architecture and epithelial thickness; improved intestinal permeability/absorption | Reduced TNF-α/IL-1β/MPO and MDA with increased IL-10/Tregs and SOD/CAT/GPx; associative evidence without direct pathway perturbation → redox assays, cytokine assay → Limit.: † | Pathway-level association† | [94] |
| Sanguinarine (SAN) | Papaveraceae plants (reported general natural source); commercial sanguinarine chloride hydrate, 98% purity | C57BL/6J mice; 10 Gy single-dose whole-abdominal X-ray irradiation | Acute/early injury; small intestine; prophylactic | 2.5 mg/kg i.p.; during the 3-day pretreatment period, including administration ~30 min before irradiation; pre-IR | Preserved crypt–villus architecture and epithelial regeneration; reduced intestinal apoptosis and inflammatory injury; partially restored radiation-disrupted gut microbiota | Downregulated HMGB1/TLR4/MyD88/NF-κB with altered cytokines and microbiota/SCFAs; pathway-level associative evidence only, without direct pathway or microbiota perturbation; No direct *in vivo* pathway perturbation | Pathway-level association† | [95] |
| Tea polyphenols (TP) | Tea (specific botanical species NR); TP mixture characterized by UPLC-Q-TOF/MS | C57BL/6J mice; 60Co γ-irradiation: 10 Gy TBI survival model and 15 Gy total-abdominal irradiation intestinal-injury model | Acute/early injury; unspecified; prophylactic | Oral gavage for 5 days before irradiation; survival model: 110, 220, or 440 mg/kg/day | High-dose TP prolonged survival; reduced colon shortening and intestinal mucosal injury; lowered inflammatory/oxidative injury | *In vivo* Nrf2/HO-1/GPX4/HSP90 changes and microbiota associations were non-causal; complementary IEC-6 studies showed EGCG–HSP90 binding by BLI and HSP90 RNAi reduced ferroptosis, providing target/genetic support for HSP90 biology but not proving that TP protection requires HSP90 → expression/phenotype assays → Limit.: † | Pathway-level association† | [96] |
| Thymoquinone (TQ) | *Nigella sativa* seed oil (reported natural source); test-compound purity NR | C57BL/6 mice, sex/age/weight NR; single 7.5, 9.0, or 11.0 Gy total-body 137Cs γ-irradiation; 9.0 Gy used for tissue studies | Acute/early injury; unspecified; prophylactic | Single oral gavage 1 days before irradiation; 10 mg/kg used for tissue studies | Improved survival across TBI models; preserved crypt–villus architecture and crypt/stem-cell regeneration; reduced intestinal apoptosis and DNA-damage markers | Reduced intestinal p53, γH2AX, Bax and caspase-8/-9/-3 with fewer TUNEL-positive cells; descriptive/pathway-level associative evidence only, without direct p53/caspase perturbation; No direct *in vivo* pathway perturbation | Pathway-level association† | [97] |
| WGX50 | *Zanthoxylum bungeanum* Maxim. (reported natural source); supplied WGX50 powder, purity NR | C57BL/6 mice, 6 weeks, wk, ~; 13 Gy single-dose abdominal X-ray irradiation | Acute/early injury; multiple sites; prophylactic | 28 mg/kg/day by oral gavage for 3 weeks before irradiation; intestinal tissues collected 7 days post-IR | Reduced weight loss and intestinal shortening; preserved villus/crypt architecture and crypt survival; protected goblet-cell/barrier features | SPR confirmed direct WGX50–EGFR binding (KD 5.86 μM); EGFR activation and SLC7A11/GPX4 upregulation accompanied ferroptosis suppression. | Pathway-level association† | [98] |

Notes: Evidence categories are causal/functional (pathway-dependency perturbation in the relevant irradiated model), pathway-level association (target/pathway readouts without a dependency test), phenotypic/descriptive (outcomes without pathway validation), and indirect/contextual (nonirradiated or non-RE models). *In vitro* functional validation, where cited as supporting evidence, does not establish *in vivo* causality.

Common limitations: † no mechanism-dependency perturbation in the original article; ‡ *in vitro* functional validation is not equivalent to *in vivo* causality; § nonirradiated/non-RE models provide contextual rather than radioprotective evidence. Expression changes, omics, network pharmacology, and docking are hypothesis-generating unless functionally perturbed in the relevant irradiated model.

Abbreviations: DEG, differentially expressed gene; IR, irradiation; NR, not reported; post-IR, after irradiation; pre-IR, before irradiation; h, hour; d, day; wk, week; mo, month; i.g., intragastric gavage; i.p., intraperitoneal injection; i.v., intravenous injection; p.o., oral administration; s.c., subcutaneous injection; q.d., once daily; q.o.d., every other day; b.i.d., twice daily; t.i.d., three times daily; q.n., nightly; SD, Sprague–Dawley; HPLC, high-performance liquid chromatography; LC–MS, liquid chromatography–mass spectrometry. Symbols: ↑, increase/upregulation; ↓, decrease/downregulation; →, evidence chain.

**Table S4.** *In vitro* evidence for herbal extracts and bioactive compounds in radiation-induced intestinal injury

| **Extract/compound** | **Source** | ***In vitro* model** | **Irradiation conditions** | **Dose; timing** | **Core outcomes** | **Mechanism/evidence (target → validation → limitation)** | **Evidence category** | **References** |
| --- | --- | --- | --- | --- | --- | --- | --- | --- |
| 6-Shogaol | Ginger oleoresin; purified by preparative HPLC (98.4% purity) | Rat IEC-6 intestinal epithelial cells (CRL-1592; passage 28, used ≤32) | Single 4 Gy γ-irradiation | 20 μM 6-shogaol for 4 h before irradiation (pre-IR); outcomes assessed at 24–72 h, assay-dependent | Preserved transepithelial electrical resistance after irradiation; reduced post-IR radiation-associated intracellular ROS | Antioxidant/barrier protection supported by ROS and TEER assays only; associative evidence without targeted pathway perturbation or causal validation → TEER/permeability → Limit.: † | Pathway-level association† | [41] |
| Aloperine (ALO) | *Sophora alopecuroides* L. (reported natural source); commercial ALO, purity ≥98% | Rat IEC-6 small-intestinal crypt epithelial cells | 0–8 Gy screened; single 6 Gy irradiation used for main mechanistic assays; radiation source/dose rate NR | 3, 10, or 30 μM for 2 h before 6 Gy irradiation; cells assessed after ~24 h; 30 μM used for most mechanistic perturbation experiments | Improved viability; reduced Fe2+ accumulation and lipid peroxidation; restored GPX4/SLC7A11 and shifted glucose metabolism toward HBP/UDP-GlcNAc | Docking/CETSA supported ALO–TAS2R138 target engagement; TAS2R138 sgRNA knockout weakened ALO-driven metabolic reprogramming and anti-ferroptotic effects, while GFPT1 knockdown and GAPDH overexpression supported the PLCβ/GAPDH–HBP mechanism (genetic/causal and functional evidence) | Causal/functional (*in vitro*)‡ | [43] |
| Baicalein | *Scutellaria baicalensis* dry root (reported natural source); commercial baicalein (Sigma-Aldrich), purity NR | Human umbilical vein endothelial cells (HUVECs) | Single 10 Gy 137Cs γ-irradiation; 3.81 Gy/min | Baicalein concentration NR; added within 1 h after irradiation; post-IR | Reduced radiation-induced P-selectin, ICAM-1, and VCAM-1 mRNA expression | Supports an endothelial-adhesion/activation mechanism; associative evidence only, without functional or genetic perturbation → expression/phenotype assays → Limit.: † | Pathway-level association† | [48] |
| Berberine (BBR; drug-containing serum) | *Coptis* and *Berberis* spp. (reported natural sources); serum obtained from berberine-treated rats | Rat IEC-6 intestinal epithelial cells (ATCC CRL-1592) | Single 12 Gy 60Co γ-irradiation over 3 min; dose rate NR | 10% (v/v; drug-containing serum: medium = 1:10) serum from berberine-treated rats added after irradiation; post-IR | Reduced Bax, caspase-3, and cytochrome-c mRNA; increased Bcl-2 mRNA versus irradiated model | Apoptosis-related gene-expression changes only; associative evidence without direct apoptosis measurement, pathway perturbation, or causal validation → expression/phenotype assays → Limit.: † | Pathway-level association† | [51] |
| *Centella asiatica* preparation (CA) | *Centella asiatica*; commercial USP preparation | Human HUVECs; differentiated human Caco-2 monolayers as an intestinal epithelial barrier model | HUVECs: 10 Gy 137Cs γ-rays, 3.25 Gy/min; Caco-2 monolayers: 15 Gy | post-irradiation CA; 5–50 µg/mL tested in HUVEC viability assays; 24–48 h depending on assay | Improved HUVEC viability, senescence, and angiogenic function; CA-conditioned medium restored Caco-2 TEER, reduced FITC-dextran flux, strengthened cell adhesion, and restored ZO1/DSG2 | CA increased endothelial EGF production/secretion; recombinant EGF recapitulated barrier rescue, whereas EGF-neutralizing antibody abolished CA-conditioned-medium rescue and ZO1/DSG2 restoration (functional/mechanistic evidence | Causal/functional (*in vitro*)‡ | [55] |
| Crocetin | *Crocus sativus* L. flower stigmas (reported natural source); test-material purity NR | Human intestinal epithelial cells (HIECs), passages 4–8 | X-rays; 2 Gy/min; total irradiation dose NR | 0, 5, 10, or 20 μM for 24 h after irradiation | Increased cell viability; reduced pyroptosis, LDH release and IL-1β/IL-18; reduced caspase-1/GSDMD-N activation | Crocetin reduced KAT2A; knockdown, NLRP3-K21 mutation and NLRP3-overexpression rescue established a causal KAT2A–NLRP3 succinylation/pyroptosis axis, although direct proof that crocetin protection requires KAT2A was not provided (genetic/causal downstream mechanism); no direct pathway perturbation. → overexpression/knockdown → Limit.: † | Pathway-level association† | [56] |
| Crocetin | *Crocus sativus* L. stigma (reported natural source); commercial crocetin (MP Biomedicals), purity NR | Rat IEC-6 intestinal epithelial cells | Single 10 Gy irradiation using a Siemens PRIMUS linear accelerator; 300 cGy/min | 0, 0.1, 1, 10, or 100 μM added after irradiation for 24 h; 10 μM selected for subsequent experiments | Increased viability; reduced apoptosis; improved antioxidant-enzyme activity and reduced lipid peroxidation/pro-inflammatory cytokines | Antioxidant, anti-inflammatory and anti-apoptotic effects were supported by SOD/CAT/GPx, MDA/MPO, cytokine and apoptosis measurements; associative evidence without pathway perturbation → redox assays, cytokine assay → Limit.: † | Pathway-level association† | [99] |
| Cynaroside (luteoloside; luteolin-7-O-glucoside) | *Lonicera* species (reported natural source); commercial purified cynaroside >98% | Human NCM460 colonic epithelial cells | Single 8 Gy X-ray irradiation using a Varian linear accelerator (2 Gy/min) | 0–20 μg/mL screened; 10 μg/mL selected; detailed viability/colony/γH2AX protocols used 4 h pretreatment before irradiation | Increased viability/clonogenic survival; reduced γH2AX DNA damage; downregulated Dnm2 | Dnm2 downregulation accompanied radioprotection; no cell-specific Dnm2 inhibitor/rescue/genetic perturbation was reported → pharmacologic inhibitor/activator → Limit.: † | Pathway-level association† | [58] |
| Defined core-component mixture: puerarin, baicalein, berberine, and glycyrrhetinic acid (molar ratio 254:186:14:1) | Commercially purchased purified compounds; specific botanical source NR | HIEC-6 normal human intestinal epithelial cells | Single 4 Gy X-ray exposure (6-MV linear accelerator; 400 MU/min) | Low-, medium-, or high-concentration mixture added immediately post-IR for 24 h; numerical concentrations NR | Reduced pro-inflammatory cytokines and TUNEL positivity; restored IL-10 and Ki67 after irradiation | Non-additivity with an NF-κB inhibitor, a Caspase-9/-3 inhibitor, or a Wnt/β-catenin agonist, together with p65, caspase, β-catenin nuclear accumulation, MYC, and CCND1 analyses, supports pharmacologic functional involvement of all three pathways; No genetic or target-engagement validation; No direct pathway perturbation. → pharmacologic inhibitor/activator → Limit.: † | Pathway-level association† | [21] |
| Ecliptasaponin II (Ecl2) | Ecliptae Herba (reported source); purified Ecl2 ≥98% | Human umbilical vein endothelial cells (HUVECs; PromoCell) | Single 10 Gy 6-MV X-ray irradiation using a Varian Clinac iX linear accelerator (400 cGy/min; source-to-skin distance 100 cm) | 1, 3, or 10 μM Ecl2 for 2 h before irradiation; pre-IR; assessed 24 h after irradiation | Improved cell viability and reduced LDH release, ROS, and apoptosis; preserved mitochondrial membrane potential and reduced mitochondrial apoptotic signaling | Docking suggested potential PPARγ interaction; Ecl2 increased PPARγ nuclear/transcriptional activity, while GW9662 abolished/reversed anti-apoptotic and Bax/Bcl2-Cyt-C-Caspase-3/PARP effects, supporting functional PPARγ involvement (pharmacological validation | Causal/functional (*in vitro*)‡ | [60] |
| (-)-Epicatechin (EC) | Green tea (reported natural source) | Human NCM460 intestinal epithelial cells; mouse small-intestinal crypt organoids/ex vivo “minigut” model | NCM460: single 8 Gy γ-irradiation; organoid experiments included crypts isolated after 10 Gy *in vivo* irradiation and a separate 8 Gy regeneration assay | NCM460: EC added after irradiation for 24 h; 50–400 μM tested, with 100 μM used in key mechanistic analyses. Organoid experiments included crypts from mice receiving 15 mg/kg EC | Enhanced NCM460 antioxidant/regenerative signaling; improved organoid survival, budding/proliferation and Lgr5+ stem-cell representation after irradiation | Nrf2/Keap1–HO1/NQO1 changes were primarily expression-based; TCF/LEF reporter activation plus attenuation of EC-induced Lgr5/CyclinD1 by the Wnt/β-catenin inhibitor PUN74654 supported functional Wnt-pathway involvement | Causal/functional (*in vitro*)‡ | [62] |
| (-)-Epigallocatechin-3-gallate (EGCG) | Green tea (reported natural source); purified EGCG purchased from Sigma-Aldrich | Human intestinal epithelial HIEC cells | X-ray irradiation, 1.15 Gy/min; 2–8 Gy depending on assay (4–8 Gy for most mechanistic analyses) | 2 μM EGCG for 30 min before irradiation; pre-IR; drug-containing medium replaced immediately after irradiation | Increased viability/clonogenic survival; reduced γH2AX/8-OHdG DNA damage, ROS, apoptosis and lipid peroxidation/ferroptosis | EGCG activated Nrf2 with HO-1/Slc7A11/GPX4 upregulation; ML385 nearly abolished clonogenic protection and partially reversed anti-apoptotic/anti-ferroptotic effects, supporting functional Nrf2 involvement (pharmacological perturbation | Causal/functional (*in vitro*)‡ | [64] |
| Fructus sophorae-derived genistein (FSGen) | Fructus sophorae (*Sophora japonica* L.); laboratory-prepared genistein, >99% purity reported | Rat IEC-6 small-intestinal epithelial cells | Single 6 Gy X-ray irradiation (444.4 cGy/min) | 1, 5, 10, or 20 μM for 24 h before irradiation; pre-IR; 5 μM selected for apoptosis/DNA-damage experiments | Increased post-irradiation viability; reduced apoptosis and comet-assay DNA damage | Phenotypic radioprotection only; no defined molecular target/pathway was directly tested (descriptive evidence); no direct pathway perturbation. → expression/phenotype assays → Limit.: † | Phenotypic/descriptive† | [67] |
| Fructus sophorae-derived genistein (FSGen) | Fructus sophorae [*Styphnolobium japonicum* (L.) Schott]; extracted genistein, 98.82% purity with HPLC/MS/UV/IR/NMR characterization | Rat IEC-6 small-intestinal epithelial cells | Single 6 Gy X-ray irradiation (RS2000; 444.4 cGy/min); 12 Gy used in dose-screening assays | 0–16 μM screened; 4 μM selected and applied for 24 h before 6 Gy irradiation; assay-specific follow-up at 24–72 h | Increased viability; reduced apoptosis, ROS, senescence and DNA strand-break/γH2AX damage | FSGen increased RASSF1A/ERCC1 expression; knockdown of RASSF1A and/or /or ERCC1 eliminated radioprotection, providing genetic loss-of-function evidence for causal involvement in IEC-6 cells *In vitro* functional validation | Causal/functional (*in vitro*)‡ | [68] |
| Ginsenoside Rg1 | NR; commercial ginsenoside Rg1 purchased from Shanghai Yifei Biotechnology | Rat IEC-6 intestinal epithelial cells | Single 4 Gy 60Co γ-irradiation; source-to-cell distance 4 m; dose rate NR | 10 μM Rg1; exact timing relative to irradiation NR; 10 μM LY294002 used in the mechanistic group | Increased cell viability and reduced apoptosis at 12–24 h; protective differences were largely no longer evident by 36 h | Rg1 increased p-AKT/Bcl-2 and reduced Bax/Bax: Bcl-2; PI3K inhibitor LY294002 attenuated the viability/anti-apoptotic and molecular effects, supporting functional PI3K/AKT involvement (pharmacological perturbation | Causal/functional (*in vitro*)‡ | [100] |
| Ginsenoside Rk1 | Ginseng-derived rare ginsenoside (reported source context); commercial Rk1 from Wekeqi Biotech | Rat IEC-6 intestinal crypt epithelial cells (ATCC; <20 passages) | Single 10 Gy irradiation using a Siemens PRIMUS linear accelerator (300 cGy/min) | 0.4, 2, 10, or 50 μM Rk1 added after irradiation; post-IR; 10 μM selected for mechanistic studies | Increased cell viability; reduced ROS and apoptosis; maintained proliferation-associated responses after irradiation | Rk1 suppressed radiation-induced p-PI3K/p-AKT/p-mTOR activation; IGF-1 rescue partially reversed viability, apoptosis-related proteins and pathway changes, supporting functional PI3K/AKT/mTOR involvement (pharmacological rescue | Causal/functional (*in vitro*)‡ | [71] |
| Icariside II (ICS II) | *Epimedium* (reported natural source); commercial ICS II, HPLC ≥98% | Human Caco-2 colorectal adenocarcinoma cells | Single 8 Gy irradiation (2 Gy/min); radiation source/type NR beyond study irradiation protocol | 5 μM ICS II added immediately after irradiation; outcomes assessed 24 h later | Reduced ROS and mitochondrial dysfunction; reduced apoptosis-related changes and restored tight-junction proteins | ICS II increased PINK1/Parkin/LC3-associated mitophagy markers; Mdivi-1 perturbation supported functional involvement of mitophagy, without genetic validation *In vitro* functional validation only | Causal/functional (*in vitro*)‡ | [75] |
| Licochalcone D (LCD) | Licorice (reported natural source); commercial LCD (TargetMol), purity NR | IEC-6 intestinal crypt epithelial cells | Single 12 Gy X-ray irradiation; dose rate NR | 0.1–5 μM screened; 0.5 μM selected; 2 h pretreatment before irradiation | Improved viability/clonogenic survival; reduced ROS/mtROS, DNA damage and apoptosis; preserved mitochondrial function | CQ abolished mitophagy-dependent radioprotection; SIRT3 siRNA abolished LCD-induced mitophagy, DNA protection, ROS reduction and cytoprotection, providing functional plus genetic evidence for SIRT3-dependent mitophagy *In vitro* functional validation | Causal/functional (*in vitro*)‡ | [77] |
| Madecassoside | *Centella asiatica* (reported natural source); commercial madecassoside (Aladdin), purity NR | Rat IEC-6 small-intestinal crypt epithelial cells | Single 10 Gy irradiation using a Siemens PRIMUS linear accelerator; 3 Gy/min | 0–2.0 μmol/L added after irradiation following medium replacement; 1.0 μmol/L selected; outcomes assessed on days 1, 3, 5, and 7 | Increased cell viability; reduced oxidative stress and pro-inflammatory cytokines; increased antioxidant-enzyme activity and IL-10 | Reduced NLRP3 mRNA only; associative evidence without inflammasome protein/activation assays or functional perturbation → expression/phenotype assays → Limit.: † | Pathway-level association† | [101] |
| Mangiferin aglycone (norathyriol) | NR (synthesized mangiferin aglycone; direct botanical source of the experimental material not reported) | Human intestinal epithelial cells (HIECs; ATCC CRL-1592) | 60Co γ-rays, 2 Gy/min; 0–16 Gy depending on assay (8 Gy viability/clonogenic screen; 4 Gy apoptosis | 0–100 μg/mL screened; 70 μg/mL selected; 1.5 h pretreatment before irradiation | Increased viability and clonogenic survival; reduced apoptosis/necrosis; reduced γH2AX foci/DNA double-strand breaks | Cell-free hydroxyl-radical scavenging and reduced radiation-induced DNA damage were demonstrated; descriptive/associative showed | Pathway-level association† | [102] |
| Perillaldehyde (PAH) | Perilla plants/*Perilla frutescens* (reported natural source); commercial PAH (AbMole), purity NR | Human HIEC-6 intestinal epithelial cells; mouse intestinal crypt organoids | HIEC-6: X-rays, 0–8 Gy screened; 6 Gy for main viability/ferroptosis assays and 2 Gy for γH2AX assay; organoids: single 6 Gy X-ray irradiation | HIEC-6: 100 μM PAH for 4 h before irradiation; ML385 10 μM used for Nrf2 perturbation | Increased viability/clonogenic survival; reduced DNA damage, cell death, ROS and lipid peroxidation; improved organoid budding/growth and Ki67-positive proliferation | PAH increased Nrf2/Slc7A11/GPX4-associated antioxidant signaling; ML385 partially/reversibly blocked radioprotection and ferroptosis suppression in HIEC-6 cells and organoids (functional pharmacologic evidence | Causal/functional (*in vitro*)‡ | [83] |
| *Pyrrosia petiolosa* aqueous extract | *Pyrrosia petiolosa* (Christ) Ching; aqueous decoction, botanically authenticated; LC–MS profiled | Mouse MODE-K intestinal epithelial cells | Single 12 Gy X-ray irradiation using RS 2000 Pro; 3.1 Gy/min | 0.1 mg/mL added after irradiation; post-IR; samples assessed at 6 and 24 h | Reduced IL-6/TNF-α/IL-1β expression and lipid peroxidation; restored GSH and SOD | Anti-inflammatory and antioxidant effects supported by cytokine mRNA and redox-marker changes only; associative evidence without direct pathway perturbation → redox assays, cytokine assay → Limit.: † | Pathway-level association† | [86] |
| Resveratrol | Commercial resveratrol (Sigma-Aldrich); specific botanical source NR | IEC-6 intestinal epithelial cells; species/origin NR in the article | High-energy 6-MeV X-rays, 0–10 Gy screened (0.67 Gy/min); exact dose used in downstream mechanistic assays NR in the main-text methods | 0.1–5 μM screened; 1 μM selected; 2 h pretreatment before irradiation | Increased post-irradiation viability; reduced ROS accumulation and apoptosis | Ex527 reversed resveratrol-associated reductions in ROS/apoptosis and altered FOXO3a/p53-related responses, supporting functional SIRT1 involvement; PI3K/AKT changes were not reversed by Ex527 and remain associative *In vitro* functional validation | Causal/functional (*in vitro*)‡ | [88] |
| *Rheum tanguticum* polysaccharides (RTP) | *Rheum tanguticum* Maxim. ex Regel rhizomes; polysaccharide preparation from the Air Force Military Medical University laboratory | Rat IEC-6 small-intestinal epithelial cells (CRL-1592; passage 14) | Single 10 Gy whole-dish X-ray irradiation (400 MU/min; source-to-cell distance 100 cm) | 5, 10, or 15 mg/mL RTP before irradiation; pre-IR; exact pretreatment interval NR | Increased cell viability; reduced ROS, apoptosis/dead cells and inflammatory responses after irradiation | Promoted Nrf2 nuclear translocation and increased Nrf2/HO-1 while reducing NF-κB, caspase-3 and inflammatory mediators; pathway-level association without inhibitor/rescue/genetic perturbation → pharmacologic inhibitor/activator → Limit.: † | Pathway-level association† | [93] |
| Rosavin (selected lead among *Rhodiola rosea* constituents) | *Rhodiola rosea* L. (reported natural source); commercial rosavin (Sigma), purity NR | Rat IEC-6 small-intestinal crypt epithelial cells (ATCC) | Single 10 Gy irradiation using a Siemens PRIMUS linear accelerator; 3 Gy/min | post-irradiation treatment with 0–100 μM for 72 h; 12.5 μM showed the strongest viability protection and was selected as the lead concentration | Increased viability of irradiated IEC-6 cells; rosavin showed the strongest protection among the tested *Rhodiola rosea* constituents | No mechanistic pathway was tested in irradiated IEC-6 cells; evidence was limited to cell-viability screening No direct pathway perturbation. → expression/phenotype assays → Limit.: † | Phenotypic/descriptive† | [94] |
| Salvianic acid A (SAA) | *Salvia miltiorrhiza* Bunge (reported constituent); commercial SAA purchased from Nanjing Zelang Bio-technique | Human normal intestinal epithelial HIEC cells | Single 4 Gy 60Co γ-irradiation; dose rate NR | 0.1, 1, or 10 mg/mL SAA, administered 1 h before irradiation; assay-specific concentrations used (1 mg/mL for comet assay) | Reduced micronucleus/comet/γH2AX DNA-damage readouts, ROS and apoptosis; partially preserved antioxidant status | Associated with lower p53/Bax, caspase-3/9 activity and mitochondrial cytochrome-c/AIF release, with higher Bcl-2; pathway-level/associative mitochondrial-apoptosis evidence without direct pathway perturbation → expression/phenotype assays → Limit.: † | Pathway-level association† | [103] |
| Sanguinarine (SAN) | Papaveraceae plants (reported general natural source); commercial sanguinarine chloride hydrate, 98% purity | IEC-6 small-intestinal epithelial cell line | X-rays (Rad Source Technologies); 4 Gy at 1.15 Gy/min for main viability/apoptosis assays; 0–8 Gy for clonogenic survival | 0.1 μM for 30 min before irradiation; drug-containing medium replaced immediately after irradiation in clonogenic assays | Improved cell viability and clonogenic survival; reduced radiation-induced early/late apoptosis | Reduced caspase-3/caspase-8 and NF-κB expression after irradiation; descriptive/pathway-level associative evidence post-IR | Pathway-level association† | [95] |
| Tea polyphenols (TP) | Tea (specific botanical species NR); TP mixture characterized by UPLC-Q-TOF/MS | Rat IEC-6 intestinal epithelial cells | 60Co γ-irradiation; 8 and 12 Gy used for oxidative-stress/ferroptosis assays; dose rate 92.88 R/min | post-irradiation treatment; 25–200 μg/mL tested; 50 μg/mL used for major ferroptosis/pathway assays, with assay-specific concentrations up to 150 μg/mL | Improved viability; reduced ROS/Fe2+ accumulation and mitochondrial injury; improved antioxidant indices and ferroptosis-related protein expression | TP restored Nrf2/HO-1/GPX4-associated signaling; HSP90 RNAi altered ferroptosis/pathway proteins, supporting HSP90 involvement but not TP dependence (no epistasis test) → Limit.: † | Pathway-level association† | [96] |
| WGX50 | *Zanthoxylum bungeanum* Maxim. (reported natural source); supplied WGX50 powder, purity NR | Human HIEC-6 intestinal epithelial cells; human NCM460 colonic epithelial cells | X-rays (Faxitron MultiRad 225); 6 Gy at 3 Gy/min for main assays; 0/2/4/6 Gy for colony formation | 20 μM selected; 24 h pretreatment before 6 Gy in the CCK-8 protocol; 72 h pretreatment before graded-dose colony assay | Improved proliferation/clonogenic survival; reduced apoptosis and ROS; restored mitochondrial membrane potential | SPR confirmed direct WGX50–EGFR binding (KD 5.86 μM); p-EGFR and SLC7A11/GPX4 increased with ferroptosis-related protection | Pathway-level association† | [98] |

Notes: Evidence categories are causal/functional (pathway-dependency perturbation in the relevant irradiated model), pathway-level association (target/pathway readouts without a dependency test), phenotypic/descriptive (outcomes without pathway validation), and indirect/contextual (nonirradiated or non-RE models). *In vitro* functional validation does not establish *in vivo* causality.

Common limitations: † no mechanism-dependency perturbation in the original article; ‡ *in vitro* functional validation is not equivalent to *in vivo* causality; § nonirradiated/non-RE models provide contextual rather than radioprotective evidence. Expression changes, omics, network pharmacology, and docking are hypothesis-generating unless functionally perturbed in the relevant irradiated model.

Abbreviations: DEG, differentially expressed gene; IR, irradiation; NR, not reported; post-IR, after irradiation; pre-IR, before irradiation; h, hour; d, day; wk, week; mo, month; HPLC, high-performance liquid chromatography; LC–MS, liquid chromatography–mass spectrometry. Symbols: ↑, increase/upregulation; ↓, decrease/downregulation; →, evidence chain.

**Reference**

1.	Liang W, Li B, Sun Y, Jia D, Hu T, Huang R, et al. Molecular mechanisms of the anchang group prescription in treating radiation enteritis: network pharmacology analysis and experimental evidence. Frontiers in pharmacology. 2025;16:1524925.

2.	Z Z, H W, A S, X Z, J Z, C Z, et al. Explore the mechanism of action of the combination of Astragali Radix and Scutellariae Radix in the intervention of acute adiation intestinal injury based on the “inflammation-gut microbiota” axis. China Medical Herald. 2025;22(30):26-32.

3.	Ying G, Yao W, Bai-ping A, Hai-liang J, Hai-ruo C, Wen-bo H, et al. Anti-inflammatory and anti-apoptotic effects of Baitouweng decoction on mice with radioactive enteritis. LISHIZHEN MEDICINE AND MATERIA MEDICA RESEARCH. 2022;33(04):827-30.

4.	W J, J L, Y D, L Y, J W. Influence of Baitouweng Decoction on Fibrogenic Factors and General Condition of Rats with Radiation Enteritis. Western Journal of Traditional Chinese Medicine. 2020;33(6):7-12.

5.	Yuming L, Sicong L. Effects of Changfukang Decoction on General Condition and Histomorphology on Small Intestinal Mucosa in Rats with Acute Radiation Enteritis. guiding journal of traditional chinese medicine and pharmacy. 2016;22(12):17-20.

6.	Lei W, Xuchun S, Xianlu S, Xiaopu W, Jiaxin K, Changbin X, et al. Changhejianye( CHJY) Can Activate Nrf2/ARE Signaling Pathway to Improve Radiation Enteritis in Rats. Liaoning Journal of Traditional Chinese Medicine. 2023;50(08):230-3+59.

7.	Ziyun X, Xuchun S, Jiaxin K, Shilei D, Ling C, Weichi J. Intervention Mechanisms of the NF-κB Signaling Pathway withChanghe Decoction in Rat Radiation Enterocolitis. Journal of Chinese Medicinal Materials. 2018;41(07):1731-4.

8.	Gao J, Li Y, Yang X, Hu M, Xu J, Cheng L, et al. Changrui enema inhibits inflammation-induced angiogenesis in acute radiation proctitis by regulating NF-κB and VEGF. Acta Cirurgica Brasileira. 2020;35(5):1-13.

9.	Zheng T, Shi X, Nie S, Yin L, Zhu J, Yu E, et al. Effects of Chinese herbal diet on hematopoiesis, immunity, and intestines of mice exposed to different doses of radiation. Heliyon. 2023;9(5):e15473.

10.	Xu W, Gao L, Zou W, Tang X, Nian W, Zheng W, et al. Compound kushen injection improves radiation enteritis via cannabinoid receptor 1 in rats. BMC complementary medicine and therapies. 2025;25(1):70.

11.	Y C, J S, Z L, Q L. Protective　Effects　of　Compound　Matrine　on　Small　Intestine　of　Rats　with　Acute　Radiation Enteritis. Cancer Research On Prevention And Treatmen. 2011;38(10):1129-32.

12.	Yu-guo W, Yong-qi D, Li Q. Effect of Daihuang Shaoyao powder on COX-2, TXB2 and 6-Keto-PGF1α in rats with radiation proctitis. Global Traditional Chinese Medicine. 2016;9(09):1043-6.

13.	Y W, L Q, Y D. Effect of Daihuang Shaoyao San on NF-κB, TNF-αand IL-6 in Rats with Radiation Proctitis. Guiding Journal of Traditional Chinese Medicine and Pharmacy. 2016;22(15):17-20.

14.	Y W, L Q, Y D. Efficacy Observation of Daihuang Shaoyao San in the Treatment of Radiation Proctitis in the Rats. World Journal of Integrated Traditional and Western Medicine. 2016;11(7):934-7+67.

15.	Y W, Y D, L Q. Effect of Daihuang Shaoyao San on Th1/Th2 in Rats with Radiation Proctitis. JOURNAL OF LIAONING UNIVERSITY OF TCM. 2017;19(2):16-9.

16.	 Liying Z,  Yangyang L,  Xin W,  Zhiming M,  Shangzu Z,  Yan C, et al. Study on the Protective Effect of the Dunhuang Medical Formula Dabupi Decoction on Rats with Radiation-induced Intestinal Edema. Chin J Mod Appl Pharm,. 2025;42(01):13-20.

17.	X D, D L, L Z. Therapeutic efficacy of fever- reducing and invigorating Chinese medicine in the treatment of patients with acute radiation enteritis and its effects on nitric oxide level and morphometrics in intestinal mucosa with radiation- induced enteritis in rats. Chinese Journal of Radiological Medicine and Protection. 2004(1):53-5.

18.	H C, F Y. Study on the Mechanism of Fuzheng Huoxue Qingre Formula in Radiative Rectal Injury in Rats. Shanxi Medical Journal. 2009;38(9):806-7.

19.	B D, S L, J Z. Study of Mechanism of Chinese Materia Medica in Radioactivitic Rectal Injury of Rat. CHINA MODERN DOCTOR. 2009;47(15):70-1.

20.	C L, X Y, S S. Effect of Modified Gegen Qinlian Decoction on SOD，CAT and MDA Expression Levels for Rats with Acute Radiation Proctitis. Journal of Sichuan of Traditional Chinese Medicine. 2020;38(1):55-8.

21.	Zhang M, Wang SJ, Du XQ, Li GA, Yu YY, Zhang YQ, et al. Decoding the healing secrets of Gegenqinlian decoction: A promising approach to radiation-induced intestinal injury. Journal of ethnopharmacology. 2026;354:120473.

22.	Li Y, Zhang L, Zhang Y, Miao Z, Liu Z, Zhou G, et al. Potential molecular mechanism of Guiqi Baizhu Decoction in radiation-induced intestinal edema by regulating HIF-1a, AQP4 and Na(+)/K(+)-ATPase. Phytomedicine : international journal of phytotherapy and phytopharmacology. 2022;107:154445.

23.	Shi LL, Chen YP, Guo HM, Wang XP, Li YY, Wang SZ, et al. Huanglian decoction prevents and treats radiation-induced intestinal injury in lung cancer by regulating endoplasmic reticulum stress. Journal of natural medicines. 2025;79(1):143-61.

24.	Jingyang S, Yue F, Menglei W, Jiang Y, Jue W, Shengyou L. Effect of Jiawei Xianglian Pills on NF-κB Signaling Pathway in Rats with Acute Radiation Enteritis. Journal of Emergency in Traditional Chinese Medicine. 2022;31(07):1153-6.

25.	Yu-guo W, Hong-ling T, Jian F, Yong-qi D. Regulation of Liangxue Guyuan decoction on intestinal microflora in rats with intestinal acute radiation injury. Military Medical Sciences. 2019;43(06):436-41+53.

26.	Yan Z, Yin B, Wang Y, Ni Z, Feng J, Yang Q, et al. Therapeutic mechanism of Liangxue-Guyuan-Yishen decoction on intestinal stem cells and tight junction proteins in gastrointestinal acute radiation syndrome rats. Journal of radiation research. 2023;64(6):880-92.

27.	Yan Z, Li Y, Xia T, Wang K, Liao Z, Zhang L, et al. Revitalizing gut health: Liangxue guyuan yishen decoction promotes akkermansia muciniphila -induced intestinal stem cell recovery post-radiation in mice. Phytomedicine : international journal of phytotherapy and phytopharmacology. 2024;132:155888.

28.	Yan Z, Li Y, Chang M, Xia T, Wang Y, Yu H, et al. Maintained homeostasis: LGYD facilitated the restoration of ISCs following radiation exposure by activating Hes1. Phytomedicine : international journal of phytotherapy and phytopharmacology. 2025;139:156506.

29.	Dou D, Lin Z, Jian W, Weisheng S, Huafang Y, Yihang G, et al. Curative effect and possible mechanism of Qinghua Liangxue Huluo decoction on rats with acute radiation enteritis. 2023;42(02):305-11.

30.	Yuanyuan Q, Lingyan Z, Li L, Bowen C, Zequn J, Mianhua W. The Impact of Qinghua Yichang Formula on NLRP3 Inflammasome in Intestinal Tissue of Mice with Acute Radiation-Induced Intestinal Injury Model. Journal of Traditional Chinese Medicine. 2024;65(16):1695-702.

31.	Chao W, Xin-ying H, Jian-qiang B, Yun-chuan S. Effects of Qingre Liangxue formula on the general condition and the expression of MDA and SOD in rats with acute radiation enteritis. Journal of Basic Chinese Medicine. 2020;26(03):338-40+88.

32.	W G, J R, Y Y, J X, H M. Activity and Mechanism of Qingre Zhixiefang in Treating Acute Radiation Enteritis in Rats. Herald of Medicine. 2013;32(7):878-81.

33.	Li Z, Gao Y, Du L, Yuan Y, Huang W, Fu X, et al. Anti-inflammatory and anti-apoptotic effects of Shaoyao decoction on X-ray radiation-induced enteritis of C57BL/6 mice. Journal of ethnopharmacology. 2022;292:115158.

34.	Z G, S H, Y H, X C, Y L, L F, et al. Chinese prescription Shenlingbaizhu Extract prevents radiation-induced small intestinal injury in mice. Military Medicine. 2011;35(10):769-72+79.

35.	C Y, Y J, J Y, Z Z, X W, Y L, et al. Protective effect of Tongxie Yaofang formula on intestinal tissue of rats with acute radiation enteritis. World Chinese Journal of Digestology. 2018;26(15):898-903.

36.	Yang C, Yang J, Xia M, Wei J, Jiao Y, Zhan Q. Efficacy of Compound Herbal Medicine Tong-Xie-Yao-Fang for Acute Radiation Enteritis and Its Potential Mechanisms: Evidence from Transcriptome Analysis. BioMed research international. 2020;2020:5481653.

37.	Wang YG, Gao Y, Feng J, Dou YQ. Effect of Modified Xijiao Dihuang Decoction on Intestinal Flora and Th17/Treg in Rats with Radiation Enteritis. Chinese journal of integrative medicine. 2021;27(3):198-205.

38.	W H, J Z, R D, Y Z, Z L, F X, et al. Effects of Xilei powder and Yunnan Baiyao gel on TNF-α and IL-6 in rats with acute radiation proctitis. World Journal of Integrated Traditional and Western Medicine. 2019;14(10):1392-5.

39.	Dong Y, Zhang Y, Wang X, Li W, Zhang J, Lu L, et al. The protective effects of Xuebijing injection on intestinal injuries of mice exposed to irradiation. Animal models and experimental medicine. 2022;5(6):565-74.

40.	Li Y, Lu D, Zhou L, Zhang Y, Yang C, Liu S, et al. Yinhuo Tang alleviates ionizing radiation injury via modulating PI3K-Akt signaling and metabolomics in mice. Journal of ethnopharmacology. 2026;356:120843.

41.	Wang J, Yao M, Wang Y, Ho C-T, Li S, Shi Y, et al. 6-Shogaol ameliorates injury to the intestinal mucosa and increases survival after high-dose abdominal irradiation. Journal of Functional Foods. 2017;36:63-71.

42.	Park E, Hwang I, Song JY, Jee Y. Acidic polysaccharide of Panax ginseng as a defense against small intestinal damage by whole-body gamma irradiation of mice. Acta histochemica. 2011;113(1):19-23.

43.	Zeng X, Pang S, Lu H, Zhu J, Li Z, Liu N, et al. Aloperine ameliorates radiation enteritis by driving the TAS2R138-PLCβ/GAPDH axis-mediated glucose metabolic reprogramming to inhibit ferroptosis in intestinal epithelial cells. Biochemical pharmacology. 2025;242(Pt 1):117292.

44.	Haiping Y, Lianying Y, Xiaohua C, Qinglian Z. The  anti-inflammatory, antifibrotic and antibacterial function of Amentoflavone in radiation proctitis  via  NF-κB  pathway. Chin  J  Gastroenterol  Hepatol. 2023;32(04):367-73+78.

45.	Liu D, Peng R, Chen Z, Yu H, Wang S, Dong S, et al. The Protective Effects of Apigenin Against Radiation-Induced Intestinal Injury. Dose-response : a publication of International Hormesis Society. 2022;20(3):15593258221113791.

46.	Ji-Yuan H, Guo-Yong S, Zhe G, Chun-Li Z. Study on the Repairing Effect of Astragalus Polysaccharide on Intestinal Mucosal Injury in Rats with Radiation Enteritis. Journal of Guangzhou University of Traditional Chinese Medicine. 2022;39(07):1630-6.

47.	Xiao C, Xu C, He N, Liu Y, Wang Y, Zhang M, et al. Atractylenolide II prevents radiation damage via MAPKp38/Nrf2 signaling pathway. Biochemical pharmacology. 2020;177:114007.

48.	Jang H, Lee J, Park S, Kim JS, Shim S, Lee SB, et al. Baicalein Mitigates Radiation-Induced Enteritis by Improving Endothelial Dysfunction. Frontiers in pharmacology. 2019;10:892.

49.	Wang M, Dong Y, Wu J, Li H, Zhang Y, Fan S, et al. Baicalein ameliorates ionizing radiation-induced injuries by rebalancing gut microbiota and inhibiting apoptosis. Life sciences. 2020;261:118463.

50.	Tu S, Huang Y, Tian H, Xu L, Wang X, Huang L, et al. Berberine enhances the function of intestinal stem cells in healthy and radiation-injured mice. International immunopharmacology. 2024;136:112278.

51.	Yao W, Ying G, Hai-Ruo C, Hai-Liang J, Zhuo-Hong L. Curative effect and possible mechanism of berberine on rats with acute radiation enteritis. Med J Chin PLA. 2021;46(10):968-74.

52.	Kim HB, Kim H, Oh SH, Kang MJ, Park JH, Lee SB, et al. Bixin alleviates radiation-induced intestinal damage via inflammation regulation and barrier recovery. International journal of radiation biology. 2025;101(7):720-9.

53.	Korany DA, Said RS, Ayoub IM, Labib RM, El-Ahmady SH, Singab ANB. Protective effects of Brownea grandiceps (Jacq.) against ϒ-radiation-induced enteritis in rats in relation to its secondary metabolome fingerprint. Biomedicine & pharmacotherapy = Biomedecine & pharmacotherapie. 2022;146:112603.

54.	Chen C, Chen Z, Xu F, Zhu C, Fang F, Shu S, et al. Radio-protective effect of catalpol in cultured cells and mice. Journal of radiation research. 2013;54(1):76-82.

55.	Kwak SY, Jang WI, Lee SB, Kim MJ, Park S, Cho SS, et al. Centella asiatica-Derived Endothelial Paracrine Restores Epithelial Barrier Dysfunction in Radiation-Induced Enteritis. Cells. 2022;11(16).

56.	Chen H, Wang Y, Xu Y, Liu F, Xu Q, Dong L, et al. Crocetin curbs radiation induced intestinal injury by blocking KAT2A/NLRP3 succinylation. Scientific reports. 2025;15(1):36753.

57.	Akpolat M, Kanter M, Uzal MC. Protective effects of curcumin against gamma radiation-induced ileal mucosal damage. Archives of toxicology. 2009;83(6):609-17.

58.	Xu J, Yang X, Pu K, Zhang Q, Wei Q, Ma X, et al. Cynaroside alleviates radiation-induced intestinal injury by inhibiting dynamin 2. International immunopharmacology. 2025;162:115167.

59.	Liu X, Zhang Y, Wang Z, Wei Y, Wu Q, Zhu J. Dendrobium huoshanense flavone attenuates radiation enteritis by enhancing gut immunity to inhibit inflammation and fibrosis. Phytomedicine : international journal of phytotherapy and phytopharmacology. 2025;148:157364.

60.	Zeng X, Zhang L, He J, Zhang Y, Lu H, Liu Z, et al. Ecliptasaponin II alleviates the radiation enteritis by activating PPARγ to inhibit the apoptosis of endothelial cells. International immunopharmacology. 2025;161:115080.

61.	Dong-zhi Z, Wen L, Yue-jun L, Xin-xin X, Yu W, Yong-hui L. Therapeutic effect and anti-free radical injury of emodin on acute radiation enteritis. JOURNAL OF SHANDONG UNIVERSITY( HEALTH SCIENCES). 2011;49(06):76-9.

62.	Li Y, Ma S, Zhang Y, Yao M, Zhu X, Guan F. (-)-Epicatechin mitigates radiation-induced intestinal injury and promotes intestinal regeneration via suppressing oxidative stress. Free radical research. 2019;53(8):851-64.

63.	Cai S, Xie L-W, Xu J-Y, Zhou H, Yang C, Tang L-F, et al. (-)-Epigallocatechin-3-Gallate (EGCG) Modulates the Composition of the Gut Microbiota to Protect Against Radiation-Induced Intestinal Injury in Mice. 2022;Volume 12 - 2022.

64.	Xie LW, Cai S, Zhao TS, Li M, Tian Y. Green tea derivative (-)-epigallocatechin-3-gallate (EGCG) confers protection against ionizing radiation-induced intestinal epithelial cell death both in vitro and in vivo. Free radical biology & medicine. 2020;161:175-86.

65.	Moon C, Ahn K, Kim J, Kim J, Kim SH, Oh TH, et al. Eutigoside C attenuates radiation-induced crypt injury in the mouse intestine. Phytotherapy research : PTR. 2010;24(6):840-5.

66.	Yang C, Yuhua G, Mingxin Y. Therapeutic Effect of Evodiamine on Acute Radiation Enteritis and Possible Mechanisms. JOURNAL OF LIAONING UNIVERSITY OF TCM. 2017;19(02):29-31.

67.	PANGZhijun, Zhaowei W, Chuanyang X, Xutao Z, HELei, Meng L, et al. Protective effect of fructus sophorae genistein on radiation-induced intestinal injury. Journal of Radiation Research and Radiation Processing. 2019;37(01):36-43.

68.	Zhang J, Pang Z, Zhang Y, Liu J, Wang Z, Xu C, et al. Genistein From Fructus sophorae Protects Mice From Radiation-Induced Intestinal Injury. Frontiers in pharmacology. 2021;12:655652.

69.	Son TG, Gong EJ, Bae MJ, Kim SD, Heo K, Moon C, et al. Protective effect of genistein on radiation-induced intestinal injury in tumor bearing mice. BMC Complement Altern Med. 2013;13:103.

70.	Ting-ting H, Li-li L, Xiao-yu D, Hong-y C. Investigation of the mechanism of ginsenoside Rg3 in the treatment of radiation proctitis in rats. China Journal of Modern Medicine. 2024;34(21):34-42.

71.	Wang Y, Su P, Zhuo Z, Jin Y, Zeng R, Wu H, et al. Ginsenoside Rk1 attenuates radiation-induced intestinal injury through the PI3K/AKT/mTOR pathway. Biochemical and biophysical research communications. 2023;643:111-20.

72.	Zhang XM, Hu X, Ou JY, Chen SS, Nie LH, Gao L, et al. Glycyrrhizin Ameliorates Radiation Enteritis in Mice Accompanied by the Regulation of the HMGB1/TLR4 Pathway. Evidence-based complementary and alternative medicine : eCAM. 2020;2020:8653783.

73.	Guven B, Can M, Piskin O, Aydin BG, Karakaya K, Elmas O, et al. Flavonoids protect colon against radiation induced colitis. Regulatory toxicology and pharmacology : RTP. 2019;104:128-32.

74.	Liu L, Lu X, Li SZ, Zhang P. Polysaccharides isolated from Hibiscus sabdariffa L. mitigate intestinal radiation injury through relieving mucosal inflammation and reshaping gut microbiota in mice. JOURNAL OF FUNCTIONAL FOODS. 2024;121.

75.	He S, Yan C, Wang Z, Mao Y, Liu K, Sun J, et al. Icariside II relieves radiation enteritis by regulating PINK/Parkin-mediated mitophagy. International immunopharmacology. 2025;146:113861.

76.	Chen YP, Shi LL, Li YY, Zhang YM, Zhang SZ, Hou HD, et al. Isoliquiritigenin alleviates radiation-induced intestinal injury in lung cancer by inhibiting TNF-α/caspase3 signaling pathway. Naunyn-Schmiedeberg's archives of pharmacology. 2025;398(9):12353-68.

77.	Zhang J, Ma S, Wang Z, Wang P, Liu H, Wang Q. Licochalcone D Alleviates Radiation-Induced Intestinal Injury via SIRT3-Dependent Mitophagy: A Natural Phytochemical Strategy for Radioprotection. Journal of agricultural and food chemistry. 2025;73(41):26035-47.

78.	Li J, Zheng X, Li X, Yang J, Liu W, Yang L, et al. Study on the protective effect and mechanism of Liriodendrin on radiation enteritis in mice. Journal of radiation research. 2022;63(2):213-20.

79.	Huang H, Li J, Zhang Y, Liu B, Jia G, Zhao G. Liriodendron attenuates intestinal fibrosis and inflammation in mice with radiation proctopathy. Chinese medicine. 2025;20(1):181.

80.	Lei Z, Zhengjie W, Wenbo L, Jia L, Huan H, Ying W, et al. Lycium barbarum polysaccharide inhibits apoptosis of intestinal epithelial cells through mitochondrial pathway in mice with radiation-induced intestinal injury. Journal of Third Military Medical University. 2020;42(20):1987-94.

81.	Li H, Du L, Lu Y, Cheng X, Xiao F, Zhang Q. Effects of Orychophragmus violaceus extract OVS-2 on the structure and diversity of intestinal flora in mice with radiation intestinal injury. Biomedical Research and Therapy. 2024;11(9):6764-74.

82.	Li YH, He Q, Chen YZ, Du YF, Guo YX, Xu JY, et al. p-Coumaric acid ameliorates ionizing radiation-induced intestinal injury through modulation of oxidative stress and pyroptosis. Life sciences. 2021;278:119546.

83.	Tang LF, Ma X, Xie LW, Zhou H, Yu J, Wang ZX, et al. Perillaldehyde Mitigates Ionizing Radiation-Induced Intestinal Injury by Inhibiting Ferroptosis via the Nrf2 Signaling Pathway. Molecular nutrition & food research. 2023;67(19):e2300232.

84.	Zhenkui Z, Jiarui H, Shuling J, Lulu H, Xiaoyu D, Sa F, et al. Use of puerarin to alleviate radiation-induced acute intestinal mucosal injury in rats. CHINESE JOUＲNAL OF COMPAＲATIVE MEDICINE. 2021;31(06):41-7.

85.	Cho HT, Kim JH, Heo W, Lee HS, Lee JJ, Park TS, et al. Explosively Puffed Ginseng Ameliorates Ionizing Radiation-Induced Injury of Colon by Decreasing Oxidative Stress-Related Apoptotic Cell Execution in Mice. J Med Food. 2019;22(5):490-8.

86.	Yang H, Zhu H, Yan X, Liu Y, Chen Y, Wang J, et al. Metabolomic and Microbiome Profiling Reveals the Protective Mechanism of Pyrrosia petiolosa Against Radiation-Induced Intestinal Injury. International journal of molecular sciences. 2026;27(12).

87.	Liu TH, Sheng YY, Zhang CY, Zhang H, Li X, Yu LH, et al. Common key ingredient concluded in Baitouweng decoction, Gegen Qinlian decoction and Shaoyao decoction prevents radiation enteritis by regulating gut microbial network. Traditional Medicine Research. 2024;9(5).

88.	Qin H, Zhang H, Zhang X, Zhang S, Zhu S, Wang H. Resveratrol attenuates radiation enteritis through the SIRT1/FOXO3a and PI3K/AKT signaling pathways. Biochemical and biophysical research communications. 2021;554:199-205.

89.	Radwan RR, Karam HM. Resveratrol attenuates intestinal injury in irradiated rats via PI3K/Akt/mTOR signaling pathway. Environmental toxicology. 2020;35(2):223-30.

90.	Sun H, Cai H, Fu Y, Wang Q, Ji K, Du L, et al. The Protection Effect of Resveratrol Against Radiation-Induced Inflammatory Bowel Disease via NLRP-3 Inflammasome Repression in Mice. Dose-response : a publication of International Hormesis Society. 2020;18(2):1559325820931292.

91.	Sha H, Gu Y, Shen W, Zhang L, Qian F, Zhao Y, et al. Rheinic acid ameliorates radiation-induced acute enteritis in rats through PPAR-γ/NF-κB. Genes & genomics. 2019;41(8):909-17.

92.	Linn L, Tian Z, Lei S, Zhipei Z, Shicao L, Bo G, et al. Protective role of Ｒheum tanguticum polysaccharide 1 on the radiation-induced acute intestinal mucosal injury. ChinJGastroenterol Hepatol. 2015;24(04):414-7.

93.	Zhang T, Shi L, Li Y, Mu W, Zhang H, Li Y, et al. Polysaccharides extracted from Rheum tanguticum ameliorate radiation-induced enteritis via activation of Nrf2/HO-1. Journal of radiation research. 2021;62(1):46-57.

94.	Zhou W, Chen K, Lu Q, Luo Y, Zhang C, Zheng Y, et al. The Protective Effect of Rosavin from Rhodiola rosea on Radiation-Induced Intestinal Injury. Chemistry & biodiversity. 2020;17(12):e2000652.

95.	Gu J, Zhao L, Chen YZ, Guo YX, Sun Y, Guo Q, et al. Preventive effect of sanguinarine on intestinal injury in mice exposed to whole abdominal irradiation. Biomedicine & pharmacotherapy = Biomedecine & pharmacotherapie. 2022;146:112496.

96.	Li R, Li L, Wu H, Gan H, Wu Z, Gu R, et al. Tea Polyphenols Mitigate Radiation-Induced Ferroptosis and Intestinal Injury by Targeting the Nrf2/HO-1/GPX4 Signaling Pathway. Antioxidants (Basel, Switzerland). 2025;14(5).

97.	Hou Q, Liu L, Dong Y, Wu J, Du L, Dong H, et al. Effects of Thymoquinone on radiation enteritis in mice. Scientific reports. 2018;8(1):15122.

98.	Yin Z, Chen G, Liu Y, Tan Y, Tang J, Zhang G, et al. WGX50 attenuates radiation enteritis by targeting ferroptosis and redox homeostasis via EGFR. Molecular medicine (Cambridge, Mass). 2025;31(1):309.

99.	Zhang C, Chen K, Wang J, Zheng Z, Luo Y, Zhou W, et al. Protective Effects of Crocetin against Radiation-Induced Injury in Intestinal Epithelial Cells. BioMed research international. 2020;2020:2906053.

100.	Yuguo W, Yongqi D, Sen Z. Protective effect of ginsenoside Rg1 on intestinal IEC-6 cell injury induced by radiation: An in vitro experiment. Academic Journal of Chinese PLA Medical School. 2020;41(03):284-8+93.

101.	Gan M, Jie Y, Qi Y. Madecassoside Ｒepair of radiation-induced damage to small intestinal epithelial cells. ModernDigestion ＆ Intervention. 2021;26(11):1390-4+9.

102.	Lei J, Zhou C, Hu H, Hu L, Zhao M, Yang Y, et al. Mangiferin aglycone attenuates radiation-induced damage on human intestinal epithelial cells. Journal of cellular biochemistry. 2012;113(8):2633-42.

103.	Zhang Y, Guo J, Qi Y, Shao Q, Liang J. The prevention of radiation-induced DNA damage and apoptosis in human intestinal epithelial cells by salvianic acid A. Journal of Radiation Research and Applied Sciences. 2014;7(3):274-85.
